# On‐the‐Fly Synthesis of Freestanding Spin‐Crossover Architectures With Tunable Magnetic Properties

**DOI:** 10.1002/adma.202420492

**Published:** 2025-06-13

**Authors:** Anh Tuan Ngo, David Aguilà, João Pedro Vale, Semih Sevim, Michele Mattera, Jordi Díaz‐Marcos, Ramon Pons, Guillem Aromí, Bumjin Jang, Salvador Pané, Tiago Sotto Mayor, Mario Palacios‐Corella, Josep Puigmartí‐Luis

**Affiliations:** ^1^ Departament de Ciència dels Materials i Química Física and Institut de Química Teòrica i Computacional Universitat de Barcelona Diagonal 645 Barcelona 08028 Spain; ^2^ Departament de Química Inorgànica i Orgànica Universitat de Barcelona Diagonal 645 Barcelona 08028 Spain; ^3^ Institut de Nanociència i Nanotecnologia (IN2UB) Universitat de Barcelona Barcelona 08007 Spain; ^4^ Associate Laboratory in Chemical Engineering (ALICE) Engineering Faculty of Porto University Porto 4200‐465 Portugal; ^5^ Multi‐Scale Robotics Lab Institute of Robotics & Intelligent Systems ETH Zurich Tannenstrasse 3 Zurich 8092 Switzerland; ^6^ Unitat de Tècniques Nanomètriques Centres Científics i Tecnològics de la Universitat de Barcelona (CCiTUB) Carrer de Lluís Solé i Sabarís, 1, Les Corts Barcelona 08028 Spain; ^7^ Institute for Advanced Chemistry of Catalonia (IQAC‐CSIC) Jordi Girona 18–26 Barcelona 08034 Spain; ^8^ Hanyang University ERICA 55, Hangyangdaehak‐ro, Sangrok‐gu Ansan‐si Gyeonggi‐do 15588 South Korea; ^9^ Institució Catalana de Recerca i Estudis Avançats (ICREA) Pg. Lluís Companys 23 Barcelona 08010 Spain; ^10^ Transport Phenomena Research Centre (CEFT) Engineering Faculty of Porto University Porto 4200‐465 Portugal

**Keywords:** 3D flow focusing, controlled concentration gradients, functional composites, hybrid composites, printing

## Abstract

Spin‐crossover (SCO) molecular‐based switches have shown promise across a range of applications since their discovery, including sensing, information storage, actuators, and displays. Yet limited processability remains a barrier to their real‐world implementation, as traditional methods for integrating SCO materials into polymer matrices are often complex, expensive, and prone to producing uneven material distributions. Herein, we demonstrate how 3D flow‐focusing chemistry enables unprecedented control for the direct fabrication of SCO composite materials, addressing key challenges in processability, scalability, and cost. By using a 3D coaxial flow‐focusing microfluidic device, we simultaneously synthesize [Fe(Htrz)_2_(trz)](BF_4_) and achieve its homogeneous incorporation into alginate fibers in a continuous manner. The device’s versatility allows for precise manipulation of the reaction‐diffusion (RD) zone, resulting in SCO composite fibers with tunable physicochemical and magnetic properties. Additionally, we demonstrate the ability to isolate these fibers as freestanding architectures and highlight the potential for printing them with defined shapes. Finally, we show that the 3D control of the RD zone granted by continuous flow microfluidic devices offers precise spatiotemporal control over the distribution of SCO complexes within the fibers, effectively encoding SCO materials into them. SCO‐encoded fibers can seamlessly combine adaptability and functionality, offering innovative solutions for application‐specific customization.

## Graphical abstract

3D flow‐focusing devices are an ideal platform for the processing and integration of functional materials within polymeric matrices. In this article, A. Tuan Ngo, D. Aguilà, T. Sotto Mayor, M. Palacios‐Corella, J. Puigmartí‐Luis show the on‐the‐fly processing of a prototypical SCO material—known for its challenging processability—thereby opening new avenues for its implementation in real‐world applications.

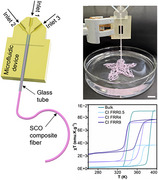

## 1 Introduction

The dynamic interplay between low‐spin (LS) and high‐spin (HS) configurations in spin‐crossover (SCO) materials is one of the most remarkable phenomena in molecular magnetic switches. This bistability—induced by temperature, pressure, light, or guest molecules^[^ ^1^, ^2^, ^3^, ^4^ ^]^ —originates from an electron redistribution between molecular orbitals, giving rise to pronounced changes in magnetic, optical, mechanical, and structural properties. These rich physicochemical signatures endow SCO materials with exceptional multifunctionality and readout versatility, positioning them as prime candidates for advanced applications including magnetic memories,^[^ ^5^ ^]^ sensors,^[^ ^6^, ^7^ ^]^ actuators,^[^ ^8^ ^]^ and displays.^[^ ^9^ ^]^

Despite their immense potential, the technological translation of SCO materials remains severely constrained by the formidable challenge of processing them directly onto surfaces or integrating them into processable matrices. Traditional approaches like chemical vapor deposition (CVD)^[^ ^10^ ^]^ are constrained by the thermal fragility of SCO complexes, while wet‐grafting or layer‐by‐layer assembly methods are laborious, limited to few‐layer thin films, and their scalability for covering larger surfaces remains uncertain.^[^ ^11^, ^12^, ^13^, ^14^ ^]^ Recent strategies have shifted toward in situ processing on nanostructured supports such as 2D materials^[^ ^15^ ^]^ or Au nanoparticles (NPs),^[^ ^16^ ^]^ revealing intriguing interfacial synergies, such as strain‐induced phase transitions in MoS₂^[^ ^15^ ^]^ or enhanced electrical outputs on Au.^[^ ^16^ ^]^ Nonetheless, while feasible, this direct processing usually yields small amounts of material, limiting their scalability.^[^ ^17^ ^]^

To circumvent traditional methods, where a tradeoff between scalability and processability is faced, SCO materials have been integrated with a variety of processable fibrous materials, such as cellulose,^[^ ^18^ ^]^ chitosan, and alginate.^[^ ^19^, ^20^ ^]^ Producing such hybrid composites has largely relied on diverse protocols that involve mixing solutions (or suspensions) of their two components^[^ ^19^, ^20^, ^21^, ^22^, ^23^, ^24^, ^25^, ^26^, ^27^, ^28^, ^29^, ^30^, ^31^, ^32^, ^33^ ^]^ or dispersing previously synthesized SCO particles in the polymer.^[^ ^8^, ^34^, ^35^, ^36^, ^37^, ^38^, ^39^, ^40^, ^41^, ^42^, ^43^, ^44^, ^45^, ^46^, ^47^ ^]^ However, these uncontrollable, non‐optimized, and often multistep methods lead to an inhomogeneous particle distribution within the polymer matrix. Therefore, further efforts are needed to develop robust and scalable methodologies that can simultaneously enable: i) the fabrication of SCO composite materials via the integration of SCO compounds onto a polymer matrix in a rapid, controllable, reproducible, and direct way, ii) the tuning of the magnetic properties of the SCO composite materials through simple modifications on the fabrication parameters, and iii) the production of freestanding SCO composite architectures.

Microfluidic techniques offer an elegant solution to these challenges. Operating in the low Reynolds number regime, microfluidic systems enable exquisite control over reaction‐diffusion (RD) processes—dictating when, where, and how reaction components interact simply by tuning flow conditions.^[^ ^48^, ^49^ ^]^ This control has been demonstrated to be paramount for obtaining materials with unprecedented physicochemical properties.^[^ ^50^, ^51^, ^52^ ^]^ For instance, recently, some of us harnessed this capability to access a non‐equilibrium crystalline phase of the canonical iron‐triazole [Fe(Htrz)_2_(trz)](BF_4_) coordination polymer, which exhibited a markedly distinct spin‐transition behavior compared to its bulk‐synthesized counterpart.^[^ ^53^ ^]^ Beyond pathway control, microfluidic systems offer untapped potential for embedding functional materials within freestanding polymeric matrices in a reproducible and scalable manner.^[^ ^54^ ^]^ Yet, despite all these possibilities, microfluidic methodologies remain largely underexplored for the fabrication of SCO–polymer composite architectures.

Herein, we leverage a microfluidic approach that provides 3D control over an RD zone, enabling the continuous and reproducible fabrication of SCO composite fibers via the in situ synthesis and integration of the well‐known iron‐triazole complex [Fe(Htrz)_2_(trz)](BF_4_)^[^ ^55^ ^]^ within a polymeric matrix. Taking advantage of the advanced design of the microfluidic chip, we modify different reaction parameters—including the injection position of the chemical precursors and their relative flow rates (controlled by the flow rate ratio, FRR)—to fine‐tune the RD zone and achieve SCO composite fibers with tailored physicochemical and magnetic properties. Moreover, we explore the potential for creating freestanding architectures and for printing complex geometries. Finally, we demonstrate how further advancements in chip design offer spatiotemporal control over the SCO complex distribution within the fiber matrix, expanding the possibilities for application‐specific customization.

## 2 Results and Discussion

Precisely controlling RD processes in microfluidic devices enables unconventional reaction pathways in material synthesis. This control facilitates the generation of unprecedented out‐of‐equilibrium crystal habits and compounds with unique physicochemical properties that differ significantly from those produced through conventional turbulent mixing.^[^ ^48^, ^49^, ^56^, ^57^, ^58^ ^]^ Building on this understanding, and before exploring into the formation of the SCO composite fibers, we first evaluated how the continuous flow microfluidic device operating with 3D control over the RD zone can affect the physicochemical properties of the SCO complex, compared to its bulk counterpart generated under turbulent conditions.^[^ ^55^ ^]^ To this end, we carried out the synthesis of [Fe(Htrz)_2_(trz)]BF_4_ without the polymeric matrix in the 3D coaxial flow‐focusing microfluidic device.

**Figure 1:**
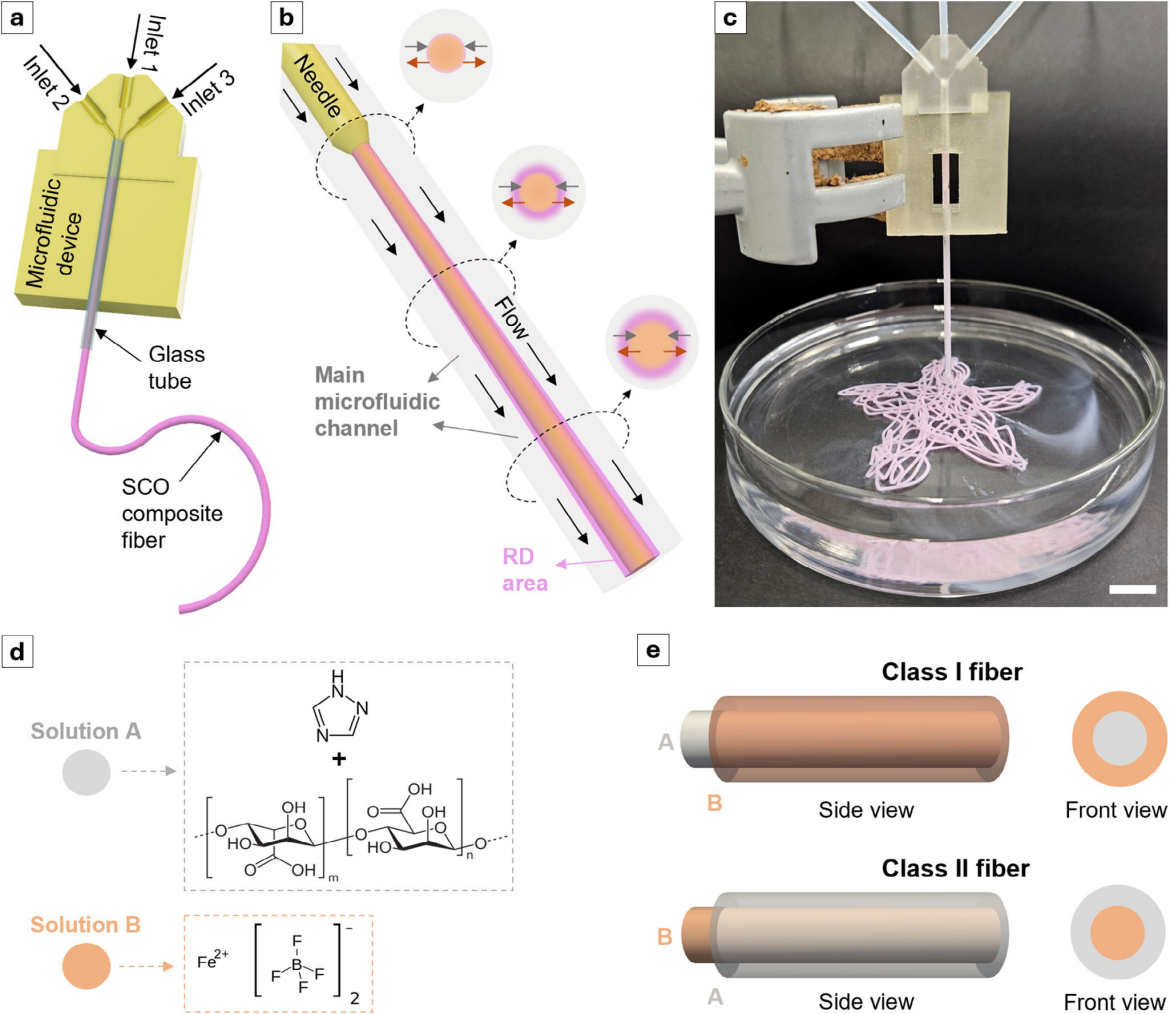
a) Schematic representation of the 3D flow‐focusing microfluidic device. b) Illustration of the gradual development of the RD zone, which takes place downstream along the main microfluidic channel. c) Photograph of the microfluidic device while generating SCO composite fiber to print a flower pattern—scale bar: 10 mm. d) Illustration of the two solutions used to generate the SCO composite fibers. Solution A is a mixture of 1,2,4‐triazole (Htrz) and sodium alginate in water, while solution B is the ethanolic solution of iron(II) tetrafluoroborate (Fe(BF_4_)_2_). e) Illustrations showing the flow configurations to generate the two types of SCO composite fibers. For Class I fibers, solution A is flowed through the central inlet, while solution B is introduced through the side inlets. In contrast, Class II fibers are generated by flowing solution B through the central inlet and solution A through the side ones.

The microfluidic device consists of three‐inlet channels—one central channel (inlet 1) for the central flow and two side channels (inlets 2 and inlet 3) for the sheath flows—and one outlet where the product is released (**Figure** **1**a; Figure S1a, Supporting Information). This inlet configuration facilitates a coaxial integration of the central flow within the sheath flows and a concentric arrangement of the RD zone at the liquid–liquid interface between the central flow and the sheath flow (Figure 1b). This flow configuration can enable precise control over reagent diffusion along the main microfluidic channel by adjusting the FRR (FRR = sheath flow rate /central flow rate). The validity of this approach is demonstrated using numerical simulations (see Section S2, Supporting Information).

Owing to the flow configuration, two distinct materials termed “Class I particles,” and “Class II particles” were prepared, depending on the injection position of the chemical precursors (Figure 1d,e; Figure S2 and Table S1, Supporting Information). Class I particles were synthesized using a total flow rate (TFR, sum of all flow rates) of 750 µL min^−1^ and a FRR of 4 by injecting an ethanolic solution of Fe(BF_4_)_2_ (0.4 m) in the side inlet channels at a flow rate of 300 µL min^−1^ and an aqueous solution of Htrz (2 m) in the central inlet channel at a flow rate of 150 µL min^−1^. Meanwhile, Class II particles were generated keeping the TFR and FRR constant but switching the injection position of the precursors. Thus, the Htrz solution was injected into the side inlet channels at a flow rate of 300 µL min^−1^, while the Fe(BF_4_)_2_ solution was introduced through the central inlet channel at a flow rate of 150 µL min^−1^. By changing the position of the reagents while keeping the other parameters (TFR and FRR) constant, we expected to see important physicochemical differences in the formation of the [Fe(Htrz)_2_(trz)]BF_4_ particles owing to the different concentration profiles and radial diffusion of the chemical precursors inside the microfluidic device (Figures S25 and S26, Supporting Information). The TFR and FRR values used in these syntheses were optimized to grow the RD zone within the main microfluidic channel as fast as possible to maximize the reaction time (Figure S27, Supporting Information), while simultaneously enabling the large‐scale production of SCO particles at industrially relevant quantities (≈ 8 g/day in a single device, Table S2, Supporting Information).^[^ ^59^ ^]^ It is important to note that too low TFR and FRR can cause clogging of the microfluidic device, whereas too high TFR and FRR will shorten the RD zone (Figure S27, Supporting Information), reducing the continuous production of SCO particles and the time available for the proper stoichiometric formation of the SCO complex (see, Section S2 and Table S2, Supporting Information).

Regardless of the class of the particles being formed, the [Fe(Htrz)_2_(trz)]BF_4_ materials were isolated from the microfluidic device as solid particles with a purple hue. We evaluated the morphology and size of Class I and II particles using scanning electron microscopy (SEM). Clear morphological differences become apparent when comparing the two configurations: Class I particles exhibit a rod‐like shape with an average diameter of 293 nm, whereas Class II particles display both spherical and elongated shapes with a smaller average size of 195 nm (Figure S3, Supporting Information).

To explain these differences, it is essential to consider several aspects. First, the diffusion coefficient of Fe(II) is approximately half that of Htrz (Table S6, Supporting Information). This means that, regardless of the FRR and precursor injection position (central or side), Htrz will diffuse throughout the entire cross‐section of the channel, while Fe(II), due to its lower diffusion coefficient, will only do so when it acts as the focusing flow or when it is weakly focused (see numerical simulation results in, Section S2 and Figure S26, Supporting Information). The second key factor is that the flow configuration controls the relative amounts of each reagent introduced. For instance, at a FRR of 4, the molar flow (flow rate multiplied by concentration) of Fe(II) injected into the channel differs drastically from one class to the other—0.00024 mol min^−1^ in Class I versus 0.00006 mol min^−1^ in Class II, i.e., a 4‐fold difference. Thus, depending on the conditions, Fe(II) may be either in excess or in deficit of Htrz (and vice versa), relative to the stoichiometric 1:3 Fe:Htrz ratio required for the formation of the SCO complex. Furthermore, taking the aspects of diffusion and stoichiometry into account, the diffusion of precursors within the main channel can still result in a non‐stoichiometric distribution within the RD zone even with the precursor solutions injected at the correct stoichiometric ratio, potentially hindering the proper formation of SCO particles if Fe(II) does not diffuse sufficiently.

Based on these considerations and the SEM images, we conclude the following: in Class I particle synthesis, an excess of Fe(II) in the main channel, along with an RD zone that spans the entire channel (Figures S27, S28 and S30, Supporting Information), facilitates continuous particle formation and larger particle growth. In contrast, Class II particle formation involves introducing a twenty‐fold excess of Htrz (the focusing flow or sheath flow) relative to Fe(II) into the channel. This configuration creates an RD zone confined to the region where Fe(II) diffuses (Figures S27, S29, and S30, Supporting Information). Although the restricted RD zone theoretically facilitates stoichiometric balance within its boundaries, the significantly low availability of Fe(II) ultimately limits particle growth. As a result, the particles formed in Class II are smaller than those in Class I. In terms of morphology, an excess of Fe(II) in Class I appears to promote the formation of rod‐shaped particles, whereas a deficiency in Fe(II) leads to incomplete rod shaping, resulting in rounded particles. To the best of our knowledge, rod‐shaped structures are the preferred morphology during [Fe(Htrz)_2_(trz)]BF_4_ synthesis across various synthetic techniques,^[^ ^60^, ^61^ ^]^ although nanocubes can also be achieved under specific conditions.^[^ ^62^ ^]^ Thus, we attribute the incomplete shaping in Class II particles to insufficient Fe(II) availability.

Following the initial particle morphology assessment, we examined the crystalline structure of Class I, Class II, and bulk‐synthesized particles using powder X‐ray diffraction (PXRD) at room temperature (Figure S5a, Supporting Information). While both systems exhibit low crystallinity, comparing Class I and II particles with bulk [Fe(Htrz)_2_(trz)]BF_4_ (pure polymorph I) reveals notable differences. In bulk samples, there is an almost defined double peak and a well‐defined triple peak at 2θ ≈ 11° and 25°, respectively. In contrast, Class I particles display a single peak and a less distinct triple peak within the same 2θ range, suggesting that they crystallize as a mixture of polymorphs I and II, with polymorph II as the predominant phase. Conversely, Class II particles show a well‐defined double peak and triple peak in the 2θ range, though the peak ratio for the double peak differs significantly from that of the bulk sample. This observation indicates a mixture of polymorphs I and II, with polymorph I as the major component in Class II particles. The occurrence of mixed‐phase [Fe(Htrz)_2_(trz)]BF_4_ is not novel and has been observed to arise when the material is downscaled under conditions that differ significantly from the original synthetic methods used for bulk production,^[^ ^55^ ^]^ as reported by others.^[^ ^60^, ^61^, ^63^ ^]^

The magnetic properties of Class I and II particles were determined using temperature‐dependent magnetic susceptibility measurements (Table S3 and Figure S6, Supporting Information). Both Class I and II particles show slightly different magnetic behaviors in the χT versus T measurements (χ being the mass paramagnetic susceptibility) after the first heating/cooling cycle. While both Class I and II particles display abrupt hysteretic thermal transitions, their SCO switching temperature, T_1/2_, in the heating and cooling modes, are slightly shifted and the HS fraction at low temperature is higher for Class I particles. Moreover, the hysteresis width (ΔT) varies between the two classes, measuring 25 K for Class I and 29 K for Class II, compared to a ΔT of 40 K observed for bulk [Fe(Htrz)_2_(trz)]BF_4_ (Figure S7, Supporting Information). We associate all these differences in magnetism to the different mixtures of polymorphs found in the particles, as similar behaviors have been observed in other mixed phase [Fe(Htrz)_2_(trz)]BF_4_ compounds.^[^ ^61^ ^]^ Furthermore, the magnetic behavior of Class I and II particles, which is intermediate between that of [Fe(Htrz)_2_(trz)]BF_4_ polymorphs I and II,^[^ ^55^ ^]^ further supports our hypothesis regarding the formation of mixed‐phase compounds.

After assessing the physicochemical properties of Class I and II [Fe(Htrz)_2_(trz)]BF_4_ particles, we further explored the capabilities of the 3D coaxial flow‐focusing microfluidic device for the one‐pot production of SCO composite fibers, achieving in situ formation of both the SCO particles and the fiber matrix. We selected sodium alginate as a polymer matrix precursor due to its natural origin and capacity to polymerize into fibers upon interaction with cations.^[^ ^64^ ^]^ SCO composite fibers generation was achieved by flowing an aqueous solution of sodium alginate (0.75% w/v) and Htrz (2 m) with an ethanolic solution of Fe(BF_4_)_2_ (0.4 m) through the main reaction channel. As in the synthesis of Class I and II particles, the device configuration allows for the production of two distinct types of fibers—Class I and Class II fibers (Figure 1e; Figure S8, Supporting Information)—depending on the injection position of the SCO composite fiber precursors. Here, due to the nearly null diffusion coefficient of alginate (see Table S6, Supporting Information) and the distinct viscosities of the two precursor solutions, we expect the less viscous precursor solution (i.e., the ethanolic solution of Fe(BF_4_)_2_) to occupy a smaller cross‐section of the main microfluidic channel.

Numerical simulations of the flow and mass transport of these solutions in our device confirmed this hypothesis (see Simulation section S2, Supporting Information). Interestingly, the simulations revealed that for FRRs of 0.5, 4, and 9, the size of the focused stream depends on which precursor solution is injected through the central inlet 1, and thus, on whether Class I or Class II fibers are fabricated. Specifically, the simulation results show that for the same FRR, a more focused central stream is achieved when fabricating Class II fibers, as the less viscous ethanolic precursor solution is introduced through inlet 1 (Figure S31, Supporting Information). In sharp contrast, a less focused central stream occurs during the fabrication of Class I fibers, where the more viscous aqueous solution of sodium alginate and Htrz is introduced through inlet 1 (Figure S31, Supporting Information).

To elucidate how these changes in flow and mass transport during the 3D flow‐focusing of the precursor solutions influence the formation of SCO composite fibers and their resulting magnetic properties, we conducted a series of experiments varying the FRR (0.5, 4, and 9) while keeping the TFR constant at 750 µL min^−1^ for each fiber type (see Table S1, Supporting Information for further experimental details). When the ethanolic solution of Fe(BF_4_)_2_ was introduced through the side inlets and the aqueous alginate/Htrz solution through the central inlet, we obtained robust, solid fibers with a pink hue (Class I fibers). Conversely, injecting the Fe(BF_4_)_2_ solution through the central inlet and the alginate/Htrz solution through the side inlets produced darker composite fibers (Class II fibers). Further experimental details are provided in Table S1 (Supporting Information).

**Figure 2:**
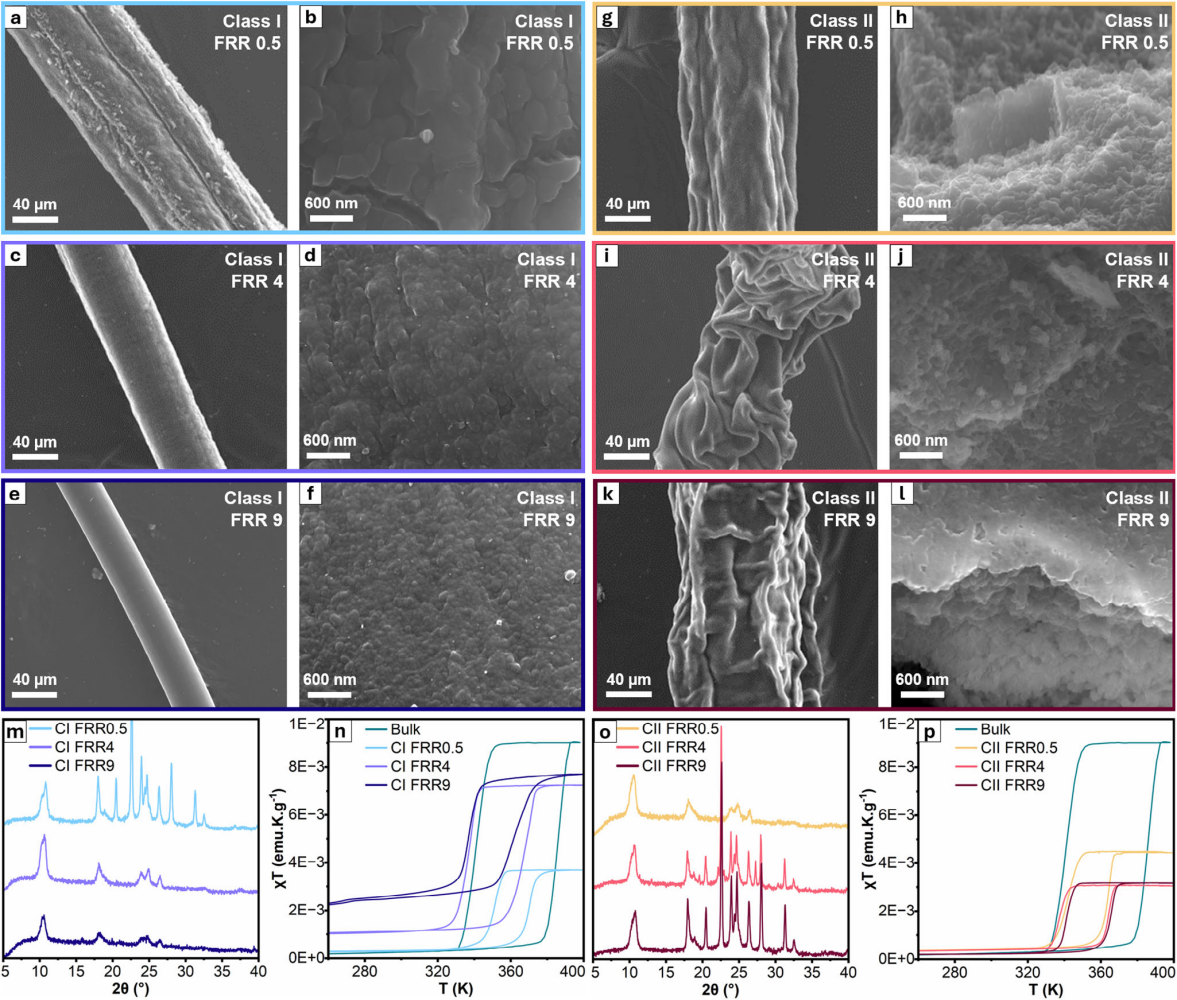
SEM images of all the SCO composite fibers synthesized with different conditions. Class I fibers—a,b) FRR 0.5, c,d) FRR 4, e,f) FRR 9. Class II fibers—g,h) FRR 0.5, i,j) FRR 4, k,l) FRR 9. Note: (b,d,f) are the SEM images focusing on the outer shell of Class I fibers, while (h,j,l) are the images focusing on the hollow core of Class II fibers. m,o) PXRD spectra of Class I and II fibers generated at different FRR, respectively. n,p) Thermal behavior of the magnetic susceptibility (χT) of Class I and II fibers compared with that of the bulk [Fe(Htrz)_2_(trz)]BF_4_, respectively.

To investigate the macro‐ and microscopic changes induced by varying precursor injection sites and FRRs, we examined the fibers using SEM. At low magnification, Class I and II fibers are readily distinguishable. Class I fibers exhibit a smooth surface that is slightly rougher than that of pure alginate fibers crosslinked with the ethanolic solution of Fe(BF_4_)_2_ (Figure S15, Supporting Information). Notably, the surface of Class 1 fibers becomes progressively uniform as FRR increases (**Figure** **2**a,c,e). In contrast, Class II fibers exhibit a wrinkled surface with folds that become more pronounced with increasing FRR (Figure 2g,i,k). The distinct morphologies observed between Class I and II fibers can be attributed to two key factors: the specific flow configurations and the selective diffusion behavior of Fe(II) ions, which readily diffuse into the alginate solution, while the alginate does not diffuse back into the Fe(II) ethanolic solution (see, Section S2 and Table S6, Supporting Information).

In Class I fibers (with Fe(BF_4_)_2_ in the sheath flows and alginate/Htrz in the central flow), fiber growth initiates as Fe(II) ions diffuse from the sheath flow into the central flow, forming a fiber that develops from the Fe(BF_4_)_2_‐alginate interface toward the center of the channel (Figures S32–S34, S38 and S40, Supporting Information). At a FRR of 0.5, numerical simulation results indicate that due to their lower diffusion coefficient, Fe(II) ions do not fully diffuse to the center of the main channel, likely resulting in hollow fiber formation (Figure S42a, Supporting Information). Increasing the FRR focuses the central flow, producing solid fibers with reduced diameters, as Fe(II) ions have to diffuse shorter distances to reach the center of the microfluidic channel and occupy its entire cross‐section (Figure S42b,c, Supporting Information).

In Class II fibers (with alginate/Htrz in the sheath flows and Fe(BF_4_)_2_ in the central flow), the diffusion pattern promotes outward fiber growth from the alginate‐Fe(BF_4_)_2_ interface (Figures S35–S37, S39 and S41, Supporting Information). Our simulation results suggest that this outward growth leads to hollow fiber formation at all FRRs (Figure S42d–f, Supporting Information). However, at higher FRRs, the flow focusing of the central flow results in thinner fiber walls, as Fe(II) ions have to diffuse longer distances to reach the main channel walls and are not able to do so over the whole length of the main channel. Upon viewing the morphology of the fibers in conjunction with the simulation results, it becomes evident that limited Fe(II) diffusion leads to highly wrinkled surfaces in Class II fibers prepared at FRRs of 4 and 9 due to their thinner walls, while Class I and II fibers prepared at a FRR of 0.5 exhibit less wrinkling and reduced folding, owing to their thicker walls formed in the widespread presence of Fe(II).

High‐magnification SEM images support these observations and provide valuable insights into SCO particle formation on the alginate matrix. It should be noted that the following analysis of particle size and shape will remain qualitative, as attempts to dissolve the fiber matrix to isolate the SCO particles consistently resulted in SCO particle dissolution, preventing a quantitative assessment.

In Class I fibers, high magnification images (Figure 2b,d,f) reveal particulates with undefined shapes homogeneously distributed on the fiber surface as well as in the core of the fiber (Figures S9 and S11, Supporting Information). However, our simulations predict that, due to the rapid outward diffusion of Htrz from the central to the sheath flow, SCO particles can also form outside the fiber matrix through reaction with Fe(BF_4_)_2_ (Figure S40, Supporting Information), which go into the waste solution after fiber isolation. Particle size on the fiber matrix progressively decreases with increasing FRR. This behavior is supported by numerical simulations, which reveal that the width of the RD zone at the end of the reaction channel decreases from ≈400 µm at a FRR of 0.5 to ≈250 µm at a FRR of 9 (Figures S42–S44, Supporting Information), due to flow focusing. Such decrease in the RD zone limits mixing, and particle growth at higher FRRs, a phenomenon that we also observed during the microfluidic synthesis of covalent organic frameworks and layered double hydroxides.^[^ ^65^, ^66^ ^]^

In Class II fibers, high magnification images confirm the formation of hollow fibers with SCO particles displaying undefined shapes (Figure 2h,j,l; Figures S10 and S12, Supporting Information). Notably, the SCO particles are homogeneously generated on the inner and within the walls of the alginate fiber, with no particles observed on the outer walls (Figures S10 and S12, Supporting Information). This distribution aligns with the flow configuration and the numerical simulation results, which predicted particle formation predominantly within the fiber matrix and on the inner surface of the hollow fiber (Figures S41–S43, Supporting Information). In contrast to Class I fibers, the size of the SCO nanoparticles within the polymer matrix in Class II fibers shows minimal variation between FRRs. The invariability in particle size can be attributed to several factors, including the limited presence of Fe(II) in the main channel as the FRR increases and the sustained width of the RD zone. At a FRR of 0.5, the Fe(II) injected into the main channel (1:2.5 Fe:Htrz) is slightly above the theoretical stoichiometric amount (1:3 Fe:Htrz) required to form [Fe(Htrz)_2_(trz)]BF_4_ (Table S1, Supporting Information). However, Fe(II) is also consumed by sodium alginate in the fiber formation process, likely leading to an iron concentration below the stoichiometric threshold. Additionally, while the diffusion coefficient of Fe(II) is relatively small (Table S6, Supporting Information), the flow configuration allows the outer diffusion of Fe(II) to reach the channel walls, resulting in a large RD zone (Figures S41g and S43d, Supporting Information). Therefore, at a FRR of 0.5 in Class II fibers, the consumption of Fe(II) in the large RD zone can likely bottleneck the [Fe(Htrz)_2_(trz)]BF_4_ formation and growth. At higher FRRs of 4 and 9, the insufficient Fe(II) injected in the main channel (1:20 and 1:45 Fe:Htrz) becomes the limiting factor, as [Fe(Htrz)_2_(trz)]BF_4_ cannot grow larger. Furthermore, the small diffusion coefficient of Fe(II) results in a similar width of the RD zone at these FRRs (Figures S42–S44, Supporting Information), consistent with the similarity in particle sizes. Consequently, in Class II fibers, the limited Fe(II) injection combined with the RD zone dimensions leads to particles of similar size across the different FRRs. These effects are not observed in Class I fibers due to the excess Fe(II) provided to the microfluidic device at higher FRRs of 4 and 9 (1:1.25 and 1:0.5 Fe:Htrz), allowing consistent formation of [Fe(Htrz)_2_(trz)]BF_4_. Here, however, the reduction of the RD zone becomes the limiting growth factor as explained above. At a low FRR of 0.5, although the amount of Fe(II) is very low (1:10 Fe:Htrz), the limited diffusion of Fe(II) plays in its favor by constraining the RD zone and leaving a significant amount of unreacted Htrz toward the center of the microfluidic channel (Figures S40g and S43a, Supporting Information), effectively balancing the reagent stoichiometry within the RD zone and enabling particle growth.

To complement the characterization, we assessed the chemical composition of pure alginate fibers crosslinked with the ethanolic solution of Fe(BF_4_)_2_ and Class I and II fibers using energy‐dispersive X‐ray spectroscopy (EDX). The spectra acquired is consistent with the expected composition of the SCO composite fibers and confirm the presence of C, O, Fe, and N, (Figures S13 and S14, Supporting Information). In contrast, N was not detected in the pure alginate fibers (Figure S15, Supporting Information).

We also conducted a thorough PXRD analysis (Figure 2m,o) to gain insight into the crystalline nature of Class I and II fibers. Both fiber classes, across all FRRs, exhibit low crystallinity and display broad peaks at lower 2θ ≈ 11°. This limited crystallinity along with the impurities from Htrz in some of the samples (see below) make it challenging to discern whether polymorph I or II of [Fe(Htrz)_2_(trz)]BF_4_ has been obtained.^[^ ^61^ ^]^ However, as with the synthesis of SCO particles, the definition and peak ratio of the double peak at 2θ ≈ 11° can be an acceptable indicator of the major presence of one of the two polymorphs. Thus, we conclude that the SCO composite fibers are a mixture of [Fe(Htrz)_2_(trz)]BF_4_ polymorphs I and II.^[^ ^61^ ^]^ Furthermore, even with the low crystallinity presented by Class I and II fibers across the different FRRs, some curious trends can be observed.

In Class I fibers, a progressive shift from a phase mixture containing more polymorph I to a different phase mixture containing more polymorph II can be seen as the FRR increases from 0.5 to 9 (Figure 2m). This is evidenced by the main double peak at 2θ ≈ 11°, which gradually evolves into a single broad peak, signaling a shift toward a phase mixture containing more polymorph II.^[^ ^61^ ^]^ In addition, as the FRR increases from 4 to 9, the triple peak at 2θ ≈ 25° diminishes and eventually forms a double peak, further indicating this polymorphic transition. Interestingly, this transition is observed exclusively in Class I fibers prepared at FRRs of 4 and 9—the only samples in which Fe(II) is present above the stoichiometric ratio relative to Htrz. This suggests that the Htrz shortage results in a [Fe(Htrz)_2_(trz)]BF_4_ phase mixture with an increased proportion of polymorph II, supporting our previous observation in the SCO particles. On the other hand, such evolution is not noticed in the main double peak at 2θ ≈ 11° for Class II fibers across all FRRs, suggesting that in all these fibers the excess of Htrz induces the formation of a phase mixture containing more polymorph I (Figure 2o).

To explain the Htrz impurities in the fibers at different FRRs, we analyzed each case individually. In Class I fiber prepared with a FRR of 0.5, the presence of crystalline Htrz is clear (Figure 2m; Figure S5b, Supporting Information). We attribute this impurity to the shortage of Fe(II) ions provided to the system coupled with their limited diffusion from the sheath to the central flow, as discussed in the morphological analysis of the fiber and supported by our simulations for Class I fibers (Figures S40 and S43, Supporting Information). This results in unreacted Htrz that crystallizes within the fiber. At higher FRRs of 4 and 9, the excess of Fe(II) ions supplied to the microfluidic device fully diffuse from the sheath to the central flow, enabling a complete reaction with Htrz and leading to a diffractogram free of Htrz impurities.

In contrast, Class II fibers show the opposite trend. PXRD patterns of Class II fibers obtained at higher FRRs of 4 and 9 indicate the presence of residual Htrz, whereas at a lower FRR of 0.5, the diffractogram shows pure [Fe(Htrz)_2_(trz)]BF_4_ (Figure 2o; Figure S5b, Supporting Information). This observation aligns with our simulation results and previous discussion (Figures S41 and S43, Supporting Information), though with an inverse rationale to that of Class I fibers. At a low FRR of 0.5, the Fe(II) ions supplied to the microfluidic device diffuse completely across the sheath flow, occupying the entire cross‐section of the device and allowing full reaction with Htrz. Conversely, at FRRs of 4 and 9, the insufficient availability of Fe(II) ions results in unreacted Htrz within the fibers.

Following the basic physicochemical characterization, we examined the magnetic properties of the SCO composite fibers by means of temperature‐dependent magnetic susceptibility measurements (Figure 2n,p). Before beginning the discussion, it is important to clarify that the magnetic properties presented here correspond to the second heating‐cooling cycle, as Class I and II fibers suffer from a shrinking of the magnetic hysteresis after the first cycle. This shrinkage is not new in this family of [Fe(Htrz)_2_(trz)]BF_4_ compounds and has been observed in the polymorph II of bulk [Fe(Htrz)_2_(trz)]BF_4_ and [Fe(Htrz)_2_(trz)]BF_4_ nanoparticles obtained using micelles as confined reactors.^[^ ^53^, ^55^, ^67^ ^]^ In general, such changes have been associated with an irreversible transition from a metastable to a stable crystalline phase upon heating the sample.^[^ ^68^, ^69^, ^70^ ^]^ For the first to third magnetic thermal cycles, we refer the reader to Figure S16 (Supporting Information).

The *χT* versus *T* curves (χ being the mass paramagnetic susceptibility) for Class I and II fibers exhibit an abrupt hysteretic transition, similar to the behavior displayed by Class I and II particles (Figure 2n,p; Figure S17 and Table S4, Supporting Information). The hysteresis loop is significantly reduced in comparison to bulk [Fe(Htrz)_2_(trz)]BF_4_ polymorph I (Figure S7, Supporting Information) for both fiber classes synthesized at three different FRRs. This reduction in cooperativity can likely be attributed again to the mixture of [Fe(Htrz)_2_(trz)]BF_4_ polymorphs I and II present in our SCO composite fibers, as has been shown before in mixed phase [Fe(Htrz)_2_(trz)]BF_4_ nanoparticles prepared using micellar approaches.^[^ ^61^ ^]^ Nevertheless, the possibility that the alginate matrix induces lattice strain in the SCO compound, thereby additionally contributing to the observed differences in magnetic behavior, cannot be ruled out.

Notably, for Class I fibers, a gradual increase in the Fe(II) HS fraction at low temperatures is observed as the FRR rises from 0.5 to 4 and 9, accompanied by an overall increase in the magnetic signal during thermal cycling. Although HS stabilization could, in principle, be attributed to nanoparticle size reduction^[^ ^67^ ^]^—where surface Fe atoms with incomplete coordination spheres tend toward the HS state—such effects typically occur only in extremely small SCO particles, specifically with sizes ranging from 6 to 10 nm.^[^ ^21^, ^67^, ^71^ ^]^ Therefore, the HS stabilization in Class I fibers likely stems from an alternative mechanism.

We hypothesize that the HS stabilization is driven by variations in the polymorph I and II ratios of [Fe(Htrz)_2_(trz)]BF_4_, as it has been observed previously.^[^ ^61^ ^]^ According to our crystallographic analysis, all systems crystallize as mixtures of polymorphs I and II. However, in Class I fibers, a progressive shift from a phase mixture containing more polymorph I to a different phase mixture containing more polymorph II can be seen as the FRR increases from 0.5 to 9 (Figure 2m). Interestingly, this control over the mixture of polymorphs and stabilization of the HS fraction was previously achieved by tuning the concentration of Fe(II) in the reaction media while keeping a stoichiometric ratio with Htrz.^[^ ^61^ ^]^ In our case, this compositional and magnetic tuning is achieved on‐the‐fly by precisely controlling the RD zone, specifically the Fe(II):Htrz ratio, during the reaction process.

Since the magnetic measurements are reported per gram of fiber rather than per quantity of SCO particles alone—and given that all [Fe(Htrz)_2_(trz)]BF_4_ phase mixtures display similar abrupt and almost complete spin transitions^[^ ^61^ ^]^—the increase in magnetic signal likely reflects a higher number of SCO particles formed as the FRR increases in Class I fibers. As discussed in the morphological characterization, at a FRR of 0.5, the limited amount of Fe(II) injected into the main microfluidic channel, dispersed across a relatively broad RD zone, produces larger particles. However, the limited diffusion of Fe(II) becomes a bottleneck in the formation of more [Fe(Htrz)_2_(trz)]BF_4_ particles. In contrast, at FRRs of 4 and 9, the increased Fe(II) input and its complete diffusion into Htrz (and vice versa) result in the formation of more SCO particles, correlating with the rise in magnetic signal (Table S1 and Figure S43a–c, Supporting Information). The slight decrease in the magnetic signal observed between FRR 4 and 9 is likely attributed to the narrowing of the RD zone. This reduction in width constrains the space available for the growth and formation of [Fe(Htrz)_2_(trz)]BF_4_ particles, resulting in a lower number of particles being formed (Figure S44, Supporting Information).

Furthermore, the magnetic signals observed for Class II fibers differ significantly from those of Class I. The HS fraction remains stable across varying FRRs, which can be explained with prior arguments. Specifically, in Class II fibers, phase mixtures across different FRRs are similar, with no crystallographic evolution detected, resulting in a consistent HS fraction (Figure 2o). This finding suggests that the crystallographic evolution across FRRs in Class I fibers is key to HS state stabilization. Additionally, it supports our hypothesis that a shortage of Htrz in Class I fibers drives their crystallographic evolution toward a phase mixture with a higher proportion of polymorph II, whereas the excess ligand in Class II fibers prevents this evolution, maintaining a constant HS fraction.

As for the magnetic signal, at FRR 0.5, the Fe(II) quantity injected in the microfluidic device during the formation of Class II fibers is near the minimum threshold necessary for the formation and growth of [Fe(Htrz)_2_(trz)]BF_4_₄ particles. This leads to a greater number of particles than at FRRs of 4 and 9, where a substantial shortage of Fe(II) results in fewer particles and, consequently, a lower magnetic signal (Table S1 and Figure S43d–f, Supporting Information).

We also investigated the effects of increasing or reducing the TFR during the production of Class I fibers. However, these adjustments either inhibited the formation of SCO particles within the fibers or resulted in the clogging of the device (Table S5, Supporting Information). Given the lack of success in these trials, we chose not to pursue the synthesis of Class II fibers under similar conditions, as meaningful comparisons between the two classes would not be feasible.

Overall, we can conclude that the 3D coaxial flow‐focusing microfluidic device used here is a powerful tool to manipulate the Fe(II):Htrz ratio on‐the‐fly. This is achieved by precisely tuning the RD zone at will, simply by altering the injection position of the precursor solutions and their flow rates. This control is not only useful for the continuous preparation of SCO composite fibers with tailored macro‐ and microscale features but also for generating materials with marked differences in their magnetic properties.

**Figure 3:**
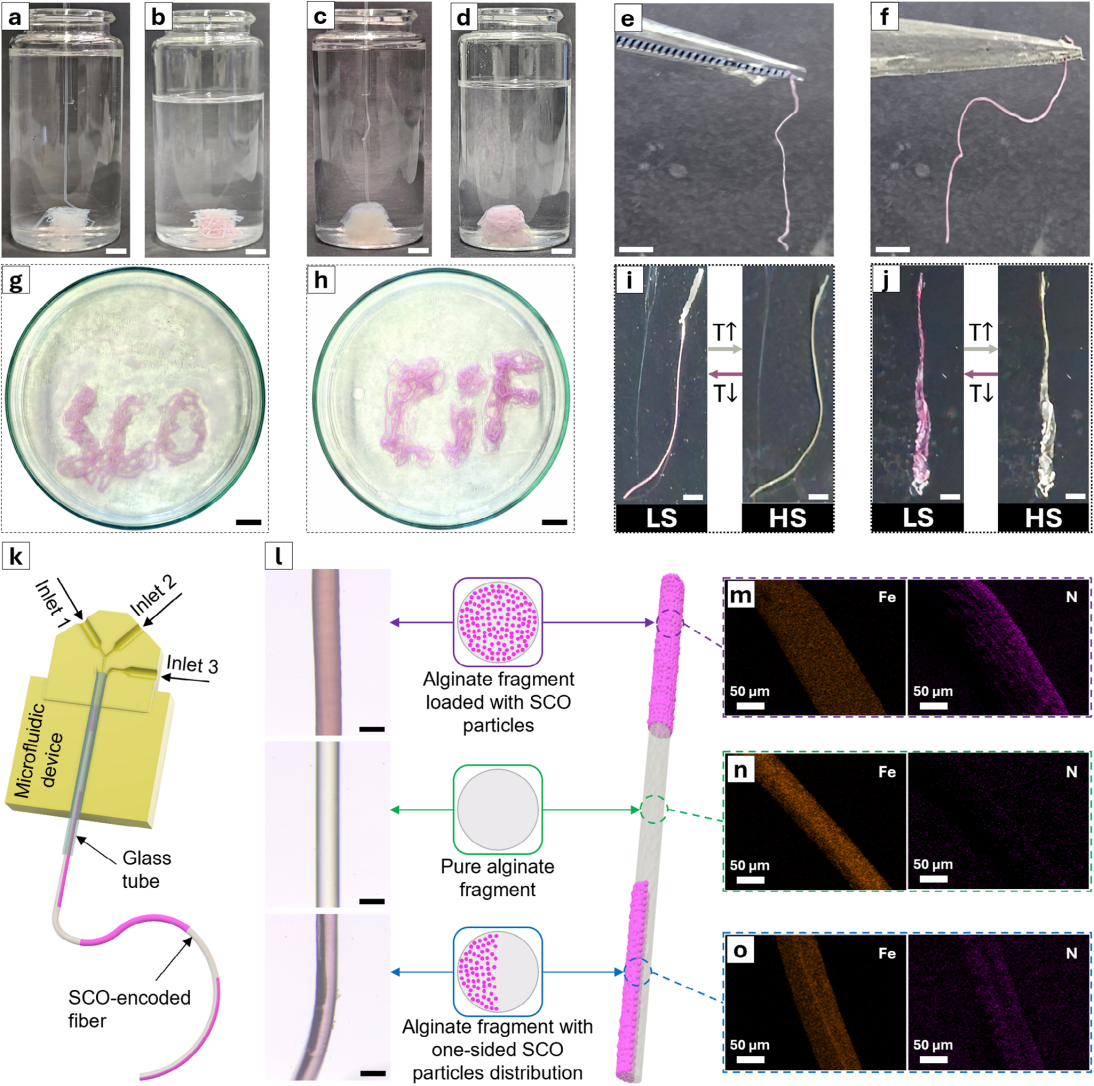
Continuous formation of SCO composite fibers and their transition to pink color after 5 min—a,b) Class I fiber, c,d) Class II fiber—scale bar: 5 mm. e,f) Optical image of self‐standing class I fiber and class II fiber, respectively—scale bar: 5 mm. g) Class I fiber and h) Class II fiber drawn on Petri dishes—scale bar: 10 mm. Thermal treatment by heating of i) Class I fiber and j) Class II fiber up to 100 °C (right) and cooling down to 20 °C (left)—scale bar: 2 mm. k) 3D model showing the microfluidic device used to produce SCO‐encoded fiber. l) Micrographs and schematic representations of the SCO‐encoded fiber with fragments fully or partially loaded with SCO particles separated by a pure alginate fragment—scale bar: 150 µm. m) EDX images of the alginate fragment fully loaded with SCO particles. n) EDX images of the pure alginate fiber fragment. o) EDX images of the alginate fragment with one‐sided SCO particles distribution.

After demonstrating the precise control over the RD zone that the coaxial 3D flow‐focusing microfluidic device provides, we explored its versatility in continuously printing Class I and II fibers. As shown in **Figure** **3**a–h, Class I, and II fibers can be printed to construct different architectures, including 3D structures and intricate patterns (Figure 3a–d,g,h, respectively), or even to produce freestanding isolated fibers (Figure 3e,f). The resulting architectures exhibit excellent long‐term stability in air, although they show limited stability under harsh conditions after 24 h (Figure S18, Supporting Information, and related discussion).

Nanoindentation measurements reveal that the fibers exhibit elastic behavior with Young's modulus values in the MPa range (Figure S19, Supporting Information), which is consistent with those reported for alginate‐based hydrogels and similar soft materials.^[^ ^72^, ^73^, ^74^, ^75^ ^]^ Regardless of the printing configuration, the fibers clearly exhibit a color change from pink to white upon heating to 100 °C, indicating the LS to HS transition in [Fe(Htrz)_2_(trz)]BF_4_ (Figure 3i,j). This effect is reversible, with the pink color returning when the fiber is cooled back to room temperature. Beyond this visual change, temperature‐dependent PXRD analyses further confirm the SCO behavior in both fiber types (Figure S20, Supporting Information).

Together, these results demonstrate our ability to continuously print thermochromic fibers, offering a robust and scalable strategy to overcome longstanding challenges in the processing of SCO materials. However, note that in Class I and Class II fibers, the SCO material can only be distributed radially within the alginate fiber. In contrast, nature provides remarkable examples of compositional control in freestanding structures. For example, spiders can modulate the chemical composition of proteins within their silk fibers sequentially and non‐radially across the fiber's diameter, tailoring the silk precisely to its intended function.^[^ ^76^, ^77^ ^]^ Inspired by this natural phenomenon, we redesigned the microfluidic device to achieve temporal control over the spatial distribution of [Fe(Htrz)_2_(trz)]BF_4_ within the fibers to generate SCO‐encoded fibers.

As shown in Figure 3k, the redesigned microfluidic device features a central channel that is fed by two inlets, while one of the original side channels has been removed, leaving a single side inlet (Figure 3k; Figure S1b, Supporting Information). Using this configuration, customized fibers (Figure 3l) were fabricated by injecting a 0.75% (w/v) sodium alginate solution with 2 m Htrz through central inlet 1, a pure 0.75% (w/v) sodium alginate solution through central inlet 2, and a 0.4 m ethanolic solution of Fe(BF₄)₂ through sheath flow inlet 3 (see the Supporting Information for further details). By selectively activating the central inlets while keeping the reagent‐laden flow of inlet 3 consistently active throughout fiber fabrication, we successfully produced continuous SCO‐encoded fibers on‐the‐fly.

When only central inlet 1 is activated alongside the reagent‐laden flow of inlet 3, we obtain alginate fragments fully loaded with SCO particles, closely resembling Class I fibers, as confirmed by SEM and EDX analysis (Figure 3m). Activating only central inlet 2 together with the reagent‐laden flow of inlet 3 produces pure alginate fragments, confirmed by the absence of nitrogen throughout the fiber (Figure 3n). Finally, simultaneously activating all the inlets yields alginate fiber fragments with SCO particles distributed non‐radially across the fiber's diameter, i.e., only on one side (Figure 3o), as evidenced by the distinct “two‐faced” structure observed in SEM images and the EDX analysis, which shows differing Fe intensity across the central symmetry line.

To our knowledge, this represents the first demonstration of spatiotemporal control over the chemical composition of an SCO composite material achieved in a single step, through a continuous process, and with remarkably short preparation times (within seconds; see Supporting Information). This chemical encoding of molecular switches unlocks exciting new possibilities for their application in real‐world devices. For instance, by enabling the precise printing of these SCO composites and SCO‐encoded fibers into defined shapes and architectures, we pave the way for their integration into devices, expanding the possibilities for application‐specific customization and facilitating their real‐world implementation.

## 3 Conclusion

We have demonstrated that our continuous flow microfluidic devices operating under 3D control of the RD zone can enable the direct and continuous fabrication of SCO polymeric fibers with a homogeneous distribution of SCO material within the polymer matrix. The resulting SCO composite fibers exhibit both magnetic bistability and thermochromic properties upon heating and cooling. By leveraging the precise control over the RD zone offered by the microfluidic chip design—through adjustment of precursor injection positions and flow rates—we can tailor SCO composite fibers with hollow or solid structures that display a variety of magnetic behaviors. Moreover, this technique also facilitates the printing of freestanding architectures with defined shapes and the isolation of fibers as self‐standing units. Building on these capabilities, we engineered a new chip design that provides precise encoding of SCO composite fibers by leveraging spatiotemporal control over the distribution of the SCO material within them, thereby significantly expanding their practical applicability. Our findings highlight the transformative potential of this microfluidic approach, opening novel pathways for scientific and industrial communities to incorporate molecular switches into materials suited for real‐world applications.

## Conflict of Interest

The authors declare no conflict of interest.

## Author Contributions

A.T.N. and D.A. contributed equally to this work. The manuscript was written and revised through the contributions of all authors. All authors have given approval to the final version of the manuscript.

## Supporting information



Supporting Information

## Data Availability

The data that support the findings of this study are available from the corresponding author upon reasonable request.
