# Supplementary material for: On‐the‐Fly Synthesis of Freestanding Spin‐Crossover Architectures With Tunable Magnetic Properties
Source: Adv Mater. 2025 Jun 13;37(37):2420492. doi: 10.1002/adma.202420492 (PMC12447033; doi:10.1002/adma.202420492)
Supplement: Supplementary file 1 — Supporting Information [file ADMA-37-2420492-s001.docx]

Supporting Information

**On-the-Fly Synthesis of Freestanding Spin-Crossover Architectures with Tunable Magnetic Properties**

Anh Tuan Ngo†, David Aguilà†, João Pedro Vale, Semih Sevim, Michele Mattera, Jordi Díaz-Marcos, Ramón Pons, Guillem Aromí, Bumjin Jang, Salvador Pané Vidal, Tiago Sotto Mayor*, Mario Palacios-Corella*, Josep Puigmartí-Luis*

**Table of Contents**

[**S1. Experimental Section** S3](#_Toc183188769)

**[S2. Simulation Section](#_S2._Simulation_Section)** [S21](#_S2._Simulation_Section)

[***Fluid properties*** S21](#_Toc183188771)

[***Geometry and boundary conditions*** S22](#_Toc183188772)

[***Numerical methods*** S23](#_Toc183188773)

[***Mesh independence testing and validation*** S23](#_Toc183188774)

[***Results*** S25](#_Toc183188775)

[**References** S41](#_Toc183188776)

# S1. Experimental Section

***Co-axial flow-focusing device***: All the microfluidic devices were designed using the Autodesk Fusion 360 software. The fabrication of these devices was performed with a commercial DLP 3D printer (Profluidics 285D, CADworks3D, Canada) using a transparent resin (Clear Microfluidics Resin V7.0a, CADworks3D, Canada).

The microfluidic devices contain two parts. The top parts include three inlets and one outlet for press-fitting a glass tube with an inner diameter of 1 mm and a length of 6 mm (**Figure S1**). This glass tube acts as the main reaction channel of the devices. The bottom part serves as the support to keep the glass tube straight and centered.

***Samples preparation***: All chemicals were purchased from Sigma-Aldrich Co. and used as received. Several different reaction conditions were screened in order to obtain the required solution concentration and flow rates for generating class I and class II fibers in the three-channel device. Faster flow rates and lower concentrations did not allow SCO particles to be produced on the fiber, while slower flow rates and higher concentrations prompted clogging on the device.

*Microfluidic synthesis of* *class I particles*: An aqueous solution (3 mL) containing 1,2,4-triazole (HTrz) (432 mg, 6.0 mmol) was prepared and connected to the central channels of the co-axial flow-focusing device (**Figure S1a**) using a 3 mL Fisher plastic syringe. A second solution was prepared by dissolving Fe(BF_4_)_2_·6H_2_O (810 mg, 2.4 mmol) in 6 mL of ethanol, which was filtered using a Sartorius Minisart NY Syringe filter to ensure clear solution and connected to the lateral channels using 3 mL Fisher plastic syringes. The insertion of the solutions for each inlet was controlled by using a neMESYS syringe pump (Cetoni GmbH, Germany) ensuring a 150 μL/min and 300 μL/min flow rates for the central and lateral inlets, respectively. The generated purple material was collected from the outlet directly on a vial immersed in an ethanol solution and was continuously produced for three minutes. The formed suspension was filtered, and the resulting purple solid was washed with ethanol and dried in air overnight.

*Microfluidic synthesis of* *class II particles:* The synthesis of class II particles was carried out following the same procedure as class I particles, inserting in this case the ethanolic Fe(BF_4_)_2_ solution in the central inlet channel and the aqueous 1,2,4-triazole solution in the two lateral inlet channels. In this case, 150 μL/min and 300 μL/min flow rates were used for the central and lateral inlets, respectively.

*Class I fibers*: An aqueous solution (10 mL) containing sodium alginate (75 mg) and 1,2,4-triazole (1.44 g, 20 mmol) was prepared and connected to the central channel of the co-axial flow-focusing device (**Figure S1a**) using a 10 mL Fisher plastic syringe. A second solution was prepared by dissolving Fe(BF_4_)_2_·6H_2_O (1.35 g, 4 mmol) in 10 mL of ethanol, which was filtered using a Sartorius Minisart NY Syringe filter to ensure clear solution, and used for both lateral channels with 5 mL Fisher plastic syringes. The insertion of the solutions for each inlet was controlled by using a neMESYS syringe pump (Cetoni GmbH, Germany) following the flow rates configurations as shown in **Table S1**. The generated fiber was collected from the outlet directly on a vial immersed in an ethanol solution and was continuously produced for three minutes. After removing the solvent, the hybrid material was washed with ethanol to remove unreacted species and dried in air overnight.

*Class II fibers*: The synthesis of class II fiber was carried out following the same procedure as class I fiber, inserting in this case the ethanolic Fe(BF_4_)_2_ solution in the central inlet channel and the aqueous sodium alginate / 1,2,4-triazole solution in the two lateral inlet channels. The flow rate conditions of each experiment for Class II fibers are shown in **Table S1**.

*Alginate fiber*: The synthesis of alginate fibers was carried out following the same procedure as class I, without the addition of 1,2,4-triazole in the aqueous solution. After removing the ethanol solution, the generated white fibers were dried in air overnight.

*Customized composite SCO fiber*: To fabricate this type of fiber, the microfluidic device shown in **Figure S1b** was used. Typically, an aqueous solution (3 mL) containing sodium alginate (22.5 mg) and 1,2,4-triazole (432 mg, 6 mmol) was prepared and connected to the central input 1. The other central input was connected to a 3 mL solution containing 22.5 mg sodium alginate. The solution connected to sheath flow input was prepared by dissolving Fe(BF4)2·6H2O (1.35 g, 4 mmol) in 10 mL of ethanol and then filtered using a Sartorius Minisart NY Syringe filter. The insertion of the solutions for each inlet was controlled by using a neMESYS syringe pump (Cetoni GmbH, Germany) ensuring a 600 μL/min and 75 μL/min flow rates for the lateral inlet and central inlets, respectively. By switching on/off the flow of the central inlets, we could generate a continuous fiber with fragments of different materials shown in **Figure 3** of the main text. For instance, when we injected into both central inputs simultaneously, we obtained alginate fiber fragments with a one-sided distribution of SCO particles. Conversely, when we halted the flow from central input 1 and continued with the other, we produced a pure alginate fragment. Finally, when we stopped central input 2 and injected through central input 1, the resulting alginate fragment was fully loaded with SCO particles.

*Bulk synthesis of [Fe(Htrz)_2_(trz)]BF_4_ particles*. Bulk SCO compound was prepared following the reported procedure.^[1,2]^ A solution of 1,2,4-triazole (1.040 g, 15 mmol) in ethanol (5 mL) was added to a solution of Fe(BF_4_)_2_·6H_2_O (1.687 g, 5 mmol) in water (10 mL). The obtained purple suspension was left undisturbed for 24 h and filtered. The resulting purple powder was washed with ethanol and dried in air overnight.

***Scanning electron microscopy (SEM)***: All samples were deposited on conductive carbon adhesive tape sputtered with a graphite layer and characterized using a JEOL JSM-7001F SEM with secondary and retrodispersed electron detectors.

***Energy-dispersive X-ray spectroscopy (SEM-EDX)***: Class I, class II and pure alginate fibers were analyzed using the JEOL J-7100FE SEM with an EDS and backscattered electrons detector. The samples were deposited on a conductive carbon adhesive tape and sputtered with graphite layer prior measurements.

***Magnetic measurements***: Magnetic susceptibility measurements were performed with a Quantum Design MPMS5 SQUID magnetometer at the “Unitat de mesures Magnètiques” of the Universitat de Barcelona. Diamagnetic corrections for the sample holder were applied. All thermal cycles were performed using 2 K/min for both heating and cooling modes.

***Powder X-Ray Diffraction***: Samples were gently grinded and deposited on a silicon single crystal substrate. Measurements were carried out using a Malvern Panalytical AERIS diffractometer equipped with a PIXcel detector and a Cu anode to select Cu Kα wavelength***.*** For the temperature-variable XRD experiments, an Anton Paar XRDynamic 500 multipurpose powder diffractometer equipped with an Anton Paar CHC plus+ Cryo and Humidity Chamber in the heating mode was used. The samples were measured in a bragg-Brentano divergent beam configuration, using a Ni/C multilayer monochromator with a 360 mm of radius. In addition, for these experiments, a Cu (λ_Kα_=1.5418 Å) radiation source was used and the samples were measured in powder with voltage-current set to 40 kV-50mA.

***Mechanical properties measurement:*** Fiber samples were deposited on glass slides. Nanoindentation measurements of these samples were performed using a Bruker AFM8 multimode atomic force microscope equipped with Nanoscope V electronics. The experiments were conducted in PeakForce Quantitative Nanomechanics (QNM) mode. A silicon tip on a nitride lever (SNL-10, Bruker) with a nominal spring constant (k) of 0.2891 N/m and a tip radius of approximately 40 nm was utilized. The cantilever had a thickness of 600 nm and a resonant frequency in the range of 40–75 kHz.

**
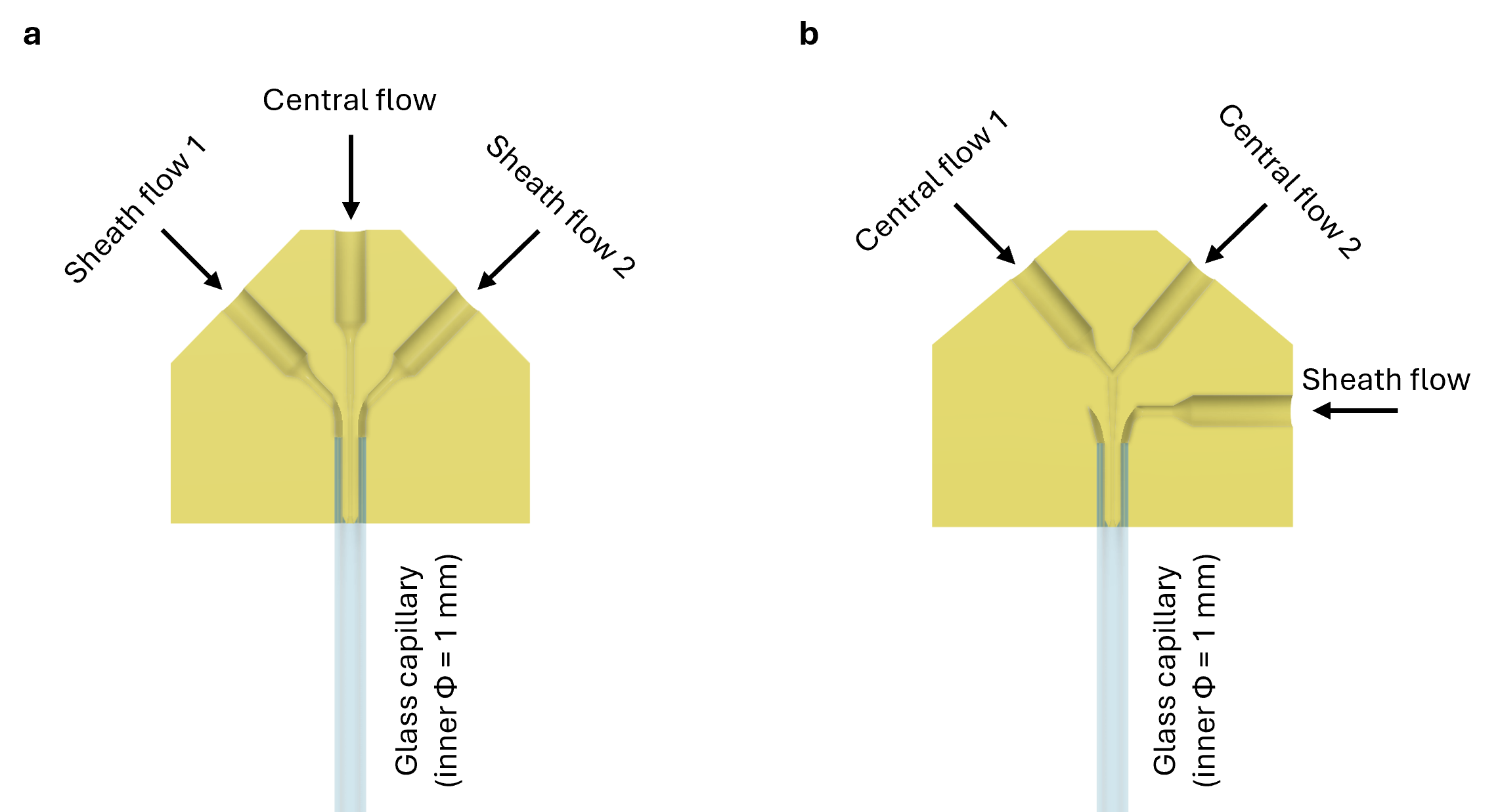
**

**Figure S1.** Schematic drawing showing the top-part of microfluidic devices used in this study. (a) Device used to synthesize particles, as well as Class I and Class II fibers. (b) Device used to synthesize customized composite SCO fiber.

**Table S1**. Experimental conditions to generate Class I and Class II particles and fibers

| **Experiments** | **Flow rate ratio** | **Total flow rate (µL/min)** | **Flow configuration** | |
| --- | --- | --- | --- | --- |
|  |  |  | ***Center flow*** | ***Sheath flow*** |
| Class I particles | **4**  (300 µL/min : 150 µL/min : 300 µL/min) | 750 | Htrz | Fe(BF_4_)_2_ |
| Class II particles | **4**  (300 µL/min : 150 µL/min : 300 µL/min) |  | Fe(BF_4_)_2_ | Htrz |
| Class I fibers | **0.5**  (125 µL/min : 500 µL/min : 125 µL/min) |  | Sodium Alginate + Htrz | Fe(BF_4_)_2_ |
|  | **4**  (300 µL/min : 150 µL/min : 300 µL/min) |  |  |  |
|  | **9**  (337.5 µL/min : 75 µL/min : 337.5 µL/min) |  |  |  |
| Class II fibers | **0.5**  (125 µL/min : 500 µL/min : 125 µL/min) |  | Fe(BF_4_)_2_ | Sodium Alginate + Htrz |
|  | **4**  (300 µL/min : 150 µL/min : 300 µL/min) |  |  |  |
|  | **9**  (337.5 µL/min : 75 µL/min : 337.5 µL/min) |  |  |  |

**
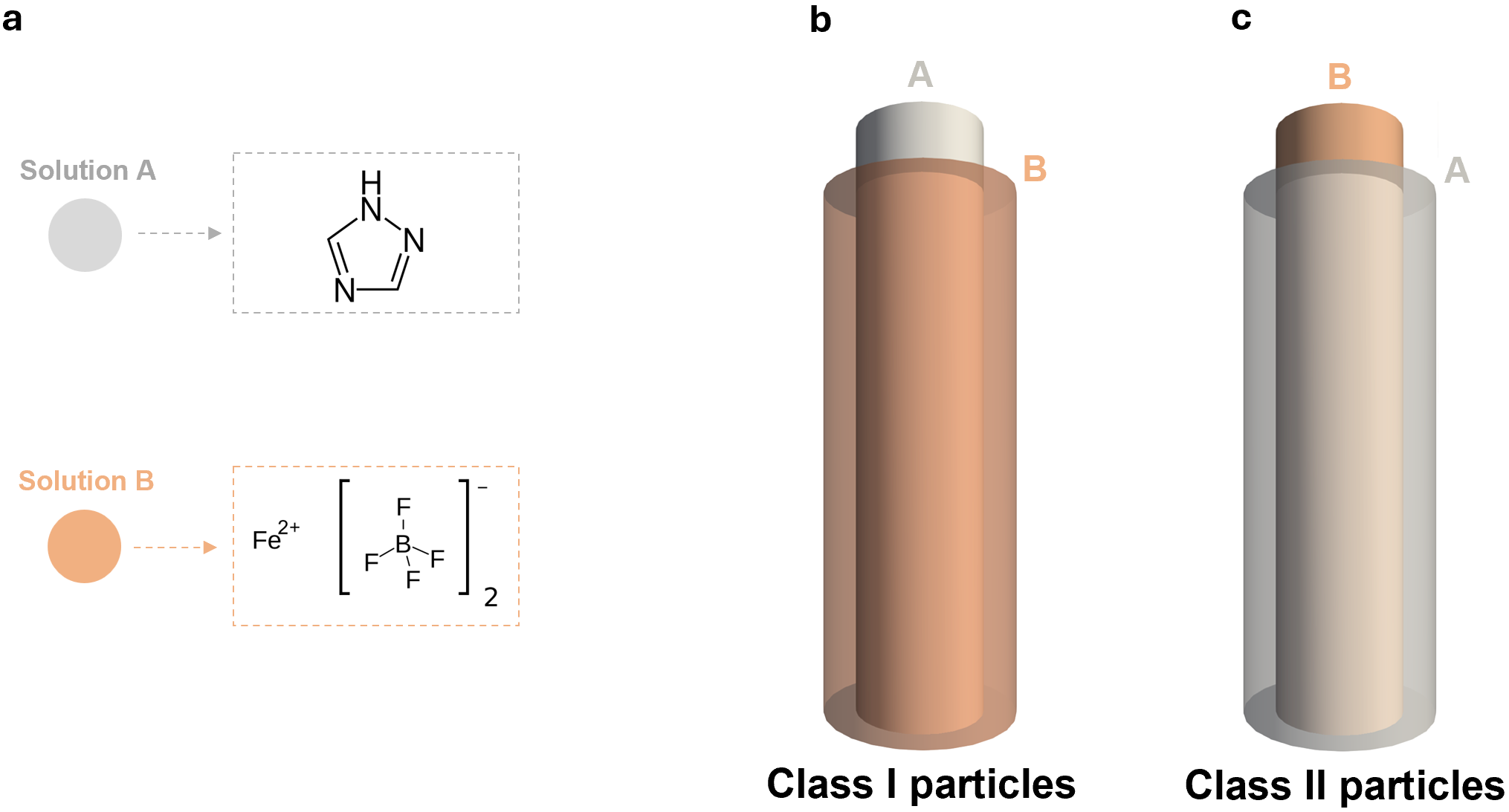
**

**Figure S2**. (a) Two solutions used to generate microfluidic SCO particles. Solution A is a aqueous solution of 1,2,4-triazole, while solution B is a ethanolic solution of Fe(II) tetrafluoroborate. (b) Flow configuration to generate Class I particles: solution A is flown through the middle inlet, while solution B is flown through the side inlets. (c) Flow configuration to generate Class II particles: solution B is flown through the central inlet, while solution A is flown through the lateral ones.

**Table S2**. Class I and II particle production rate (mg/min) at different FRRs. FRR of 0.5 has been excluded due to the clogging of the device.

| **Flow rate ratio** | |  |
| --- | --- | --- |
| **Experiments** | **4** | **9** |
|  |  |  |
| Class I particles (mg/min) | 5.5 | 0.6 |
| Class II particles (mg/min) | 4.7 | 1.1 |

**
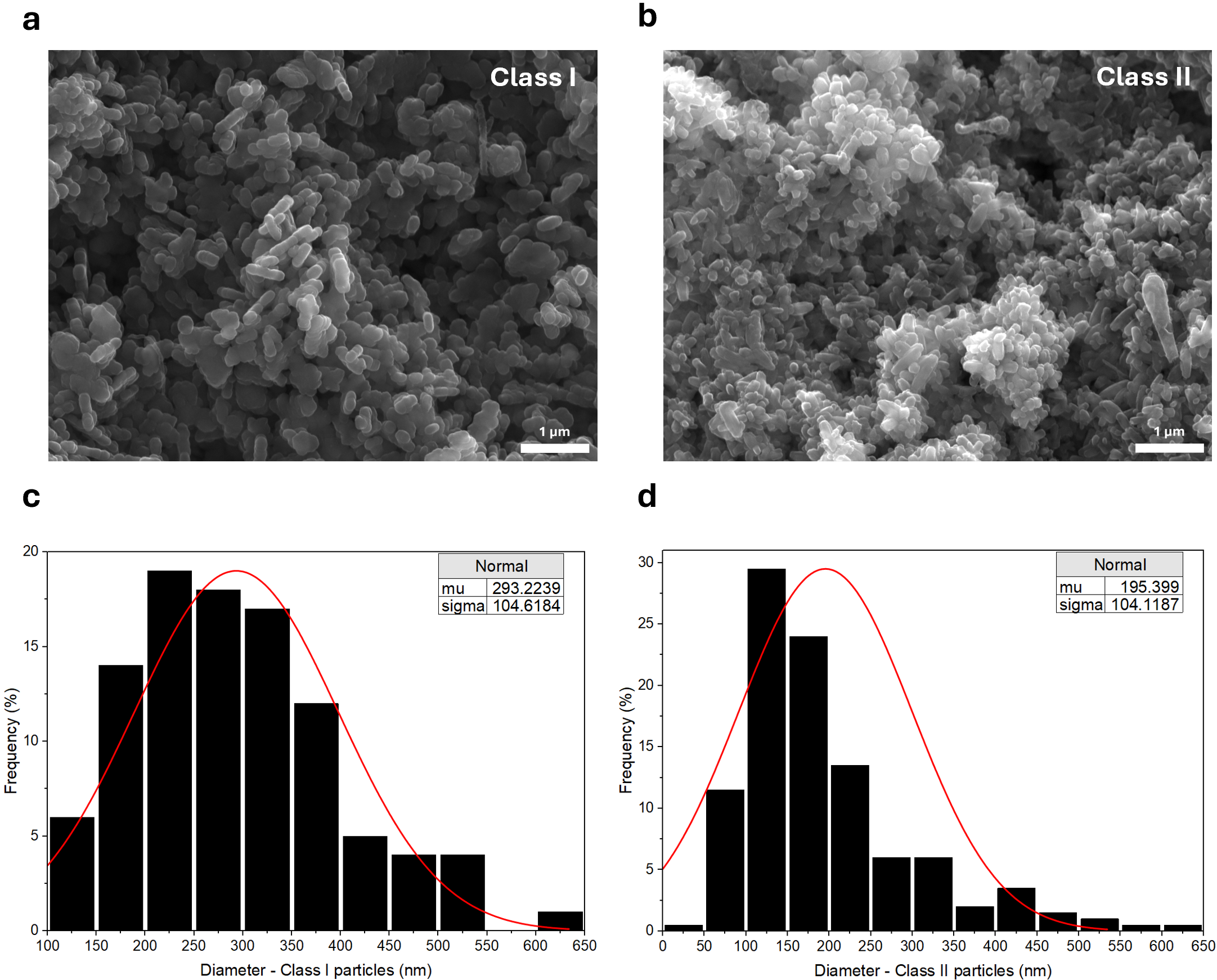
**

**Figure S3.** SEM images of (a) Class I and (b) Class II particles obtained using the 3D coaxial flow focusing microfluidic device. Size distribution histogram estimated from 100 particles of (c) Class I and (d) Class II particles from SEM analysis.


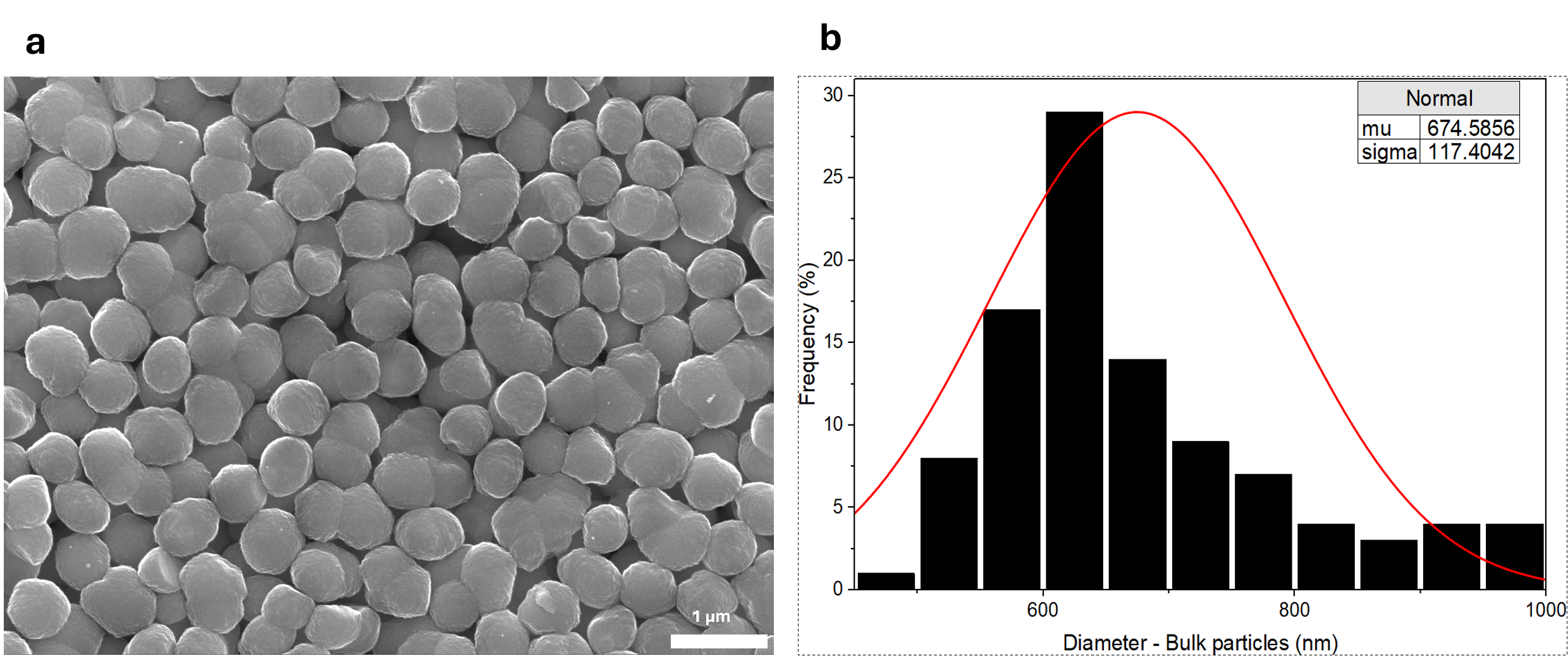


**Figure S4**. (a) SEM images of bulk [Fe(HTrz)_2_(trz)]BF_4_ particles and (b) their size distribution estimated from 100 particles.


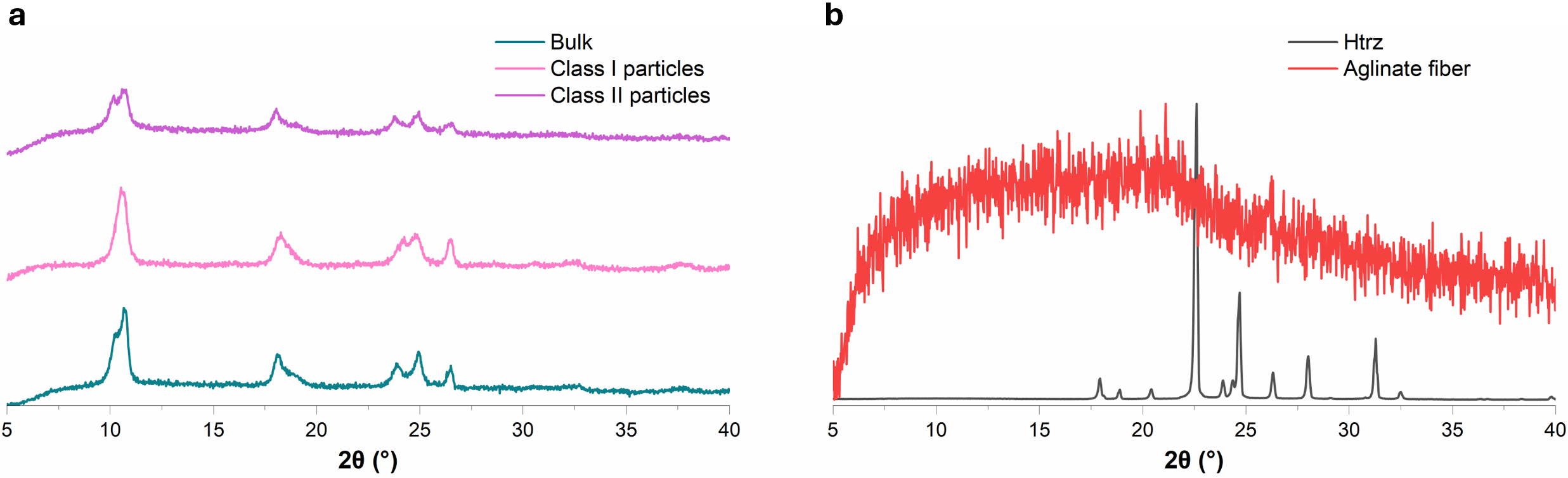


**Figure S5**. (a) PXRD spectra of Bulk SCO particles, Class I particles, Class II particles. (b) PXRD spectra of 1,2,4-triazole before reaction, and cross-linked pure alginate fiber.

**
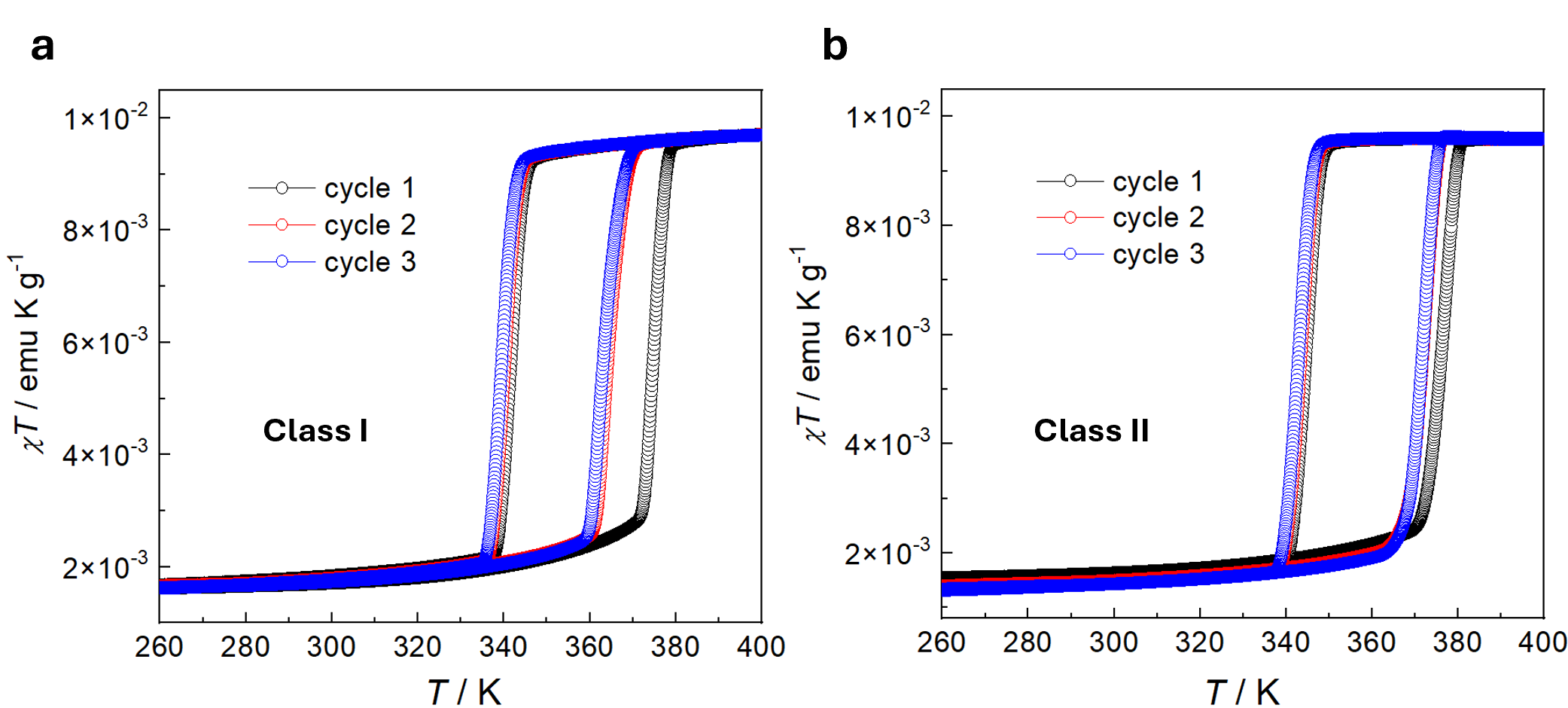
**

**Figure S6**. Thermal behavior of the magnetic susceptibility (*χT*) for [Fe(HTrz)_2_(trz)]BF_4_ particles generated using the 3D microfluidic device collected on three consecutive cycles. (a) Class I particles. (b) Class II particles.

**Table S3**. Transition temperatures observed for the SCO particles during the first and second thermal cycles obtained from magnetic measurements.

|  | **First thermal cycle** | | | **Second thermal cycle** | | |
| --- | --- | --- | --- | --- | --- | --- |
|  | ***T*_c_↑ (K)** | ***T*_c_↓ (K)** | **Δ*T* (K)** | ***T*_c_↑ (K)** | ***T*_c_↓ (K)** | **Δ*T* (K)** |
| **Bulk particles** | 389 | 343 | 46 | 387 | 341 | 46 |
| **Class I particles** | 375 | 343 | 32 | 364 | 340 | 24 |
| **Class II particles** | 377 | 345 | 32 | 372 | 343 | 29 |


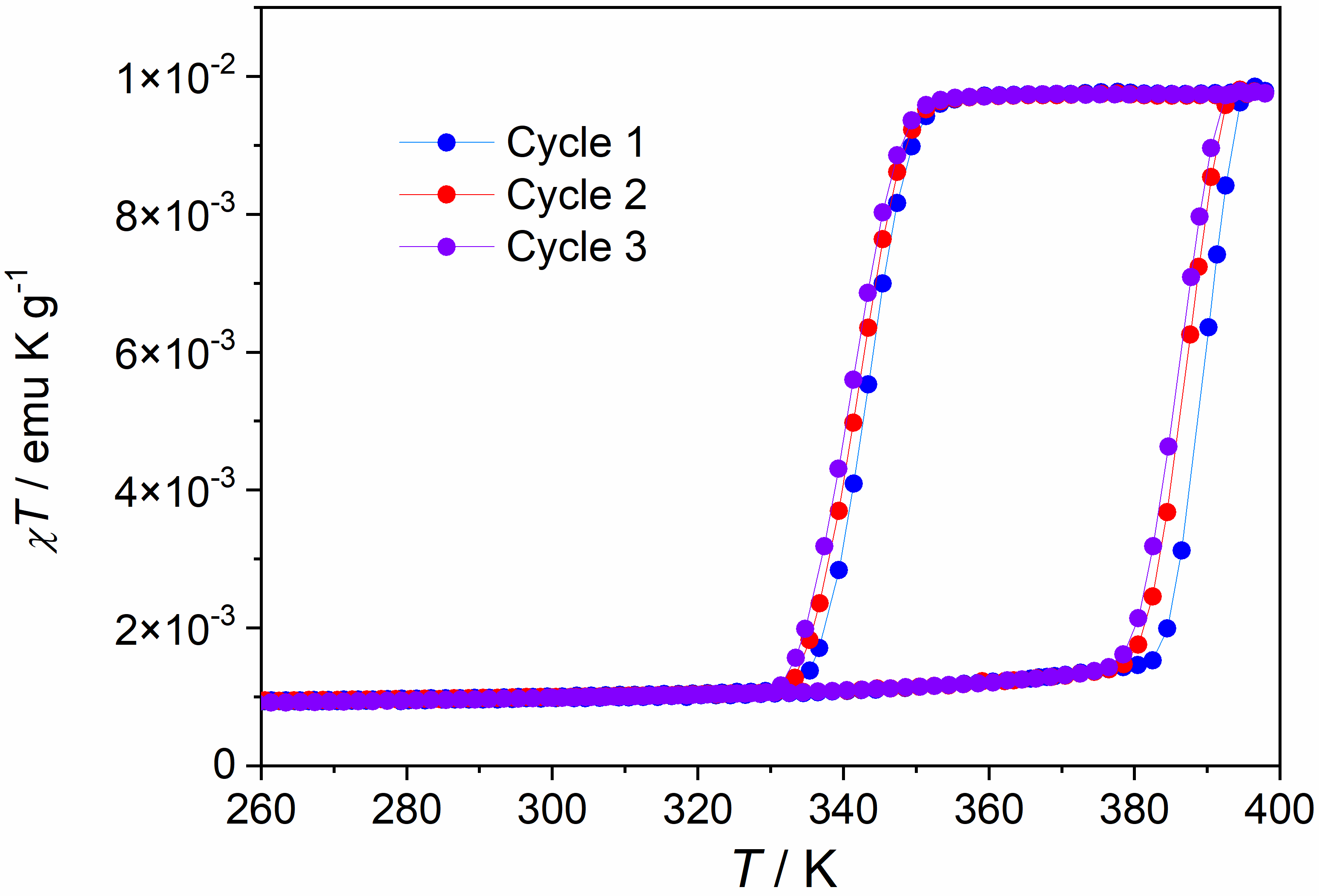


**Figure S7**. Thermal behavior of the magnetic susceptibility (*χT*) for bulk [Fe(HTrz)_2_(trz)]BF_4_ collected on three consecutive cycles.

**
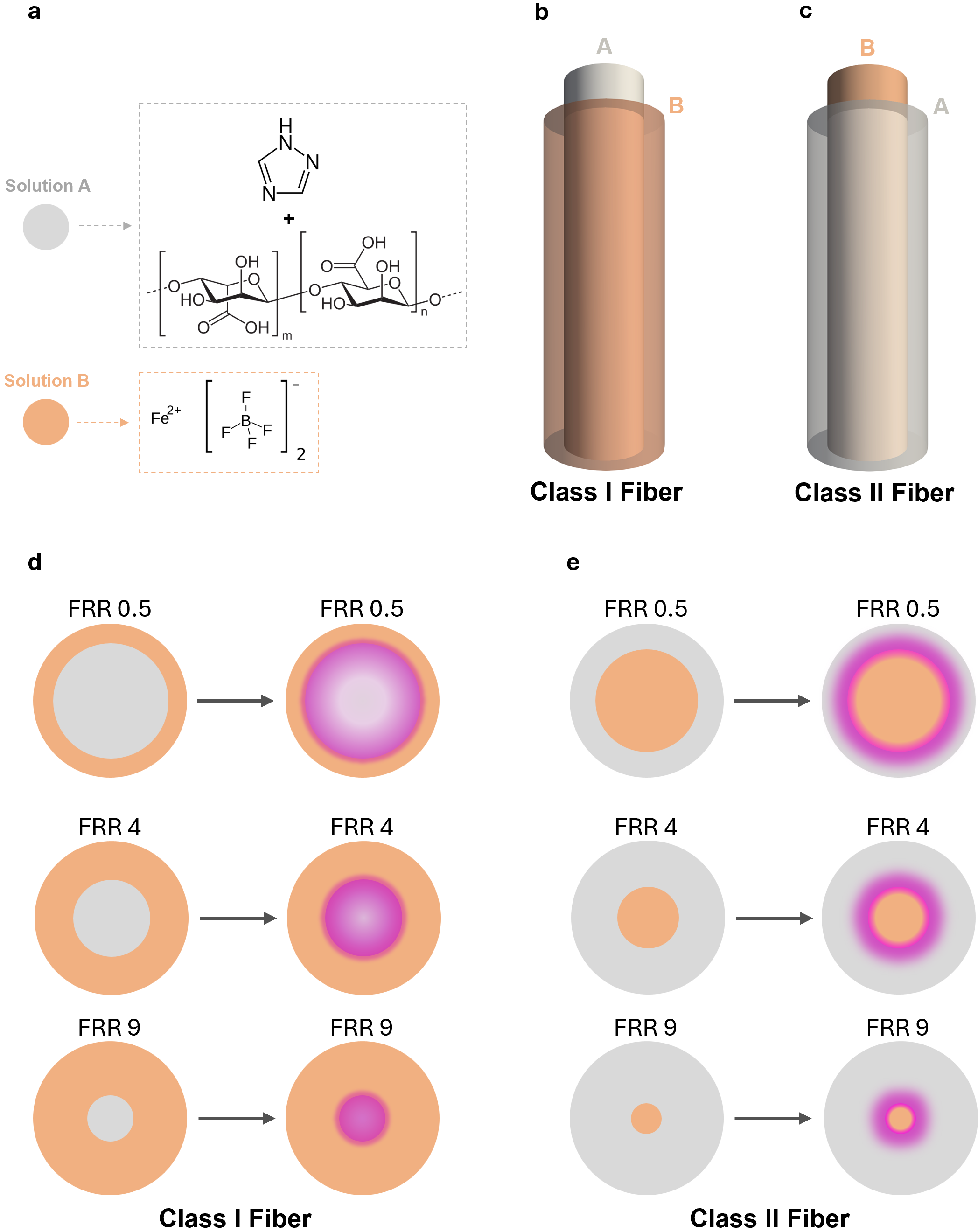
**

**Figure S8**. (a) Two solutions used to generate the SCO fibers. Solution A is a mixture of 1,2,4-triazole and sodium alginate in water, while solution B is the ethanolic solution of Fe(II) tetrafluoroborate. (b) Flow configuration to generate Class I fibers: solution A is flown through the middle inlet, while solution B is flown through the side inlets. (c) Flow configuration to generate Class II fibers: solution B is flown through the central inlet, while solution A is flown through the lateral ones. (d) Schematic representations of the RD zone (in pink color) for Class I fibers at various FRRs. Here, the left schematics illustrate the cut view of the reation chamber near the tip of the center needle, while the right ones represent the cut view at the end of the output channel. (e) Similar to (d) but for Class II fibers.


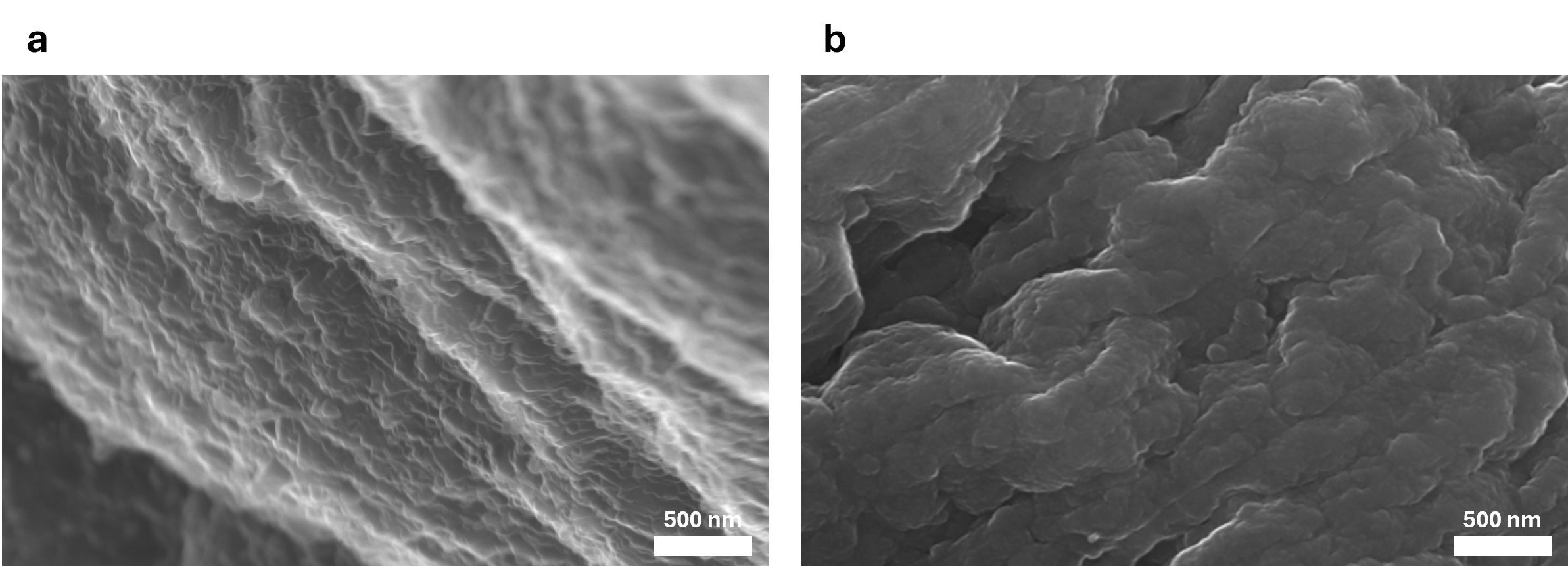


**Figure S9**. SEM images of a Class I fiber, featuring a solid topology of the biopolymer loaded with SCO nanoparticles. (a) SEM image focusing on the core of the fiber. (b) SEM image focusing on the outer shell of the fiber.


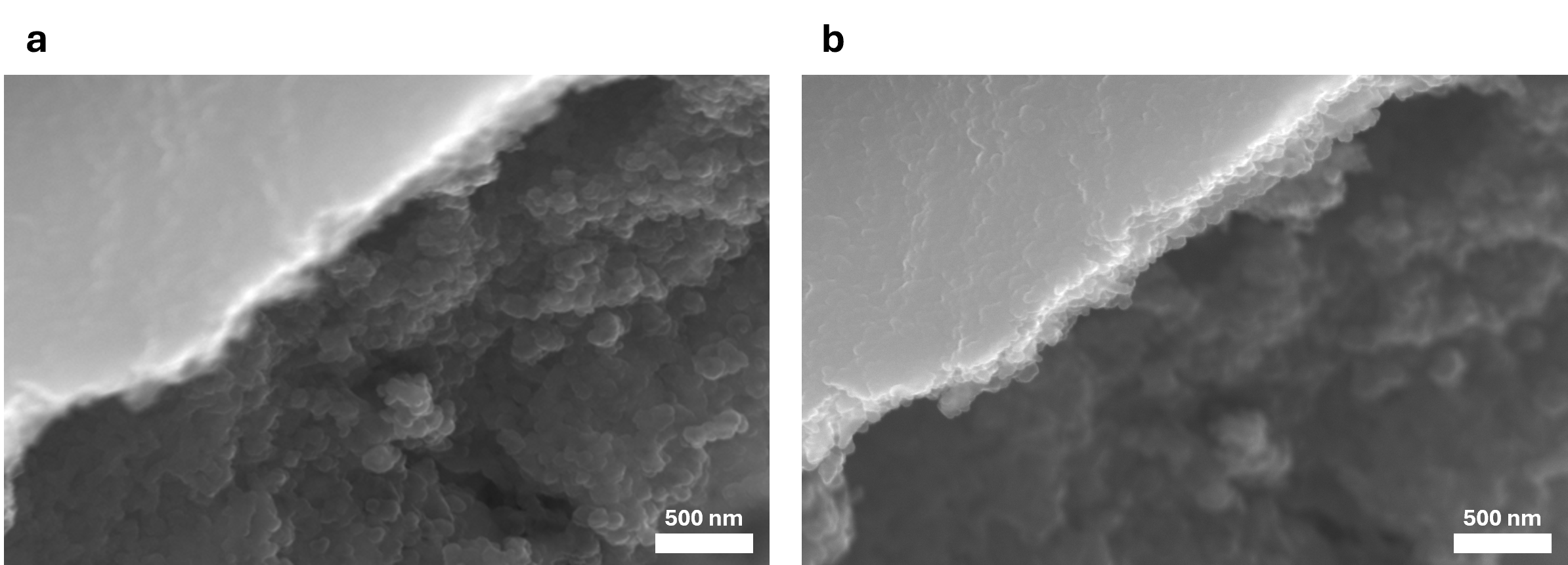


**Figure S10**. SEM images of a Class II fiber, featuring a folded hollow topology with SCO nanoparticles trapped inside the biopolymer. (a) SEM image focusing on the core of the fiber. (b) SEM image focusing on the outer shell of the fiber.


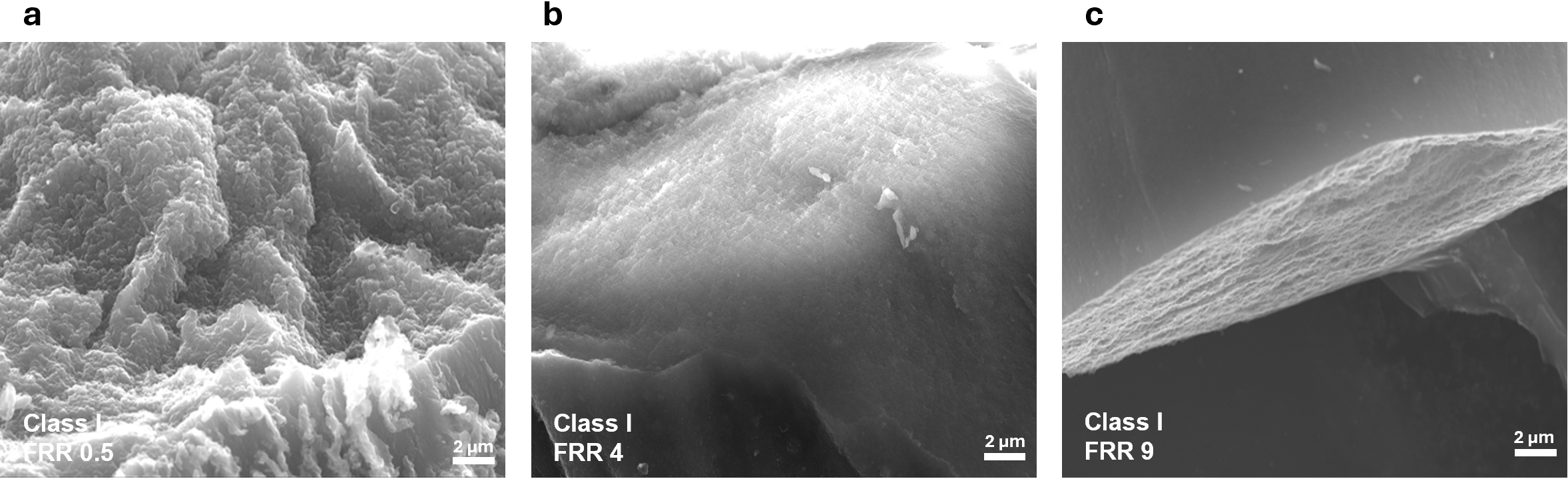


**Figure S11**. SEM images of Class I fibers synthesized with different conditions focusing on the core of the fibers: (a) FRR 0.5, (b) FRR 4, (c) FRR 9.


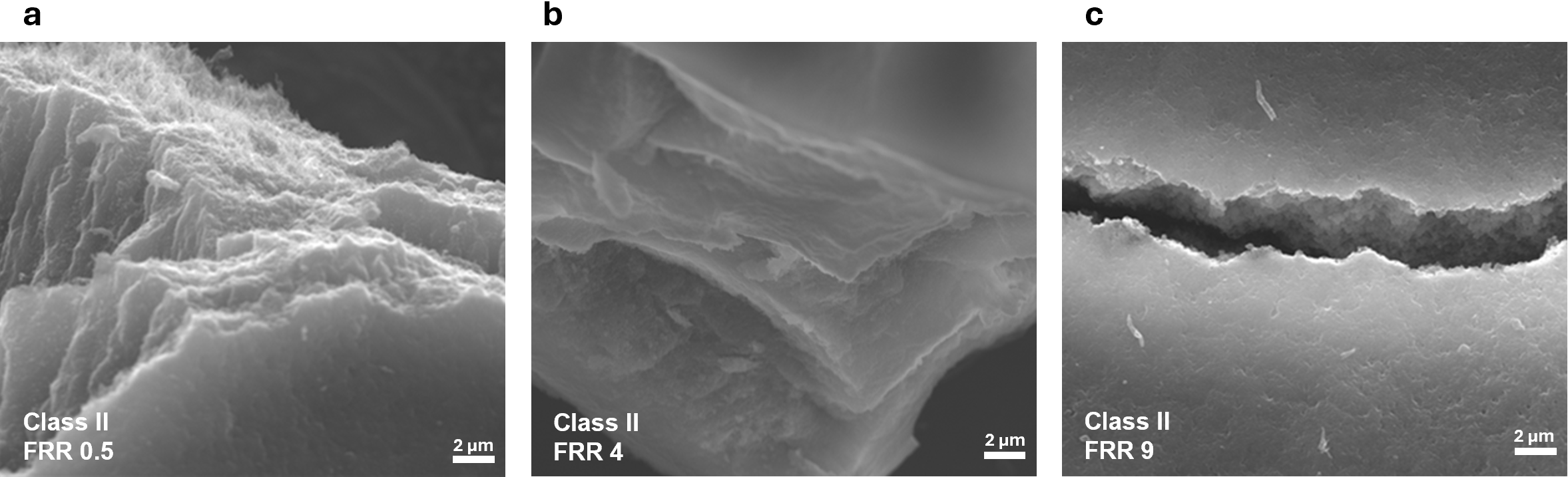


**Figure S12**. SEM images of Class II fibers synthesized with different conditions focusing on the outer shell of the fibers: (d) FRR 0.5, (e) FRR 4, (f) FRR 9.


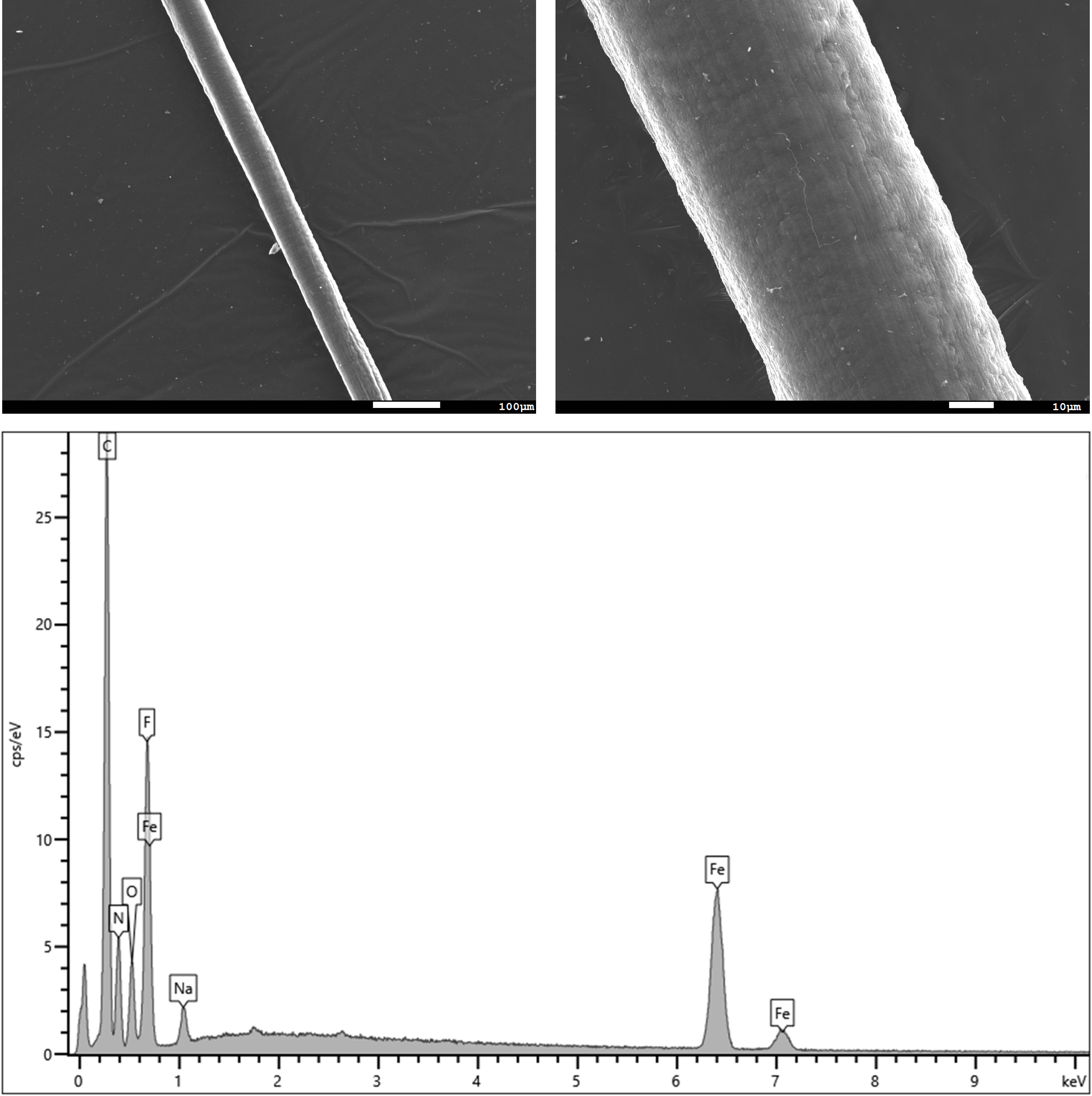


**Figure S13**. SEM images of a Class I fiber and its representative EDX analysis.


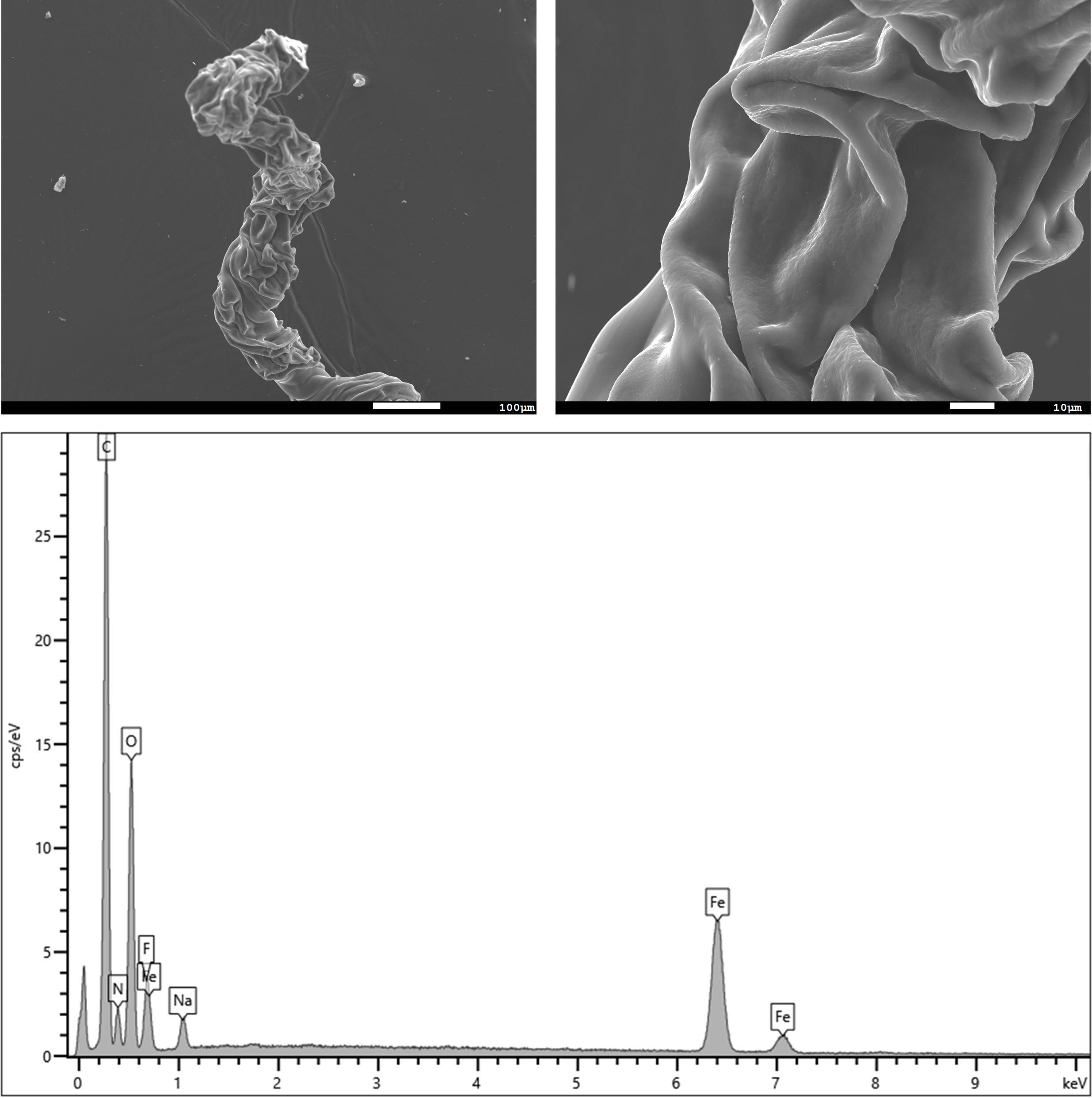


**Figure S14**. SEM images of a Class II fiber and its representative EDX analysis.


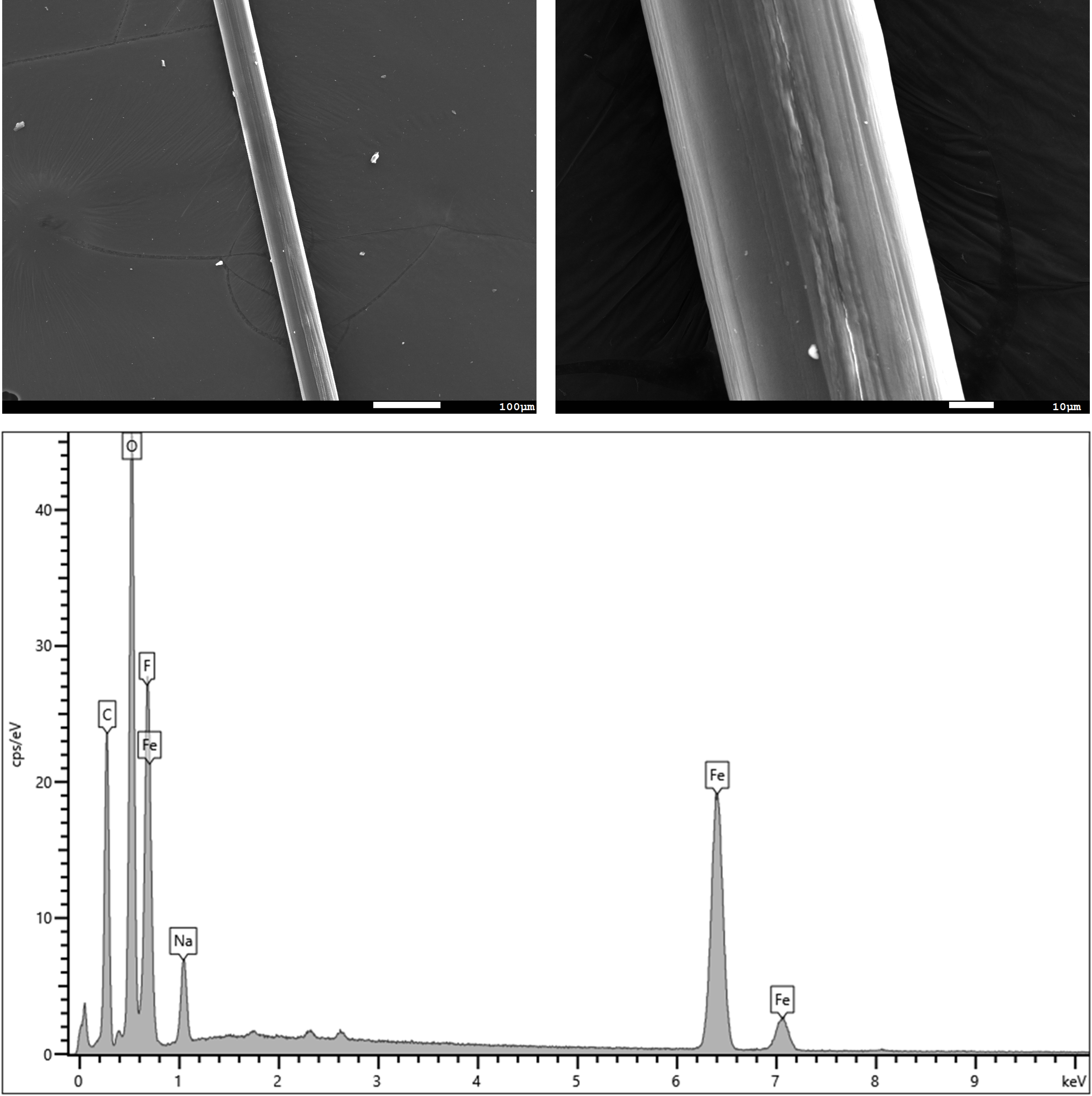


**Figure S15**. SEM images of a pure alginate fiber and its representative EDX analysis.


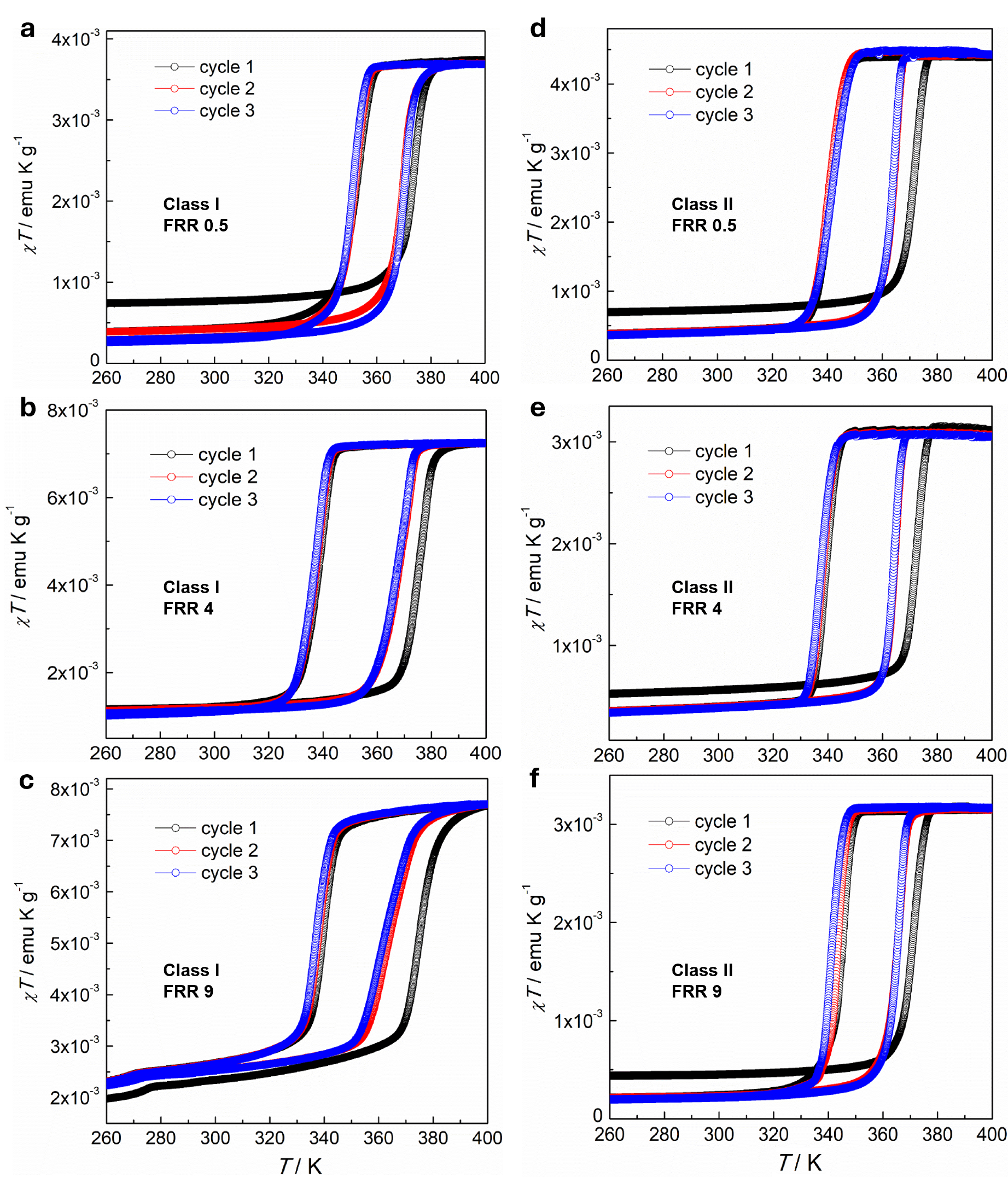


**Figure S16**. Thermal behavior of the magnetic susceptibility (χT) collected on three consecutive cycles of all the composite SCO fibers synthesized with different conditions. Class I fibers – (a) FRR 0.5, (b) FRR 4, (c) FRR 9. Class II fibers – (d) FRR 0.5, (e) FRR 4, (f) FRR 9.


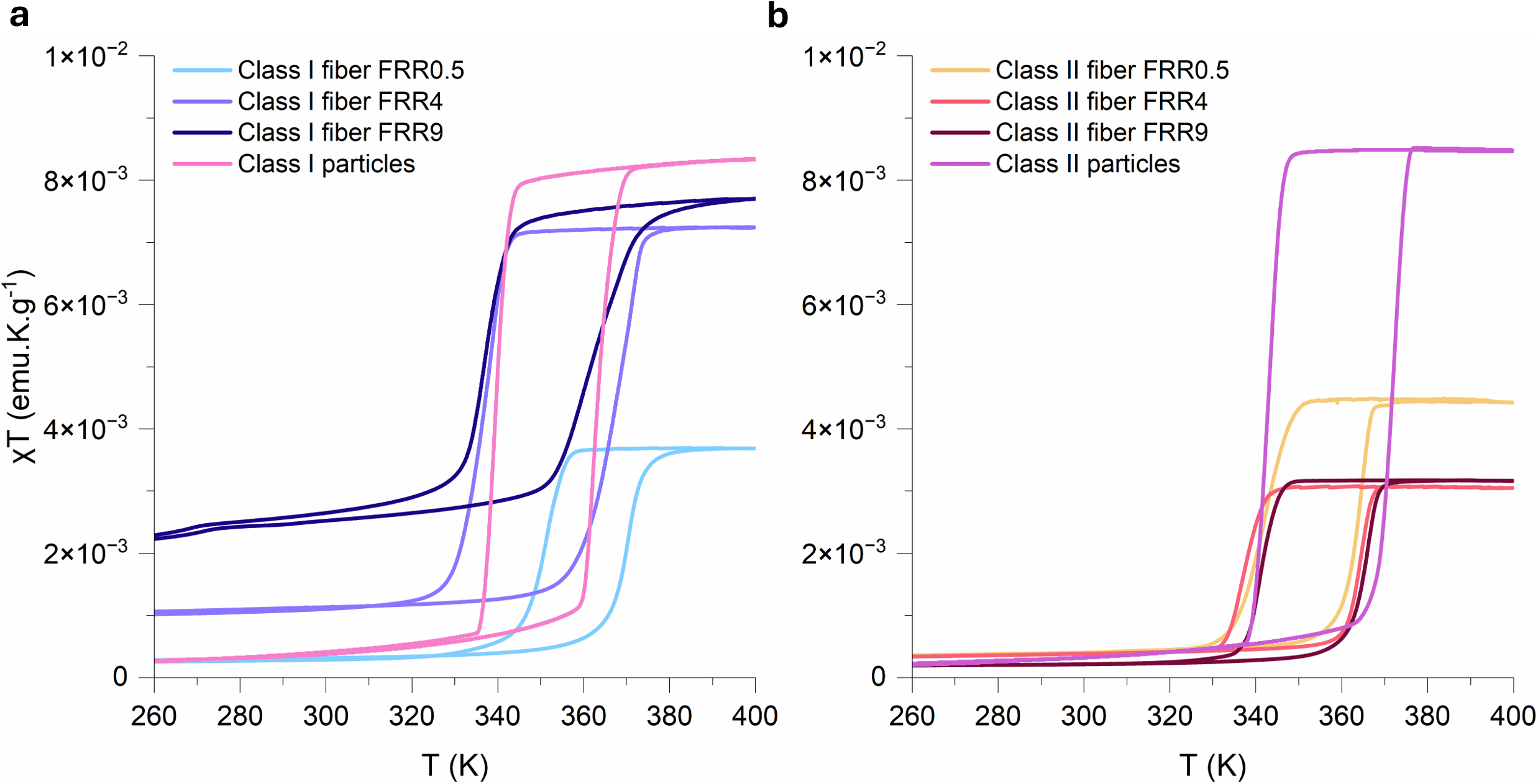


**Figure S17**. (a) Thermal behavior of the magnetic susceptibility (χT) comparison between Class I particles and Class I fibers. (b) Thermal behavior of the magnetic susceptibility (χT) comparison between Class II particles and Class II fibers.

**Table S4**. Transition temperatures observed for the different materials during the first and second thermal cycles obtained from magnetic measurements.

|  | **First thermal cycle** | | | **Second thermal cycle** | | |
| --- | --- | --- | --- | --- | --- | --- |
|  | ***T*_c_↑ (K)** | ***T*_c_↓ (K)** | **Δ*T* (K)** | ***T*_c_↑ (K)** | ***T*_c_↓ (K)** | **Δ*T* (K)** |
| **Class I fiber FRR 0.5** | 374 | 355 | 19 | 370 | 352 | 18 |
| **Class I fiber FRR 4** | 375 | 338 | 37 | 368 | 337 | 31 |
| **Class I fiber FRR 9** | 375 | 338 | 37 | 365 | 337 | 28 |
| **Class II fiber FRR 0.5** | 372 | 342 | 30 | 364 | 342 | 22 |
| **Class II fiber FRR 4** | 373 | 341 | 32 | 364 | 338 | 26 |
| **Class II fiber FRR 9** | 372 | 345 | 27 | 365 | 342 | 23 |

**Table S5**. Reaction conditions attempted for generation of Class I fibers

| **Fe(BF_4_)_2_·6H_2_O (M)** | **NaAlginate (%)** | **Htrz (M)** | **Total flow rate (µL/min)** | **FRR**  **(Class I)** | **Result** |
| --- | --- | --- | --- | --- | --- |
| 0.4 | 1 | 0.5 | 1050 | 6 | Whitish fibers |
| 0.4 | 0.5 | 2 | 1000 | 4 | Whitish fibers |
| 0.4 | 0.5 | 0.5 | 1050 | 2 | Few whitish fibers |
| 0.4 | 1 | 1 | 1050 | 2 | Some purple fibers |
| 0.4 | 1 | 2 | 1050 | 2 | Some purple fibers |
| 0.4 | 1 | 3 | 750 | 4 | Clogging |
| 0.4 | 0.75 | 2 | 250 | 4 | Clogging |
| 0.4 | 0.75 | 2 | 750 | 4 | Continuous formation of purple fibers |

Both classes of fibers display excellent long-term air stability, retaining the characteristic diffraction patterns of the initial sample after 7 days of exposure (**Figure S18a, d**). However, their chemical stability under acidic and basic conditions differs markedly. Class I fibers show limited stability at pH 2, 4, 10, and 12, as evidenced by the loss of their characteristic pink color and the emergence of an orange hue in the surrounding solution after 24 hours (**Figure S18b, e**). PXRD data further confirm this degradation, showing the disappearance of diffraction peaks associated with the SCO material. In contrast, Class II fibers exhibit significantly improved stability across all tested pH values, maintaining the diffraction pattern of the SCO particles (**Figure** **S18c, f**). This difference in chemical stability is attributed to the spatial distribution of the particles: in Class II fibers, the SCO particles are primarily embedded within the matrix, whereas in Class I fibers they are mainly surface-exposed and thus more vulnerable. Consequently, the slight coloration observed in the solution for Class II fibers likely results from the limited diffusion of acidic or basic species, which reach only the outermost SCO particles near the fiber surface, leading to partial decomposition, while the majority of the embedded particles remain intact. Interestingly, the triazole impurity disappears in all Class II samples after soaking in acidic or basic environments, likely due to water diffusion into the fiber, which dissolves the triazole and promotes its outward migration.


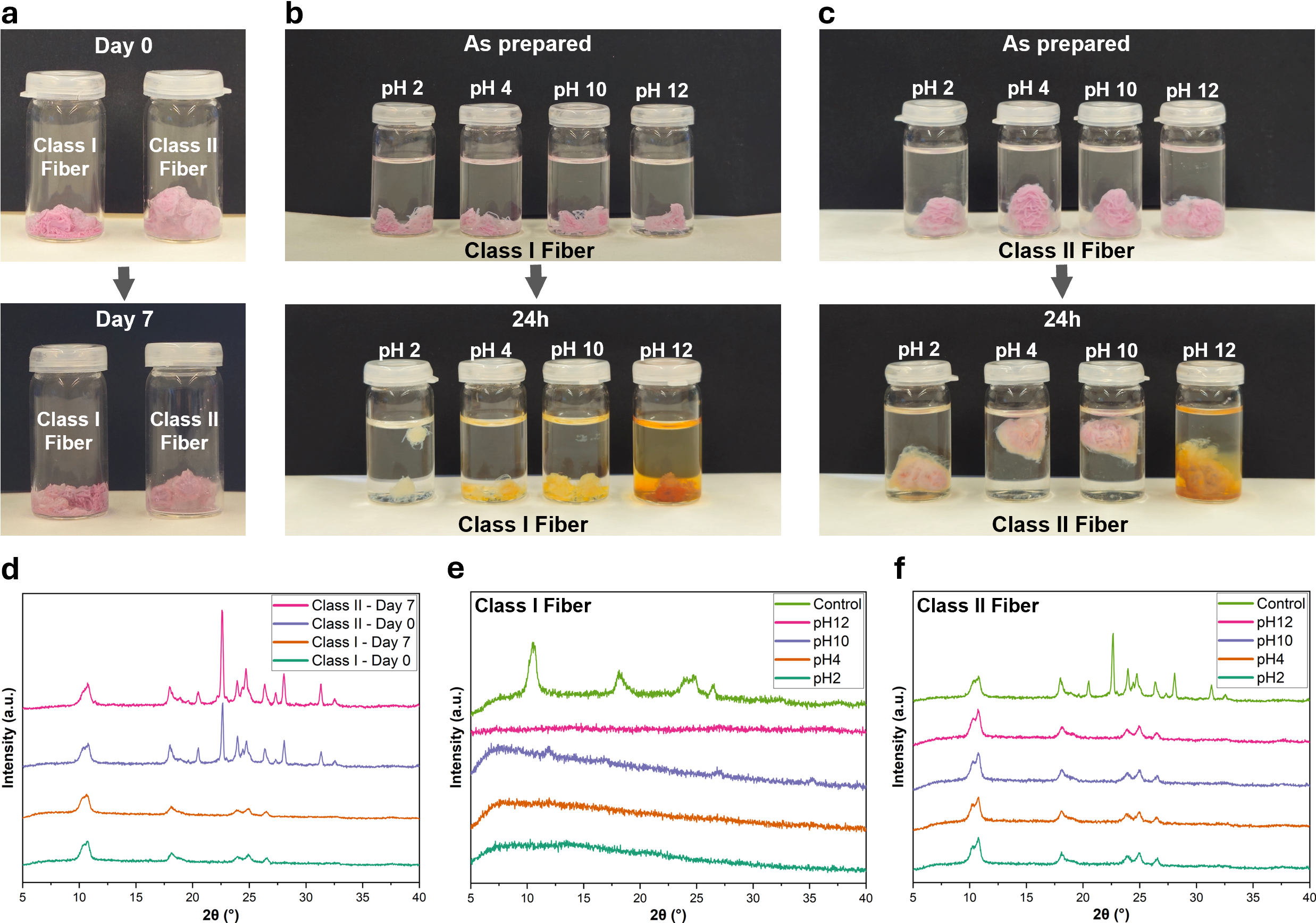


**Figure S18**. Stability test of the SCO composite fibers. (a) Stability test of the fibers in air for 7 days, with the vials’ cap closed to keep the moisture. (b, c) Stability test of Class I and Class II fibers in acidic and alkali solution for 24 hours. (d) PXRD spectra of Class I and Class II fibers on day 0 and day 7 of the storing period. (e, f) PXRD spectra of Class I and II fibers after staying in the acidic/alkali solutions for 24 hours.

Nanoindentation measurements reveal markedly different Young’s modulus values for Class I and Class II fibers (**Figure S19**). Class I fibers exhibit a modulus of approximately 499 MPa, significantly higher than the 11 MPa observed for Class II fibers. This disparity can be attributed to differences in the spatial distribution of the spin crossover SCO particles and the measurement depth of nanoindentation. Since nanoindentation probes surface and near-surface mechanical properties, the measured modulus is heavily influenced by the material composition in the top few nms. In Class I fibers, the SCO particles form a dense, surface-localized layer, leading the indenter to primarily engage with the rigid inorganic phase. The measured modulus thus reflects the stiffer nature of SCO materials, comparable to semi-crystalline polymers such as polyvinylidene fluoride (PVDF).^[3,4]^ Conversely, in Class II fibers, the SCO particles are predominantly embedded within the bulk of the polymer matrix, with the fiber surface largely composed of alginate. As a result, the modulus measurement reflects the softer mechanical response of the hydrogel phase, consistent with reported values for alginate composites and other soft elastomers such as PDMS.^[5,6]^


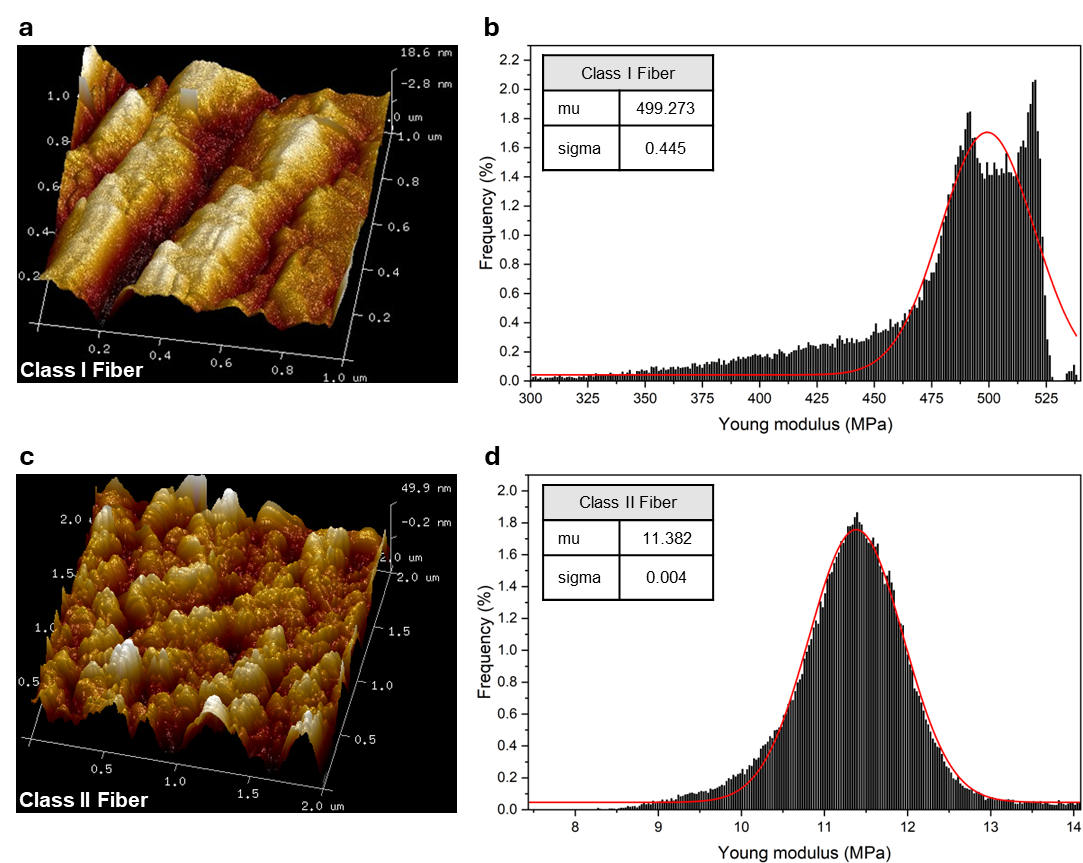
**Figure S19**. Nanoindentation measurement of Class I and Class II fibers. (a, c) Tomography images of the region of interest (ROI) from Class I and Class I fibers. (b, d) Statistics of Young modulus measurement of the ROIs from class I and Class II fibers..

Variable-temperature XRD data for Class I and II fibers shows a similar trend for both systems (**Figure S20**). Upon the LS to HS transition, both fiber classes exhibit lattice expansion, as evidenced by a shift in the diffraction peaks towards lower 2θ angles. This transition also appears to be associated with the disappearance of the third peak in the triplet located at approximately 2θ ≈ 26°. When the samples are cooled back to room temperature, the original crystalline structure is recovered. This analysis suggests that the spin transition does not involve an irreversible crystallographic change and aligns with the spin transtions previously observed in polymorphs I and II.^[2]^ However, due to the resolution of the data, it is not possible to determine whether the space group is preserved above the transition temperature. Additionally, in the case of Class II fibers, heating the sample to high temperatures (110–120 °C) results in the disappearance of the peaks associated with the triazole impurities. This is attributed to the melting of the triazole impurities, which do not recrystallize upon cooling.


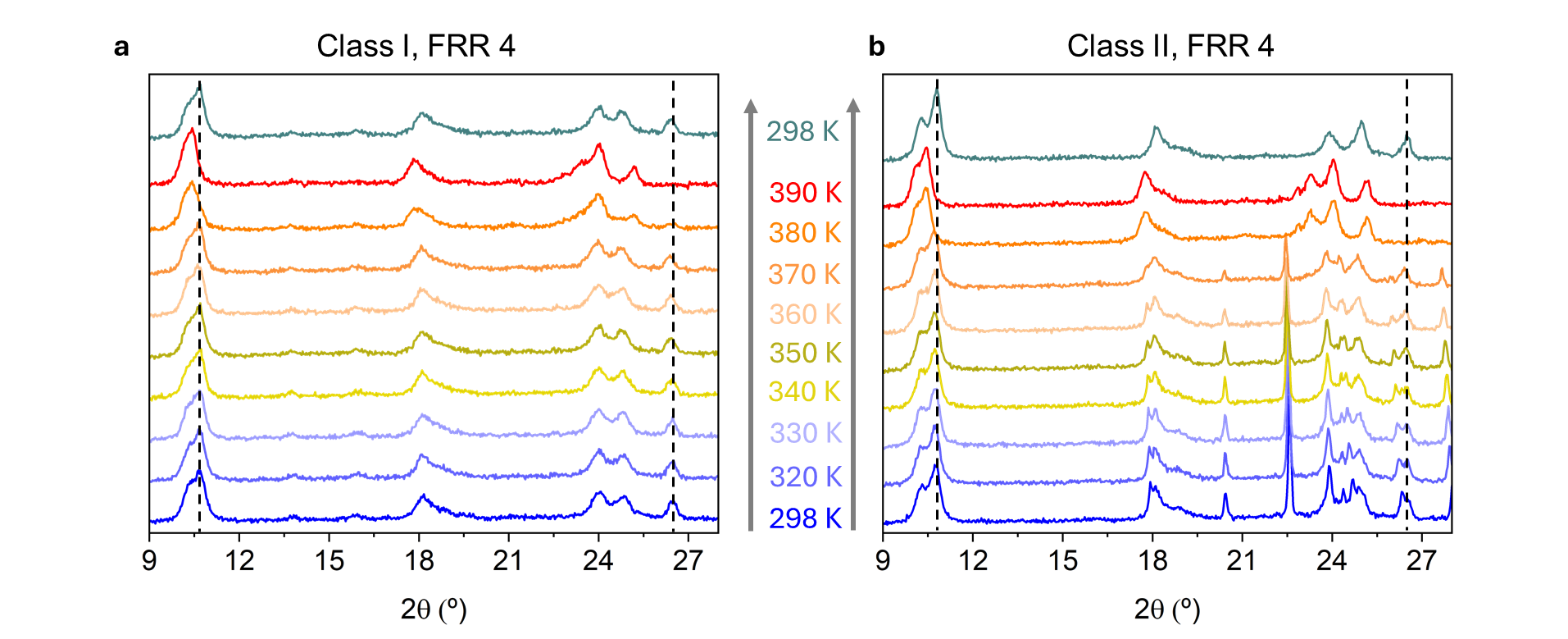


**Figure S20**. Variable temperature XRD for Class I (a) and Class II (b) fibers.

# S2. Simulation Section

## Fluid properties for SCO complex and composite generation

**Solution A**: 0.75% w/v of Na Alginate and 2 M of Htrz in water. After mixing everything, the solution is quite viscous, but the viscosity does not change significantly with shear rate (ranging from 17 to 18 mPa.s, see **Figure S21**). Therefore, we assumed a constant viscosity in numerical simulations (17.8 mPa.s). However, in simulations that considered only the formation of SCO particles without the presence of alginate, the viscosity of solution A was assumed to be that of water (1 mPa.s).

**Solution B**: 0.4 M of Fe(BF_4_)_2_·6H2O in ethanol. Viscosity was assumed to be that of ethanol at room temperature: 1.2 mPa.s ^[7]^.

| Reagents | Diffusion coefficients | References |
| --- | --- | --- |
| Htrz | $1.91\times{10}^{-9} m^{2}/s$ | ^[8]^ |
| Sodium alginate | $1\times{10}^{-20} m^{2}/s$  (Insoluble in ethanol) | ^[9]^ |
| Fe^2+^ | $0.719\times{10}^{-9} m^{2}/s$ | ^[10]^ |

**Table S6**. Diffusion coefficient considered for each reagent in numerical simulations.


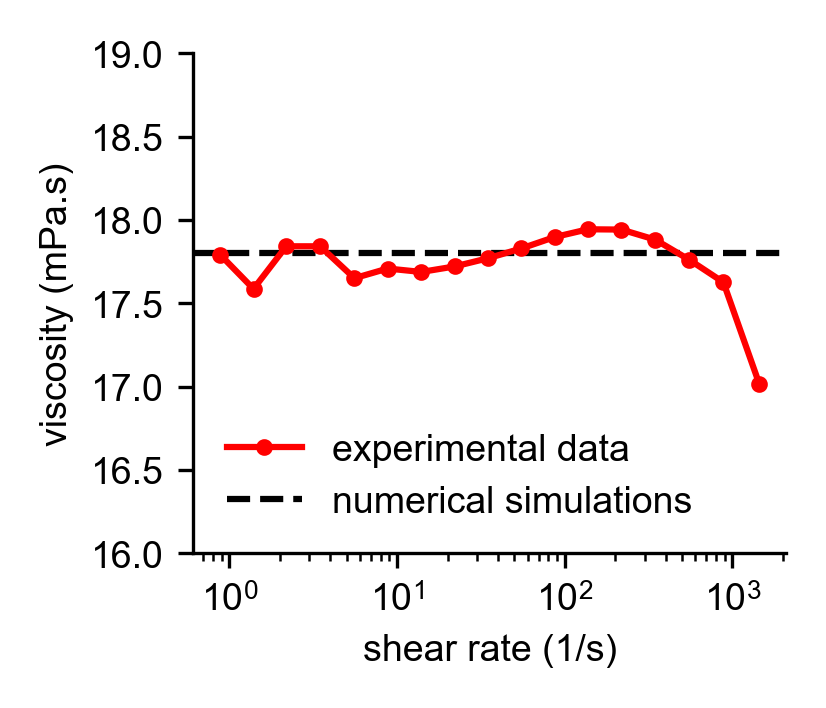


**Figure S21**. Viscosity of alginate solution (solution A) at various shear rates. Red dots represent the experimental data, and the black dashed line represents the constant viscosity that was assumed in numerical simulations.

## Geometry and boundary conditions

Because the flow and mass transport in the main chamber of our microfluidic device are symmetric around the central axis of the device (**Figure S22a**), we performed numerical simulations considering an axisymmetric 2D geometry (**Figure S22b**), without loss of accuracy or representativeness of the results. Mimicking the experiments that were performed, solutions A and B were continuously introduced either through the central or side the inlets at various flow rates. No-slip condition was assumed at all the walls of the microfluidic device, and zero gradient was assumed at the outlet.


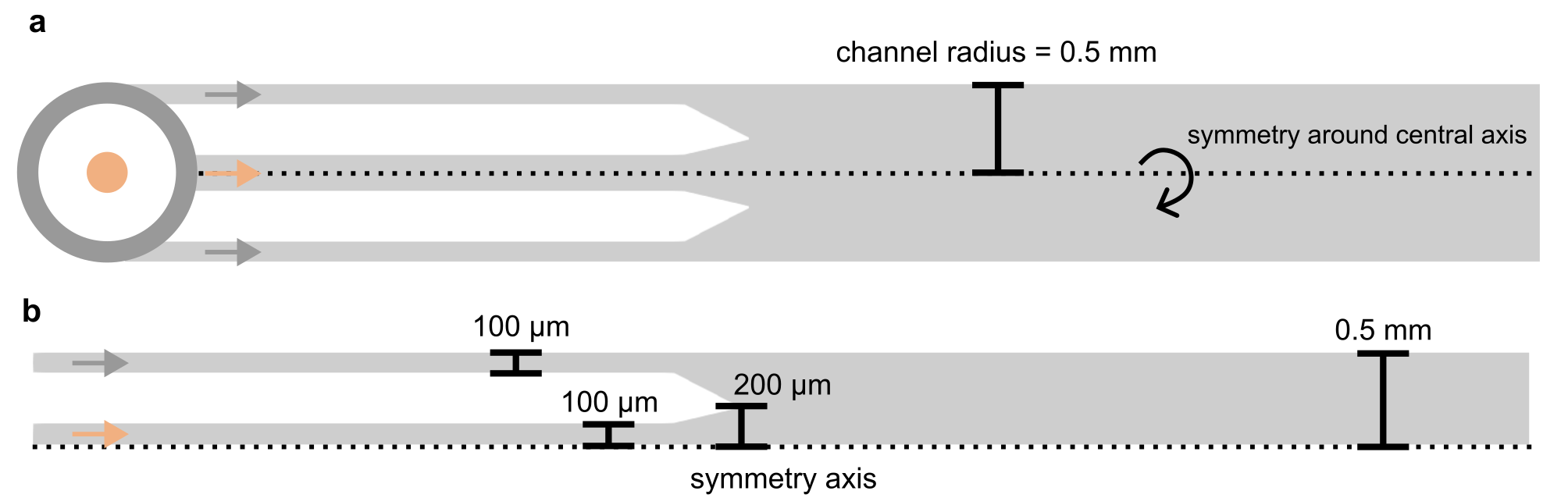


**Figure S22**. Representation of the simulation domain, main dimensions and symmetry axis.

## Numerical methods

A computational fluid dynamics approach was used to simulate the flow and mass transport inside the microfluidic device. Specifically, the velocity, pressure, and species concentration were calculated using the finite volume method by coupling the Navier-Stokes equation for incompressible Newtonian fluids, the continuity equation, and the species transport equation that are respectively given by:

$$\frac{\partial\vec{V}}{\partial t}+\vec{V}\left( \nabla\cdot\vec{V} \right)=-\frac{1}{\rho}\nabla P+\upsilon\nabla^{2}\vec{V}$$

$$\frac{\partial\rho}{\partial t}+\nabla(\rho\vec{V})=0$$

$$\frac{\partial(\rho Y_{i})}{\partial t}+\nabla\left( \rho\vec{V}Y_{i} \right)={\rho D}_{i}\nabla^{2}Y_{i}$$

where $\vec{V}$ is the velocity vector, *P* is the pressure, ∇ is the divergence operator, $\nabla^{2}$ is the Laplacian operator, *ρ* is the fluid density, *υ* is the viscosity, *Y_i_* is the mass fraction of species i and *D_i_* is the diffusion coefficient of species i. A steady-state, double precision solver was employed to solve these equations considering the SIMPLE algorithm for pressure-velocity coupling and using second order upwind discretization.

Since the transport of solutes (Htrz, Fe^2+^) does not significantly affect the viscosity of the solutions nor the velocity / pressure fields inside the device, we solved the equations above using a two-step approach. First, we coupled the momentum and continuity equations with the mass transport equation for solution A. Because alginate is not soluble in ethanol (*D_alginate_* = 1 × 10^-20^ m^2^/s), the species transport equation used to calculate the fraction of solution A and B can be reduced to:

$$\nabla\left( \rho\vec{V}Y_{A} \right)=0$$

with:

$$Y_{B}=1- Y_{A}$$

where *Y_A_* and *Y_B_* are the mass fraction of solution A and B, respectively. In a second step, we used the steady-state solution for velocity, pressure and fraction of solution A and B, obtained in the first step, to solve scalar transport equations for each solute (Htrz, Fe^2+^), which, in steady-state, are represented by:

$$\nabla\left( \vec{V}C_{i} \right)=D_{i}\nabla^{2}C_{i}$$

where *C_i_* is the concentration of solute i.

## Mesh independence testing and validation

We performed preliminary simulations using different meshes to identify the coarsest mesh that could produce mesh-independent results. These simulations were performed for a total flow rate of 750 µL/min considering a constant viscosity of 1.2 mPa∙s. We found that the velocity and Hrtz profiles along the radius of the device (**Figure S23**) obtained using a mesh containing 200 000 cells and considering residuals lower than 10^-4^ as convergence criteria, were similar to those obtained using a mesh containing 2 000 000 cells and residuals lower than 10^-6^. Moreover, the velocity profile obtained using a mesh containing 200 000 cells and considering residuals lower than 10^-4^, was similar to that predicted by the theoretical solution for velocity profile in circular pipes.^[11]^ Based on this results, re ran all simulations with a mesh contained around 200 000 cells (**Figure S24)**, and we assumed that convergence of the simulations when residuals were lower than 10^-4^.


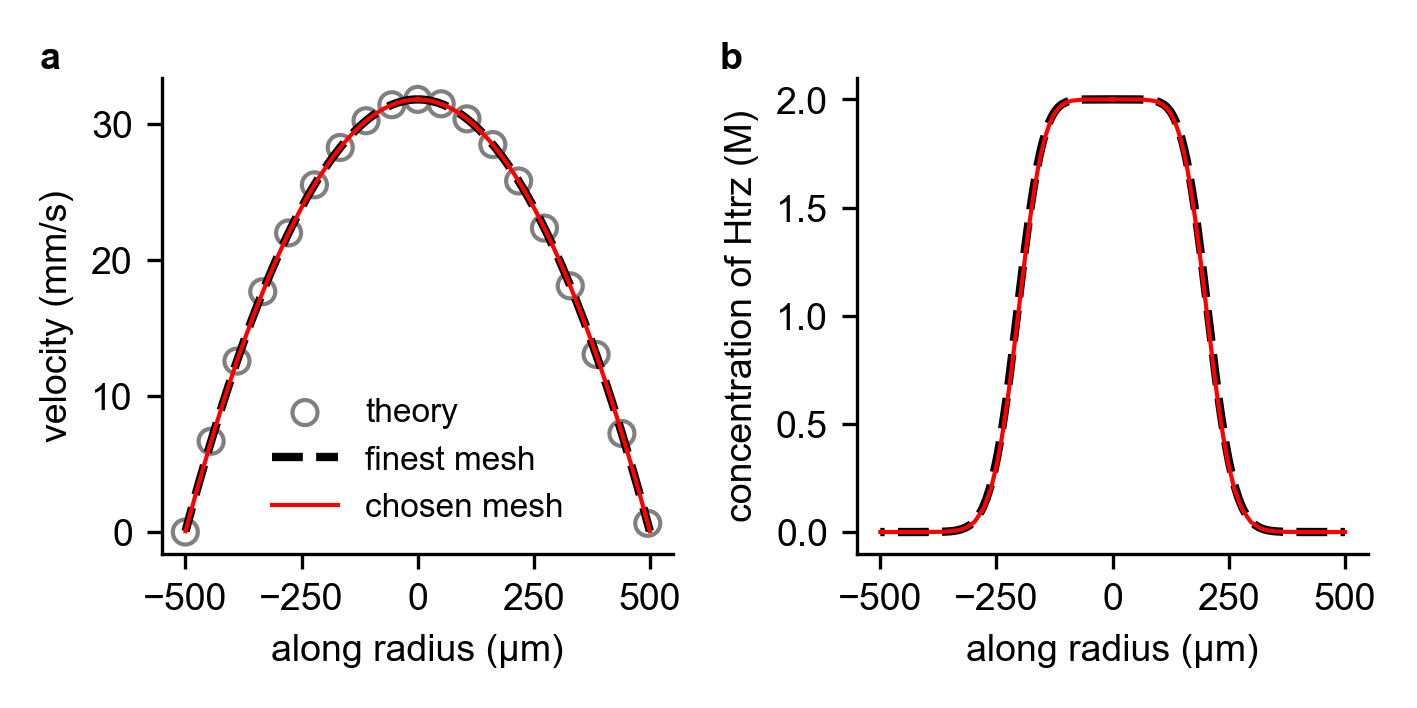


**Figure S23**. Simulations for mesh independence testing and validation. (a) Velocity and (b) Htrz concentration profiles along the radius at the outlet of the microfluidic device considering a viscosity of 1.2 mPa∙s and TFR of 750 µL/min. The theoretical solution for velocity profile is valid for laminar flows in circular pipes.^[11]^ The finest mesh consists of around 2 000 000 cells and its solution was considered converged when residuals were lower than 10^-6^. The chosen mesh consists of around 200 000 cells and its solution was considered converged when residuals were lower than 10^-4^.


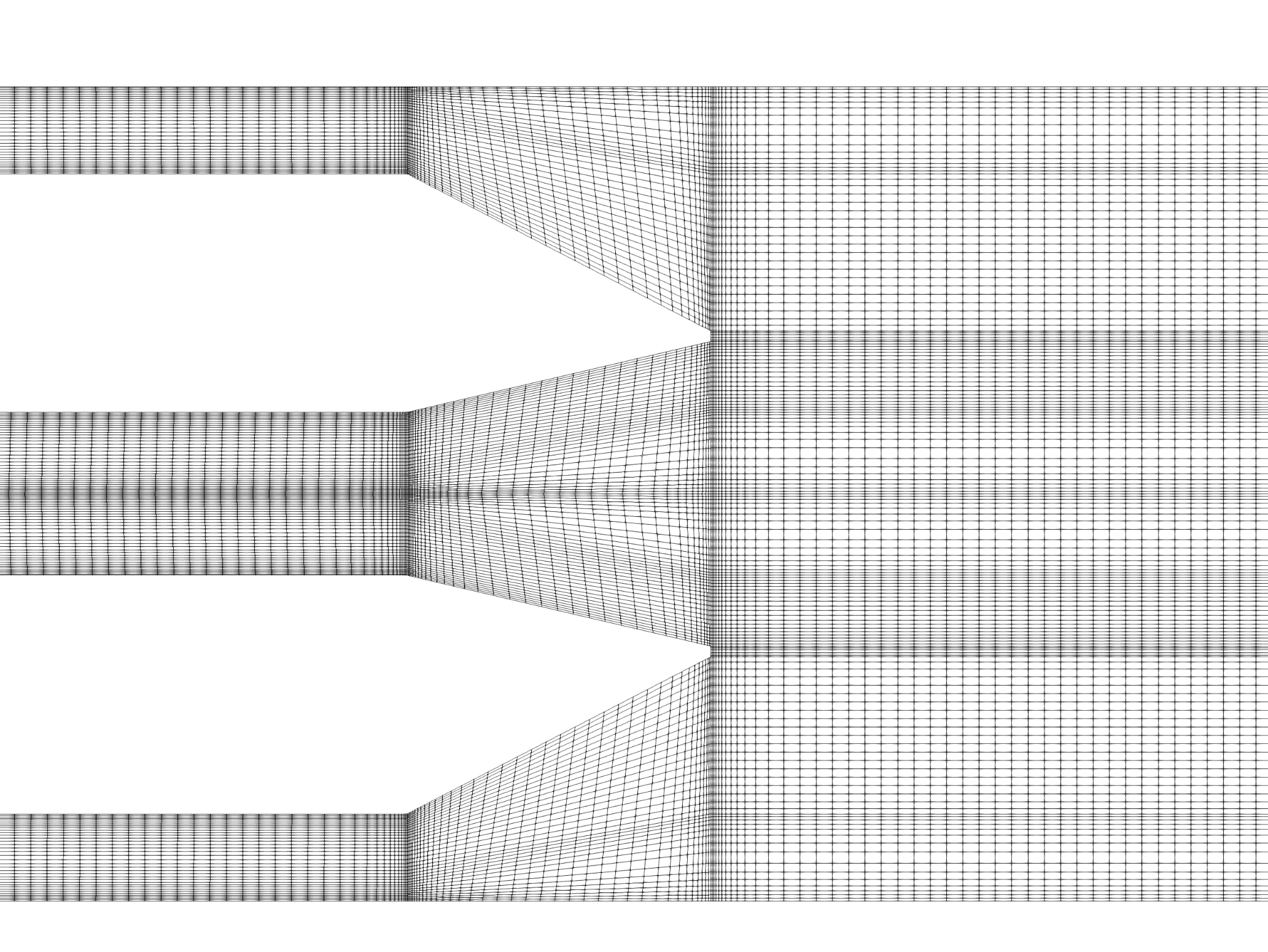


**Figure S24**. Representation of the chosen mesh near the inlets of the microfluidic device.

## Results

***Class I and II particles***

Initially, numerical simulations of flow and mass transport in the microfluidic device were performed considering the formation of SCO particles without the presence of alginate. In simulations of class I particle formation, a solution with Htrz is inserted into the central inlet and is focused by the side flows containing Fe(II). Inversely, for class II particle formation, Fe(II) is inserted into the central inlet and is focused by the side flows that, in this case, contain Htrz. The concentration profiles of these two species inside the microfluidic device are very different between class I and class II particles (**Figure S25**) because of the different inlet position of the species in both cases. Furthermore, these two chemical species will diffuse radially at different rates (proportional to their diffusion coefficient, see **Table S6**) along the microfluidic channel and, depending on the FRR that is used, may or may not occupy the entire cross-section of the device upon reaching the outlet (**Figure S26**). For example, for class I particles, using a FRR of 0.5 leads to Htrz diffusing to the entire cross-section of the device (1000 µm) but, in those conditions, Fe(II) only occupies around 650 µm along the width of the device. Instead, a FRR of 4 can be used to enable both Htrz and Fe(II) to diffuse to the entire width of the device. This indicates that the transport of Htrz and Fe(II) in the device, and their subsequent mixing and reaction to form SCO particles, can be controlled using our microfluidic approach by changing flow parameters such as the FRR.


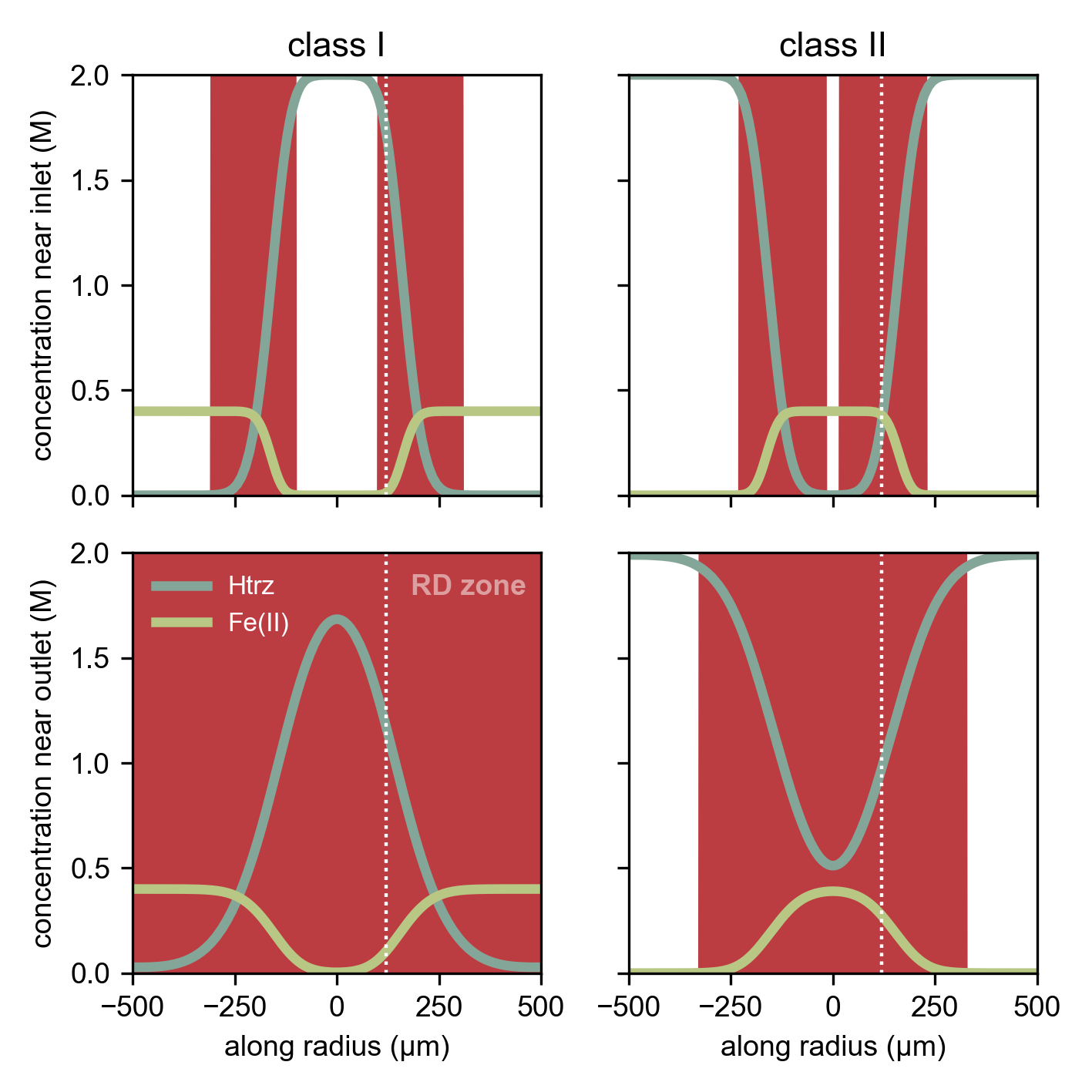


**Figure S25**. Concentration profiles of Htrz and Fe(II) along the radial direction for class I (left plots) and class II (right plots) particle formation near the inlet (upper plots, 10 mm into the main microfluidic channel) and near the outlet (lower plots, 60 mm into the main microfluidic channel). The RD zone along the channel radius in which Htrz and Fe(II) concentrations exceed 1 mM are represented in red. The dotted vertical line represents an arbitrary position along the channel radius in which significant differences in Htrz and Fe(II) concentration can be observed when we compare plots for different particle class or position along the device (near inlet/near outlet).


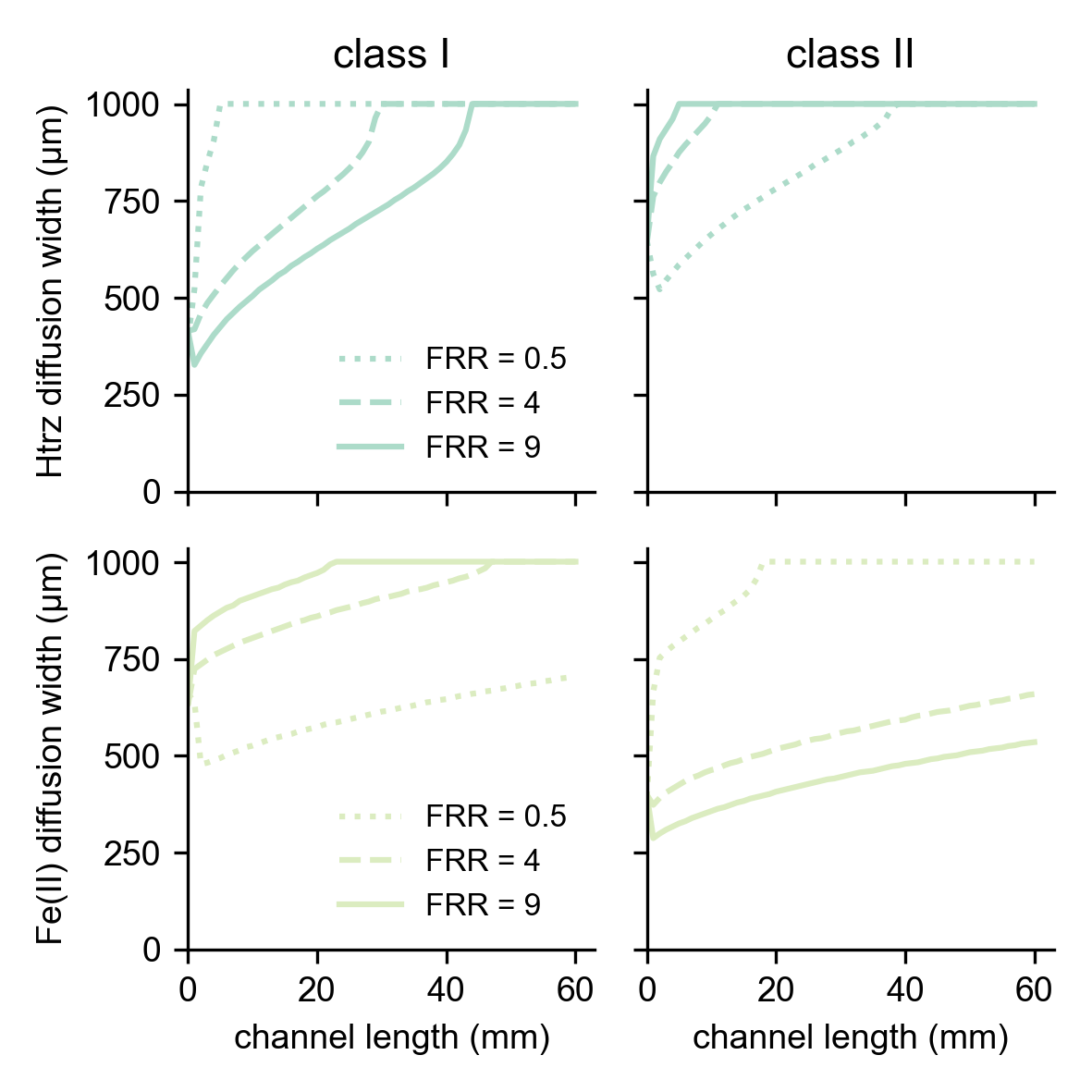


**Figure S26**. Diffusion width of Htrz and Fe(II) along the microfluidic channel length for class I and class II particle formation at various flow rate ratios.

The FRR can also be leveraged to control and maximize the RD zone for particle formation in the device, i.e. the region in which there is a high concentration (> 1 mM) of both Htrz and Fe(II). Interestingly, our simulations showed that FRR = 4 leads to a larger overall RD zone along the microfluidic channel than FRR = 9, for both Class I and Class II particle formation (**Figure S27**). This indicates that using FRR = 4 may be preferable when looking to maximize throughput. Note that although FRR = 0.5 led to a larger RD zone than FRR = 4 for class II particle formation, such a low FRR can cause clogging.


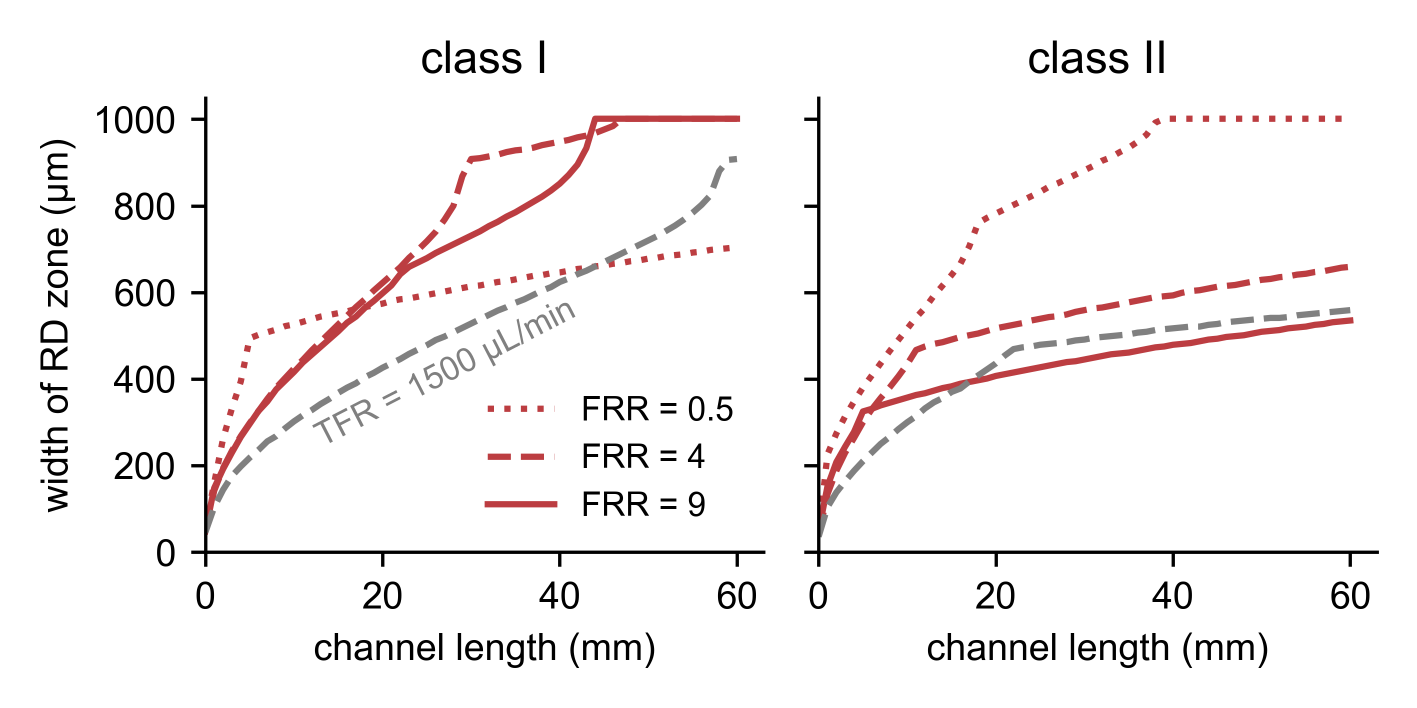


**Figure S27**. RD zone width for the formation of SCO particles (class I and class II) along the microfluidic channel length at various flow rate ratios.

In **Figure S28** and **Figure S29** we represented the regions at the outlet of the microfluidic device where the concentrations of Htrz and Fe(II) are above the minimum threshold (> 1 mM) considered for acknowledging the RD zone. Then, we represented in red the RD zone where particle formation primarily takes place, i.e. where both Htrz and Fe(II) > 1 mM. These simulation results indicate that Htrz occupies the entire outlet of the microfluidic device for all conditions tested, but that Fe(II) only does so in specific conditions (i.e. with high FRR for class I and low FRR for class II). This is because Htrz has a much higher diffusion coefficient than Fe(II) (**Table S6**). Furthermore, results indicate that the location of the RD zone for particle formation will correspond to the regions where Fe(II) is present (being thus limited by its diffusion rate) given that Htrz occupied the entire cross-section of the device in every scenario tested. Finally, simulations allowed us to calculate the approximate width of the RD zone for SCO particle formation at various FRR (**Figure S30**). The width of the RD zone can be as low as 500 µm in class II particle formation at FRR = 9, or up to 1000 µm (i.e. the diameter of the channel) at FRR = 0.5 in class II particle formation and at FRR ≥ 4 in class I particle formation. This demonstrates that the flow conditions allow us to easily leverage the characteristics of the RD zone in which SCO particles are generated.


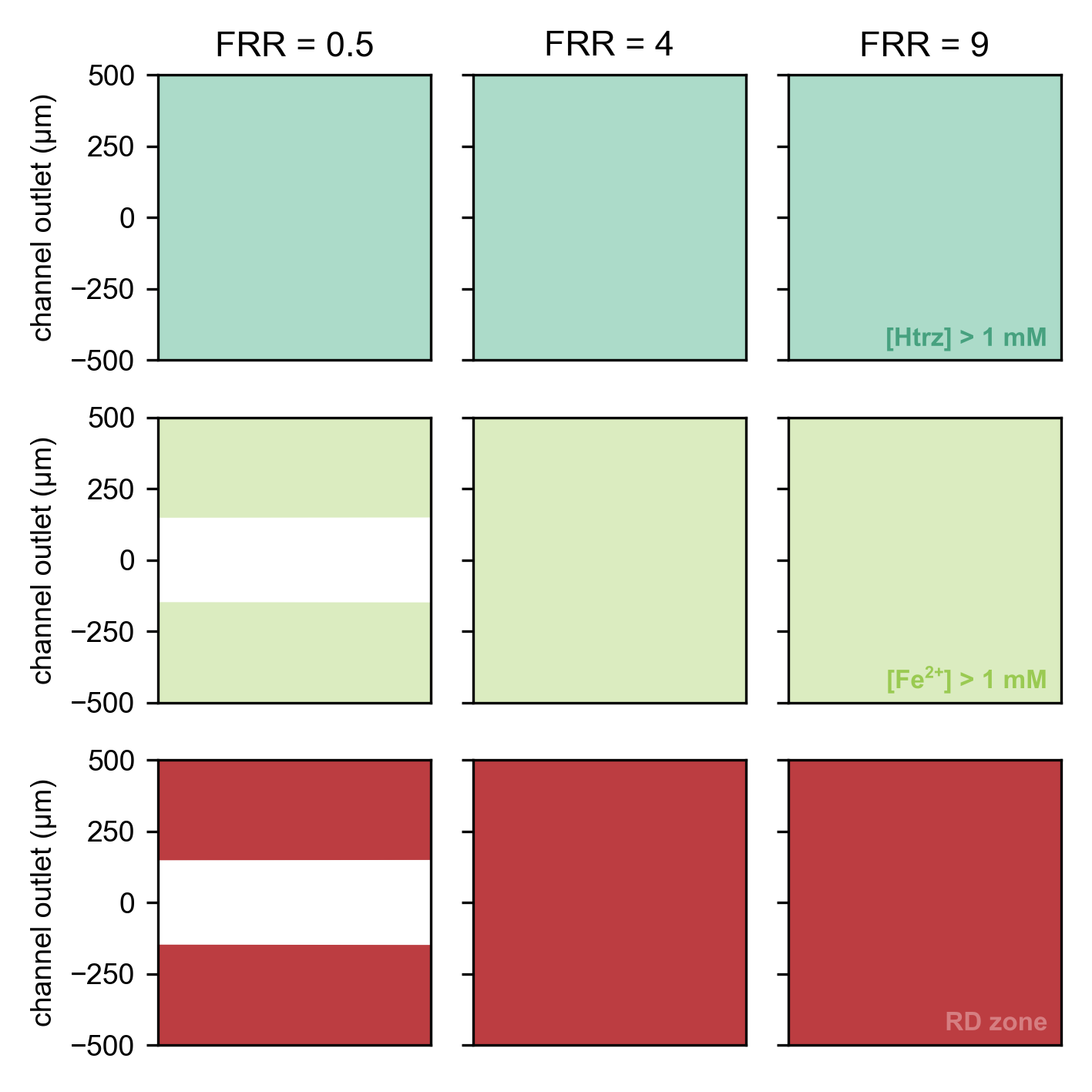


**Figure S28**. Positions along the radius of the channel outlet where each chemical species and RD zone are located for class I particle formation: [Htrz] > 1 mM, [Fe^2+^] > 1 mM and RD zone.


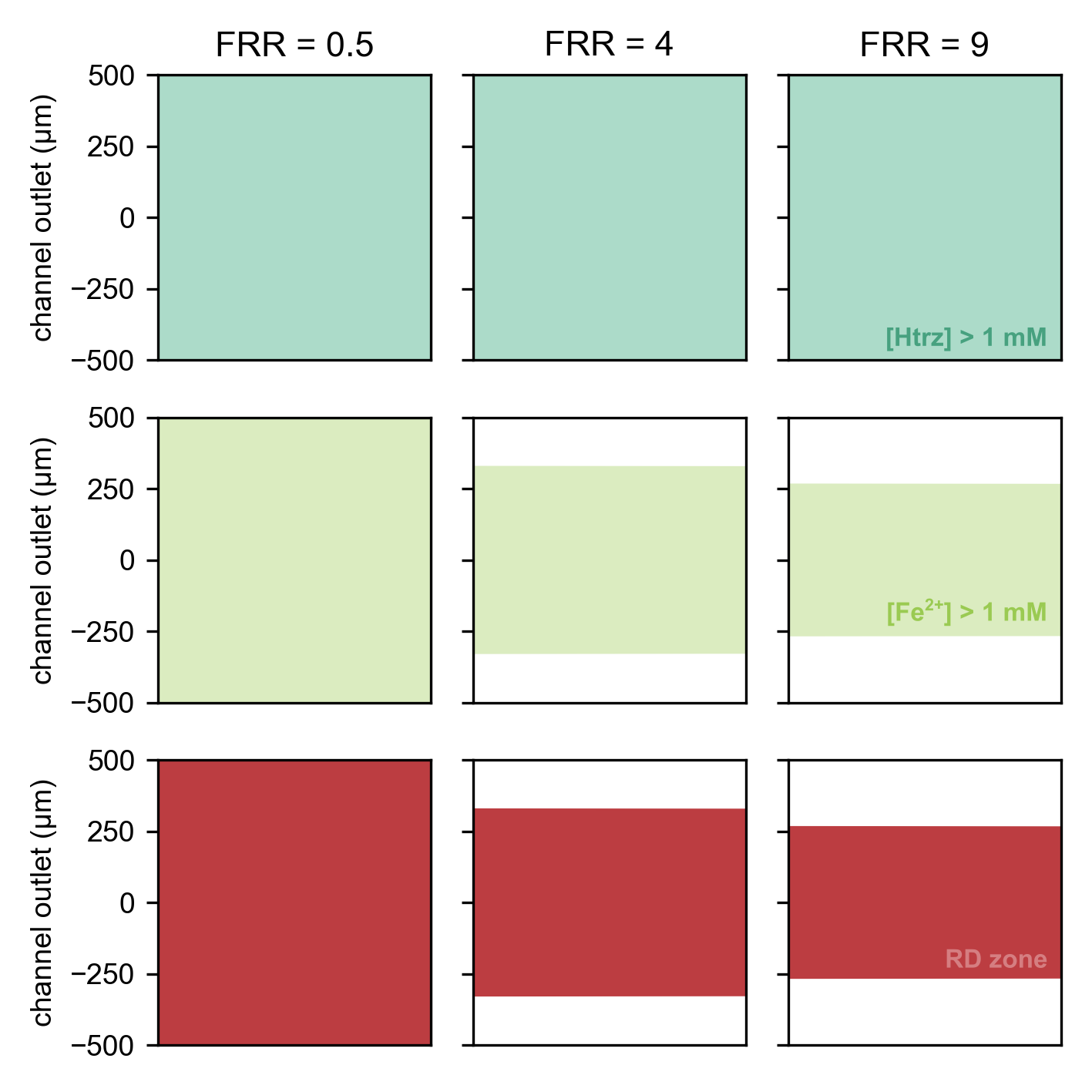


**Figure S29**. Positions along the radius of the channel outlet where each chemical species and RD zone are located for class II particle formation: [Htrz] > 1 mM, [Fe^2+^] > 1 mM and RD zone.


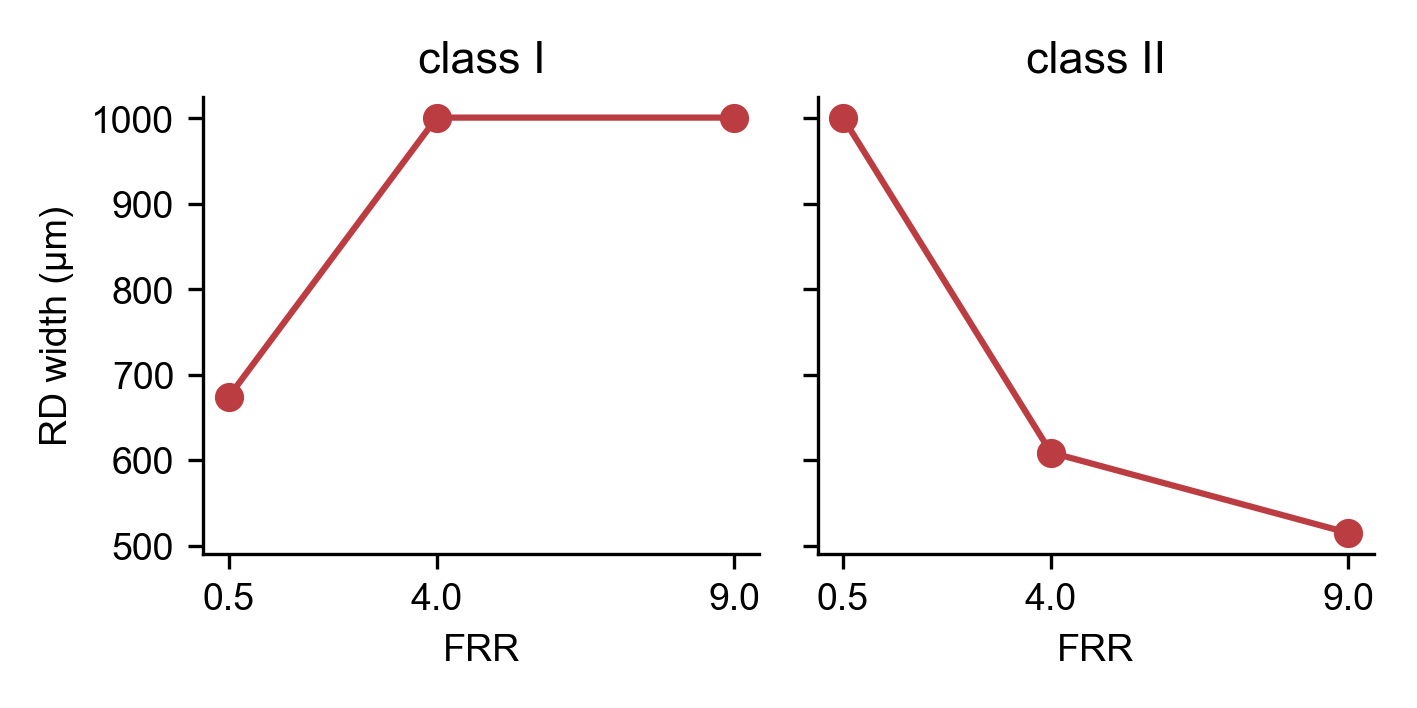


**Figure S30**. RD width in the generation of class I and class II particles using various flow rate ratios.

***Class I and II fibers***

Numerical simulations of flow and mass transport in the microfluidic device were performed at increasing values of FRR for the formation of class I and class II fibers. Note that the results of these simulations are slightly different from those performed in the previous section for particle formation, because, in the previous section, the lack of alginate meant that a different viscosity was considered in simulations.

For class I fibers, solution A (grey, alginate-laden) is inserted into the central inlet and is focused by the side flows containing solution B (orange, ethanol-based). Inversely, for class II fibers, solution B is inserted into the central inlet and is focused by the side flows that, in this case, contain solution A. This is shown in **Figure S31** where we represent, for all scenarios, the streams of solution A and B at the outlet of the device. As expected, because the diffusion coefficient considered for alginate is very low, the two streams are well separated (note that the distributions in **Figure S31** do not represent the transport of Htrz and Fe(II) but rather the fluids containing these species, otherwise there would be interdiffusion). Interestingly, for any given FRR, the size of the focused stream depends on which solution is inserted in the central inlet, and thus, on whether class I or class II fibers are fabricated. This is because solutions A and B have very different viscosity, which influences the width of the focused stream (compared to a more viscous solution, a less viscous solution tends to occupy a smaller cross-section of the device when focused). Specifically, for the same FRR, a more focused central stream is obtained when fabricating class II fibers because that is achieved by inserting the less viscous solution B (orange) through the central inlet. In contrast, a less focused central stream is obtained when fabricating class I fibers because that is achieved by inserting the more viscous solution A (grey) through the central inlet.


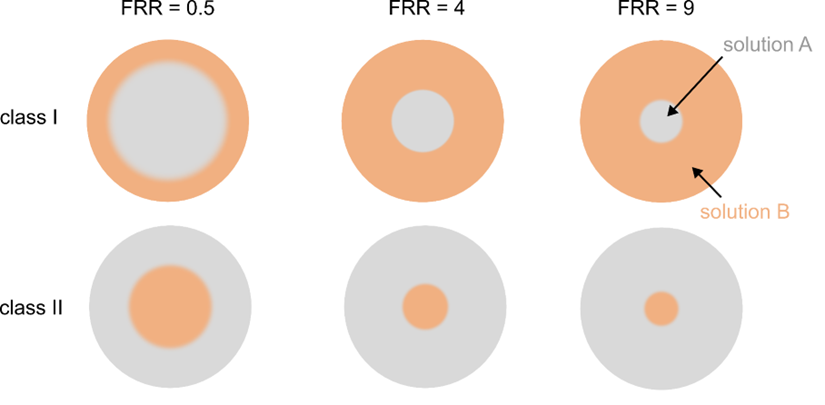


**Figure S31**. Distribution of solutions A and B at the outlet of the microfluidic device obtained from numerical simulations. Solution A is identified as the region where the alginate concentration is >50 % of that at the inlet, whereas solution B corresponds to the remaining fluid.

During the continuous flow of solutions A and B in the microfluidic device, there is transport of solutes by convection (flow focusing) and diffusion (in the radial direction). In **Figure S32** – **Figure S37** we show how the transport of different chemical species evolves along the microfluidic device for class I and class II fibers being fabricated at increasing flow rate ratios (FRR). In each figure, we represent the fraction of alginate solution and the concentration of Htrz and Fe^2+^ (**Figure S32** – **Figure S34** for class I fibers at increasing FRR, **Figure S35** – **Figure S37** for class II fibers at increasing FRR).


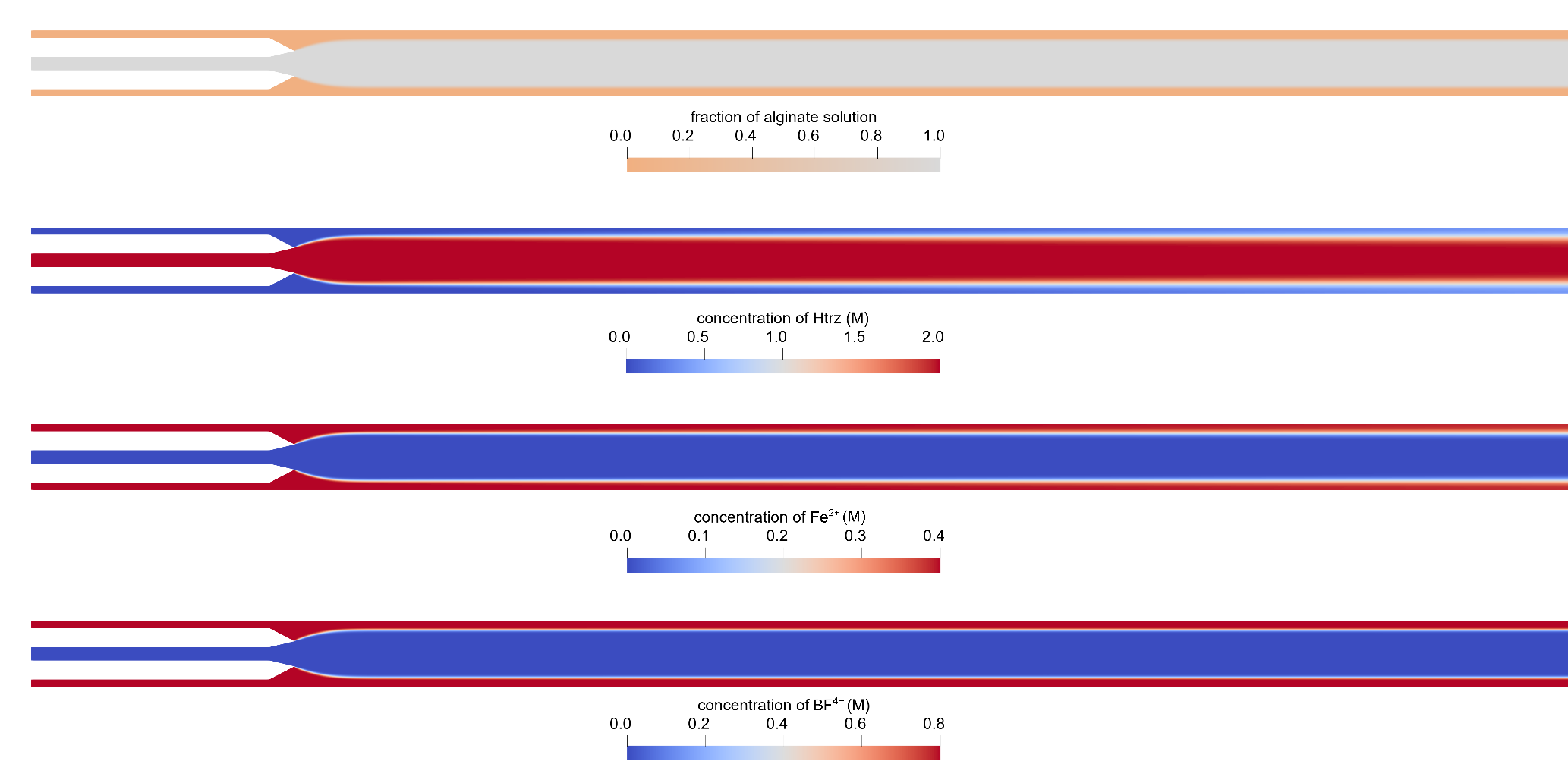


**Figure S32**. Numerical simulation results for class I fiber formation considering FRR = 0.5.


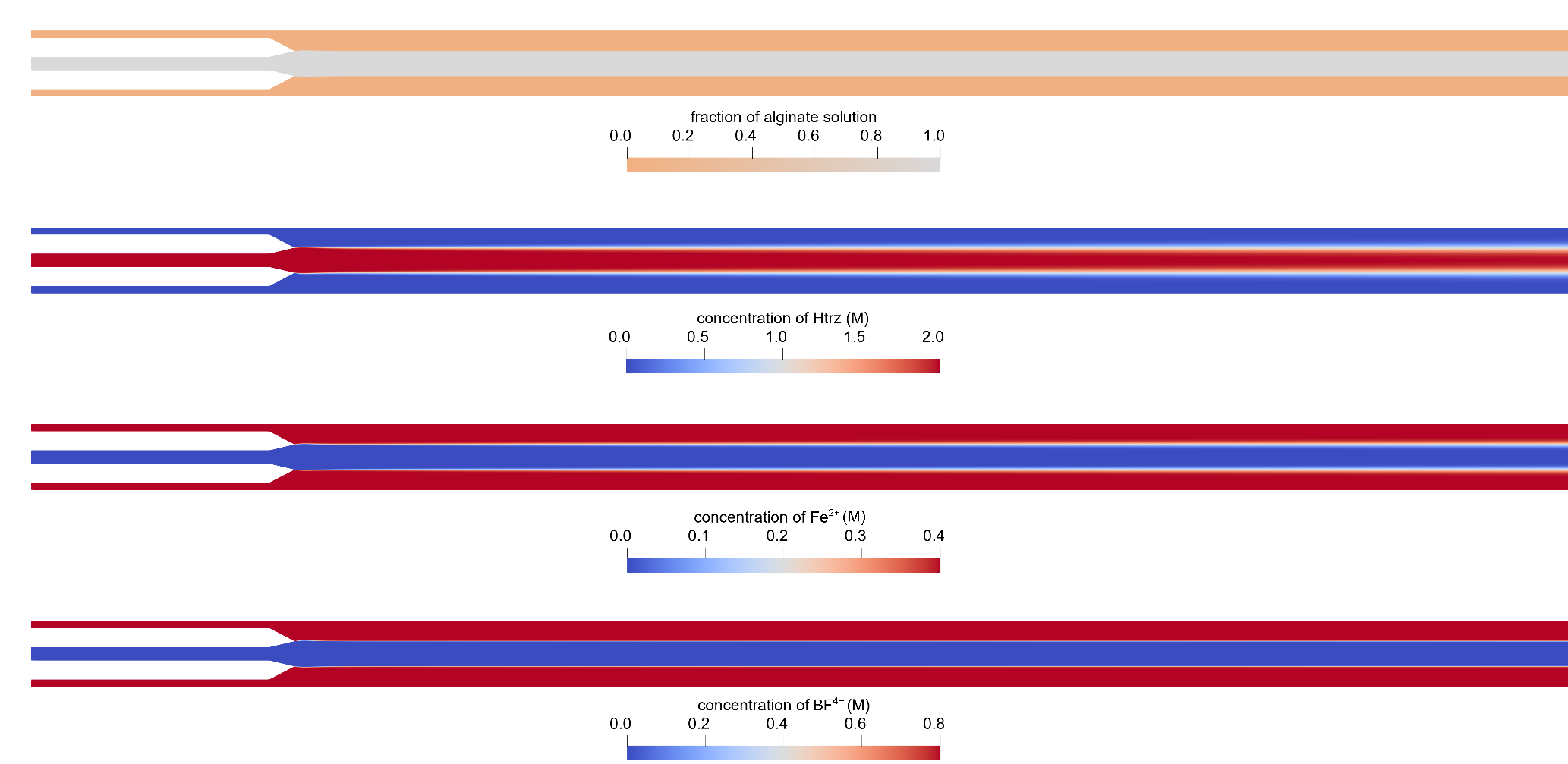


**Figure S33**. Numerical simulation results for class I fiber formation considering FRR = 4.


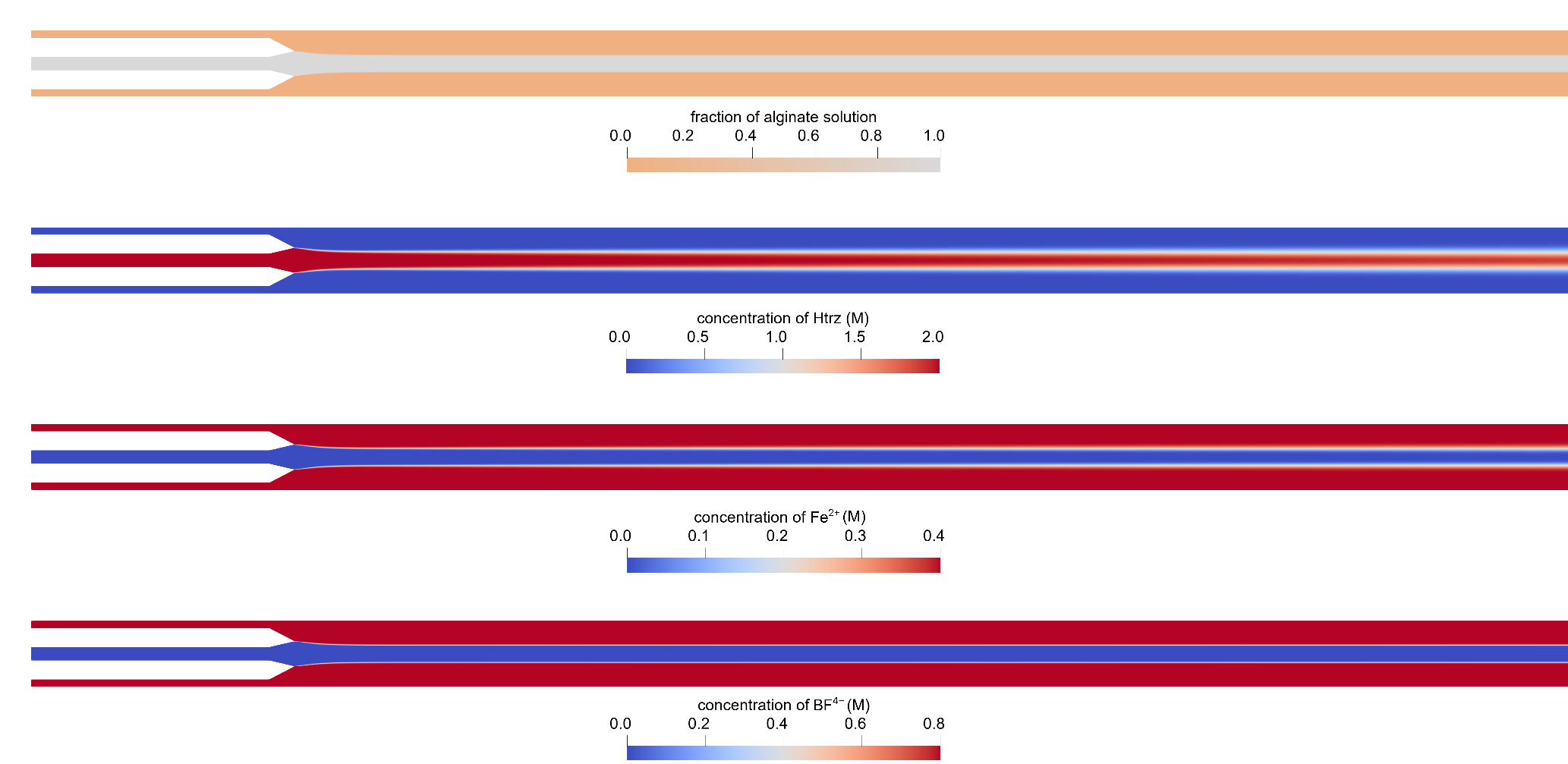


**Figure S34**. Numerical simulation results for class I fiber formation considering FRR = 9.


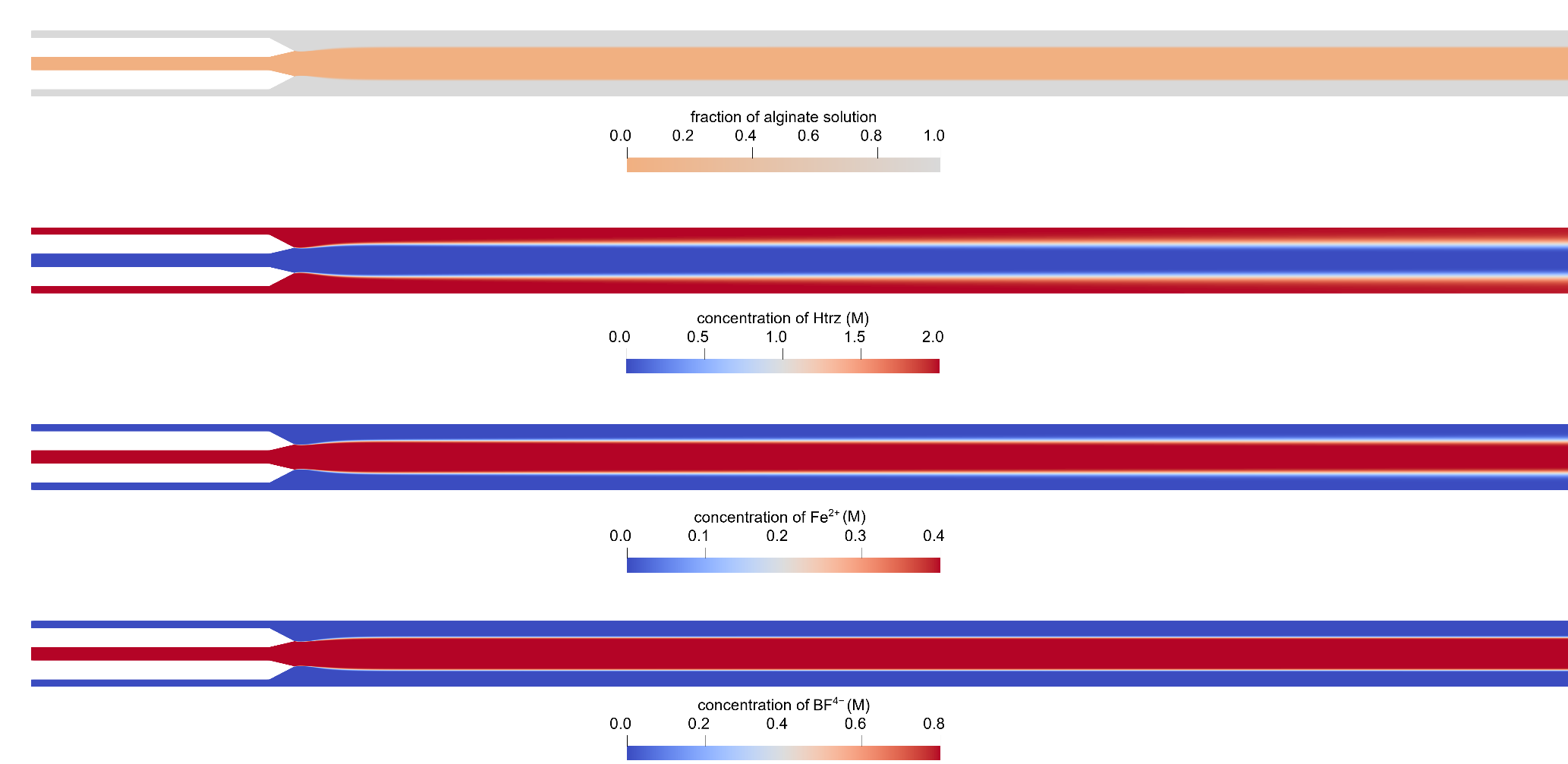


**Figure S35**. Numerical simulation results for class II fiber formation considering FRR = 0.5.


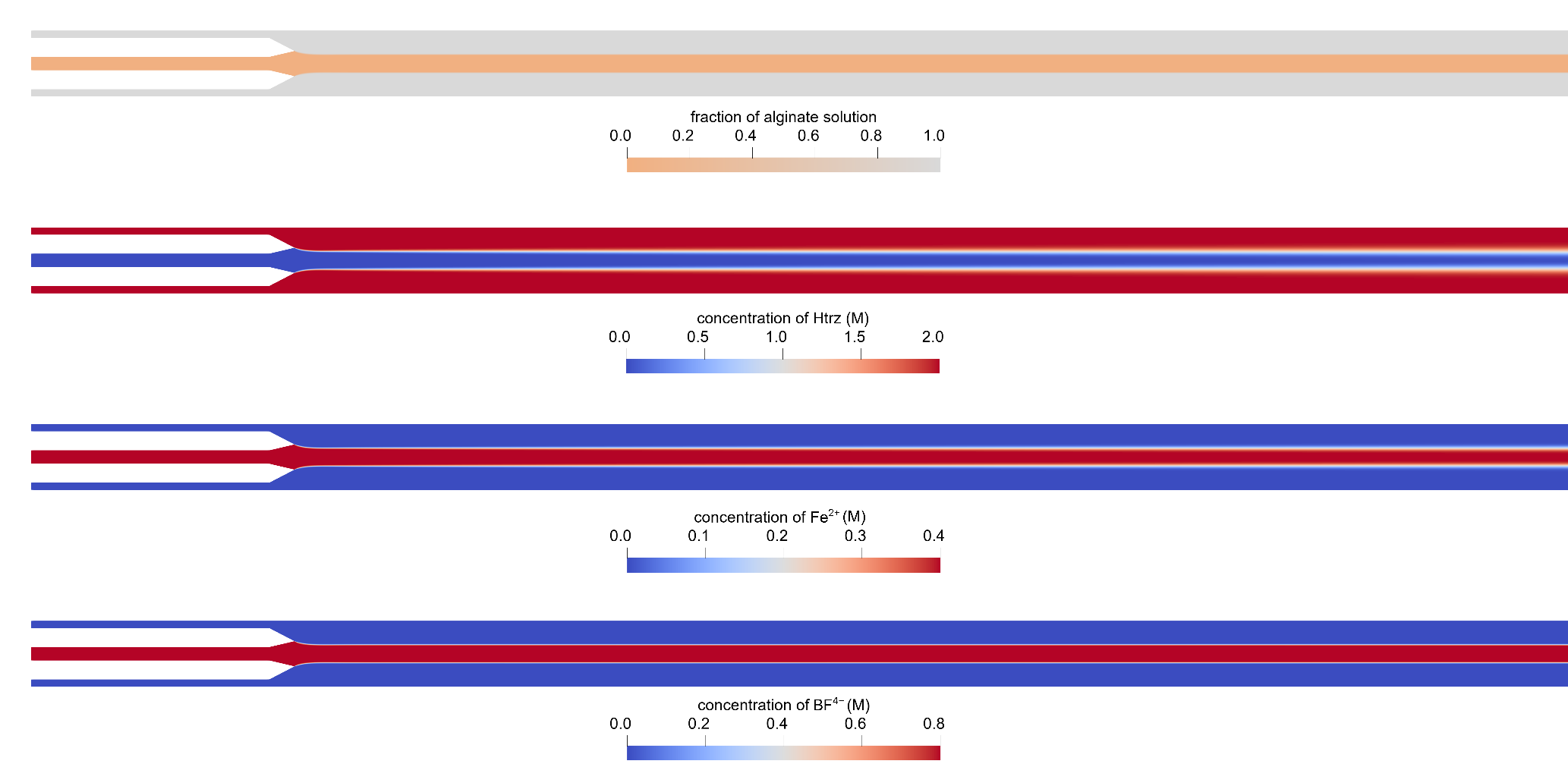


**Figure S36**. Numerical simulation results for class II fiber formation considering FRR = 4.


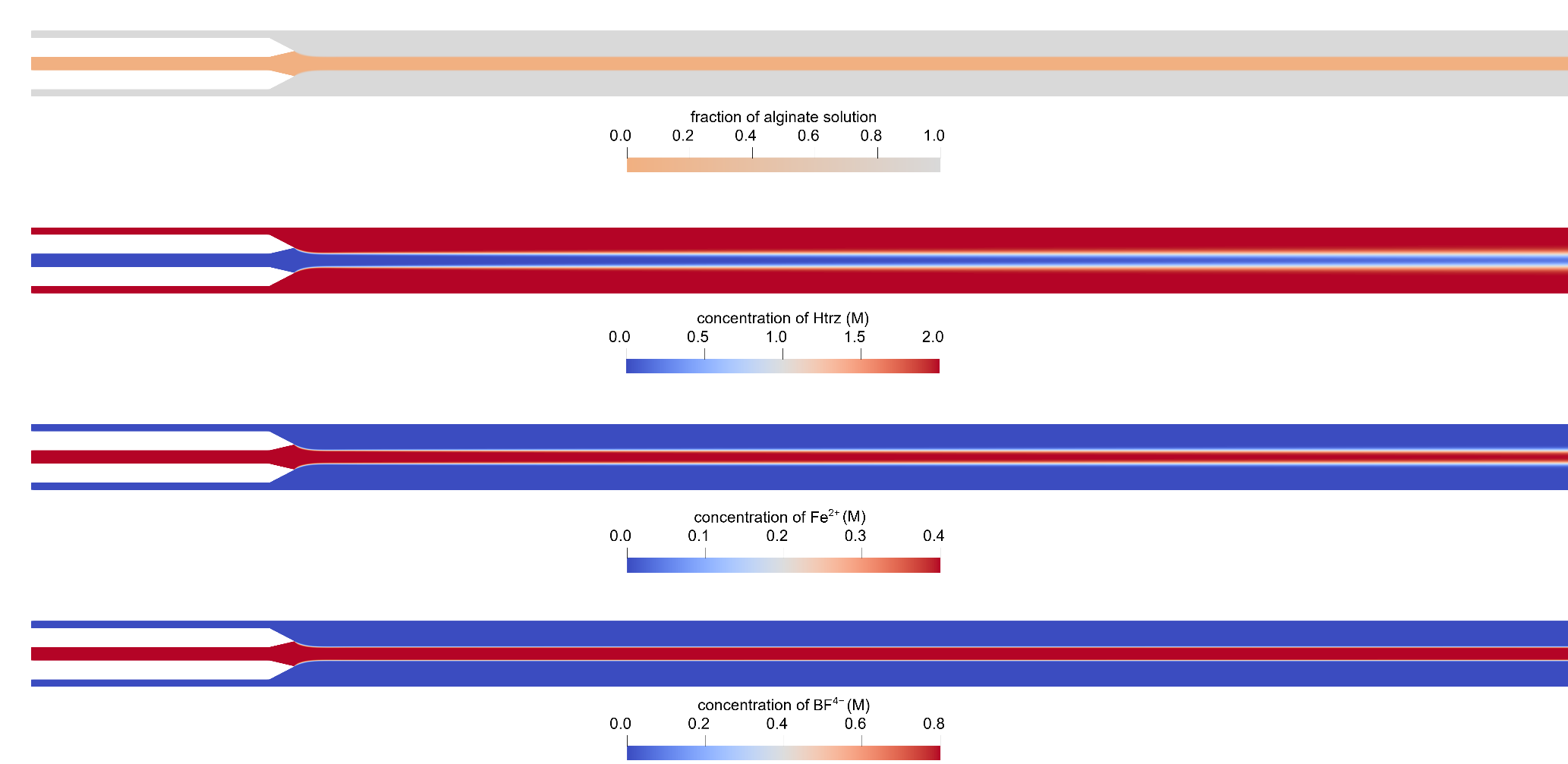


**Figure S37**. Numerical simulation results for class II fiber formation considering FRR = 9.

As expected, results show that solutes are transported radially as the fluids move along the device, and that those in the central stream become more focused with increasing FRR (FRR is increased by increasing the side flow rate at the expense of the central flow rate). Moreover, and as expected, the radial transport of solutes also grows with their diffusion coefficient. For example, Htrz rapidly occupied the entire channel radius because of its high diffusion coefficient.

To investigate how the concentration of solutes influences fiber formation, we defined reaction-diffusion (RD) regions depending on the presence of specific solutes. Chemically, the alginate fiber backbone is formed in the presence of alginate and Fe^2+^, while the formation of spin-crossover particles occurs in the presence of Fe^2+^ and Htrz. Therefore, we considered that the RD region in which alginate fiber backbone formation occurs (referred to as RD backbone) is the region within solution A (i.e. fraction of alginate solution > 0.5) where the concentration of Fe^2+^ was > 1 mM. Simultaneously, we considered that the RD region for spin-crossover particles formation (referred to as RD particles) is the region in which the concentrations of Htrz and Fe^2+^ were > 1 mM. This threshold of 1 mM was chosen to enable the visualization of the RD regions, and we confirmed that considering different threshold values would not significantly affect the results. Finally, because the fibers are composite materials made of the backbone and spin-crossover particles, we considered a RD region that constitutes the composite (referred to as RD composite). This RD region forms where the fraction of alginate solution is > 0.5, the concentration of Fe^2+^ is > 1 mM and the concentration of Htrz is > 1 mM (in other words, the RD composite is the region in the device simultaneously occupied by both RD backbone and RD particles).

In the following figures we represented RD backbone in blue, RD particles in red and RD composite in purple. Note that the RD regions are significantly different when fabricating class I and class II fibers, due to the different insertion position of solutions A and B (and their solutes; **Figure S38** vs **Figure S39**).


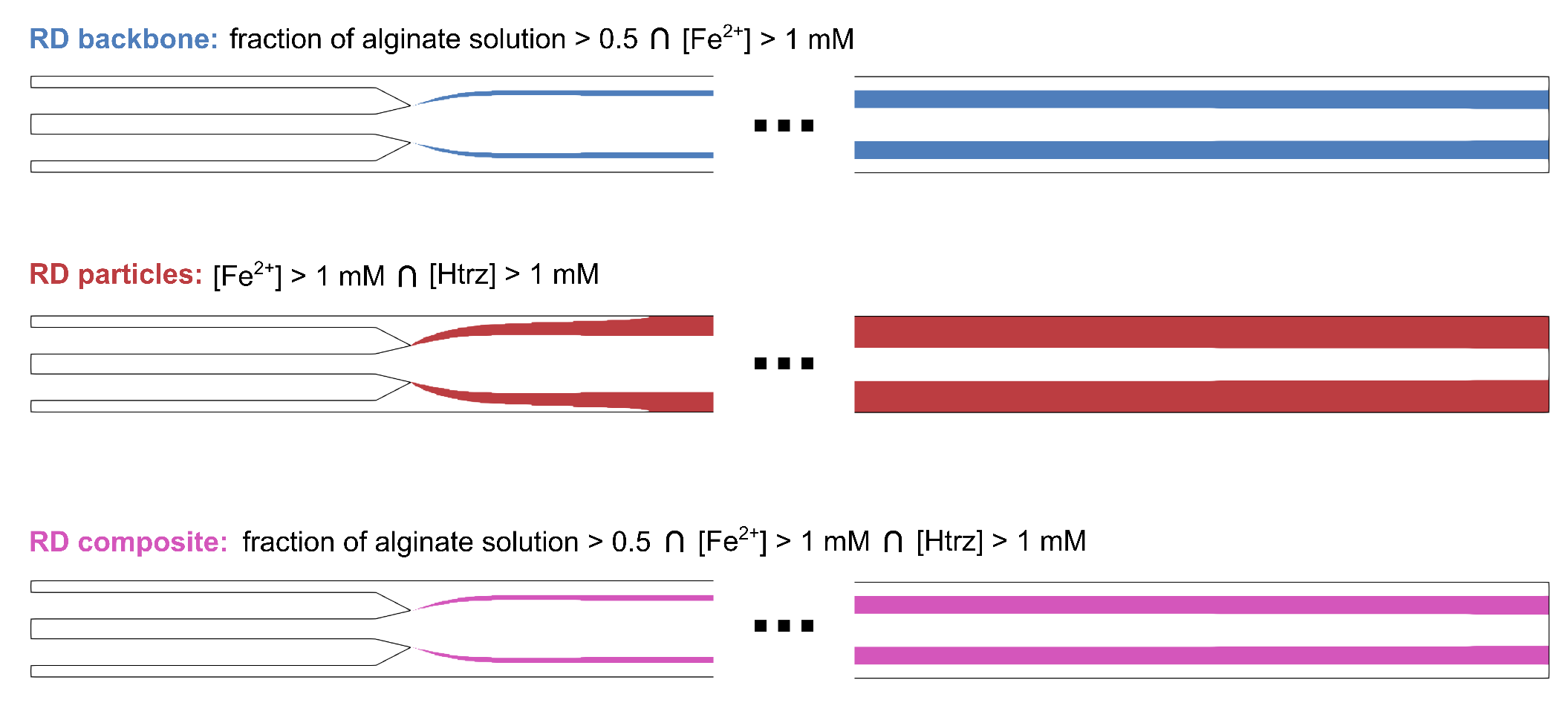


**Figure S38**. Representation of the RD regions (RD backbone in blue, RD particles in red and RD composite in purple) of the microfluidic device generating a class I fiber at FRR = 0.5.


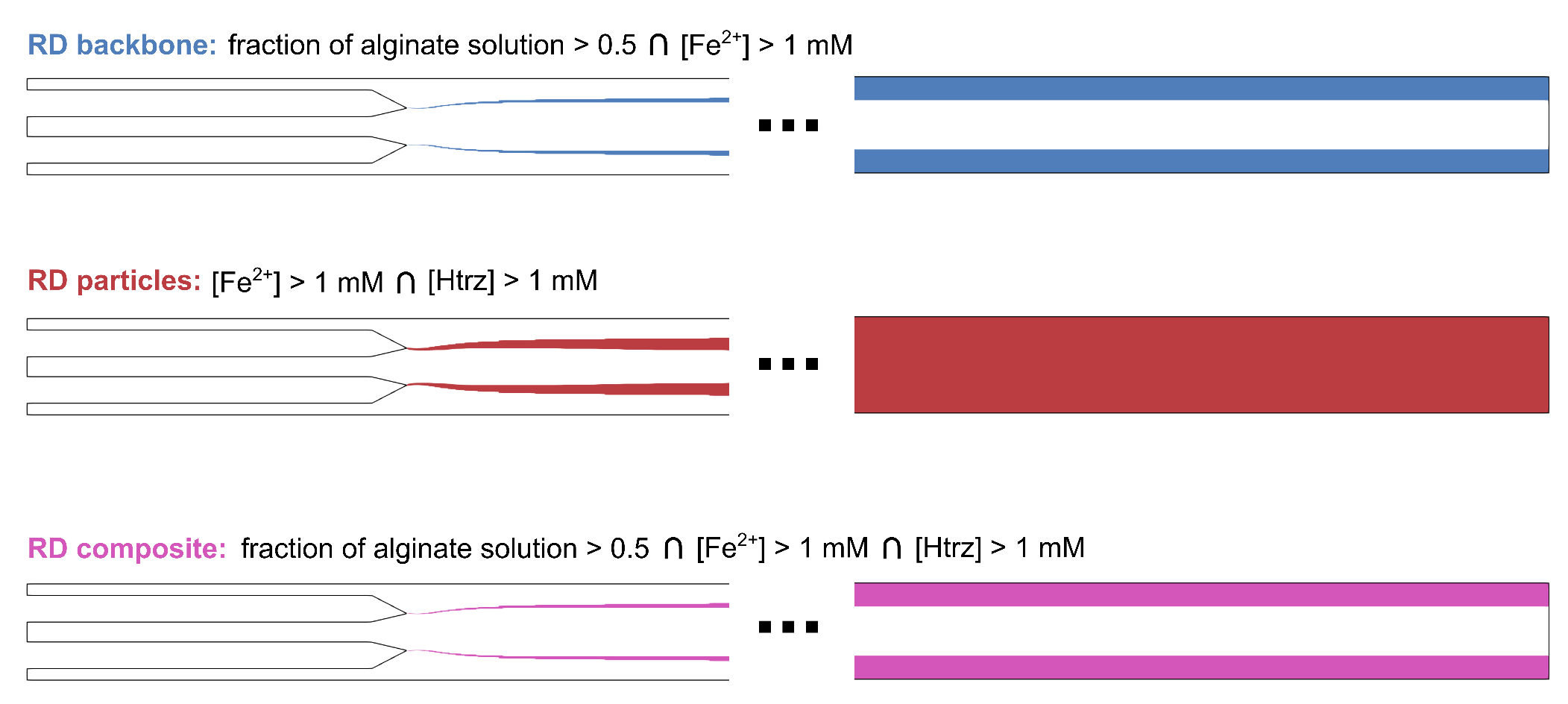


**Figure S39**. Representation of the RD regions (RD backbone in blue, RD particles in red and RD composite in purple) of the microfluidic device generating a class II fiber at FRR = 0.5.

As mentioned above, the RD regions grow along the device due to the diffusion of species (**Figure S38**, **Figure S39**). To better understand the generation of the different RD regions at various FRR, we marked in **Figure S40** (for class I) and in **Figure S41** (for class II) where each chemical species and RD region are located, along the radius of the channel outlet. Specifically, we marked:

- the regions along the radius where solution A and solution B are located (grey/orange);
- the regions along the radius where the concentrations of Htrz and Fe^2+^ are above the minimum threshold (1 mM) considered for acknowledging a RD region;
- the regions along the radius where RD regions are located: RD backbone (blue) and RD particles (red).


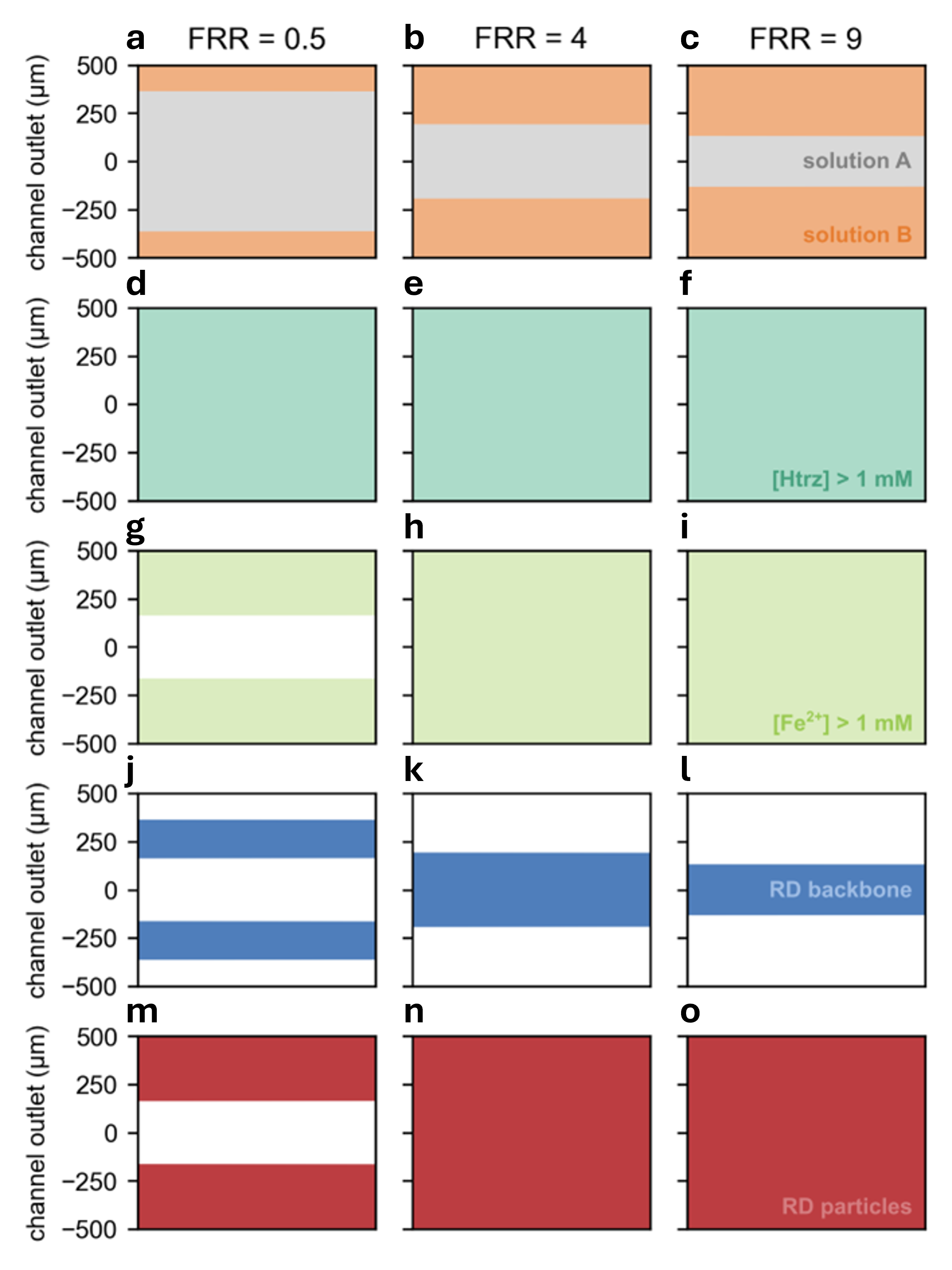


**Figure S40**. Positions along the radius of the channel outlet where each chemical species and RD region are located for class I fiber formation: solution A/solution B, [Htrz] > 1 mM, [Fe^2+^] > 1 mM, RD backbone and RD particles.


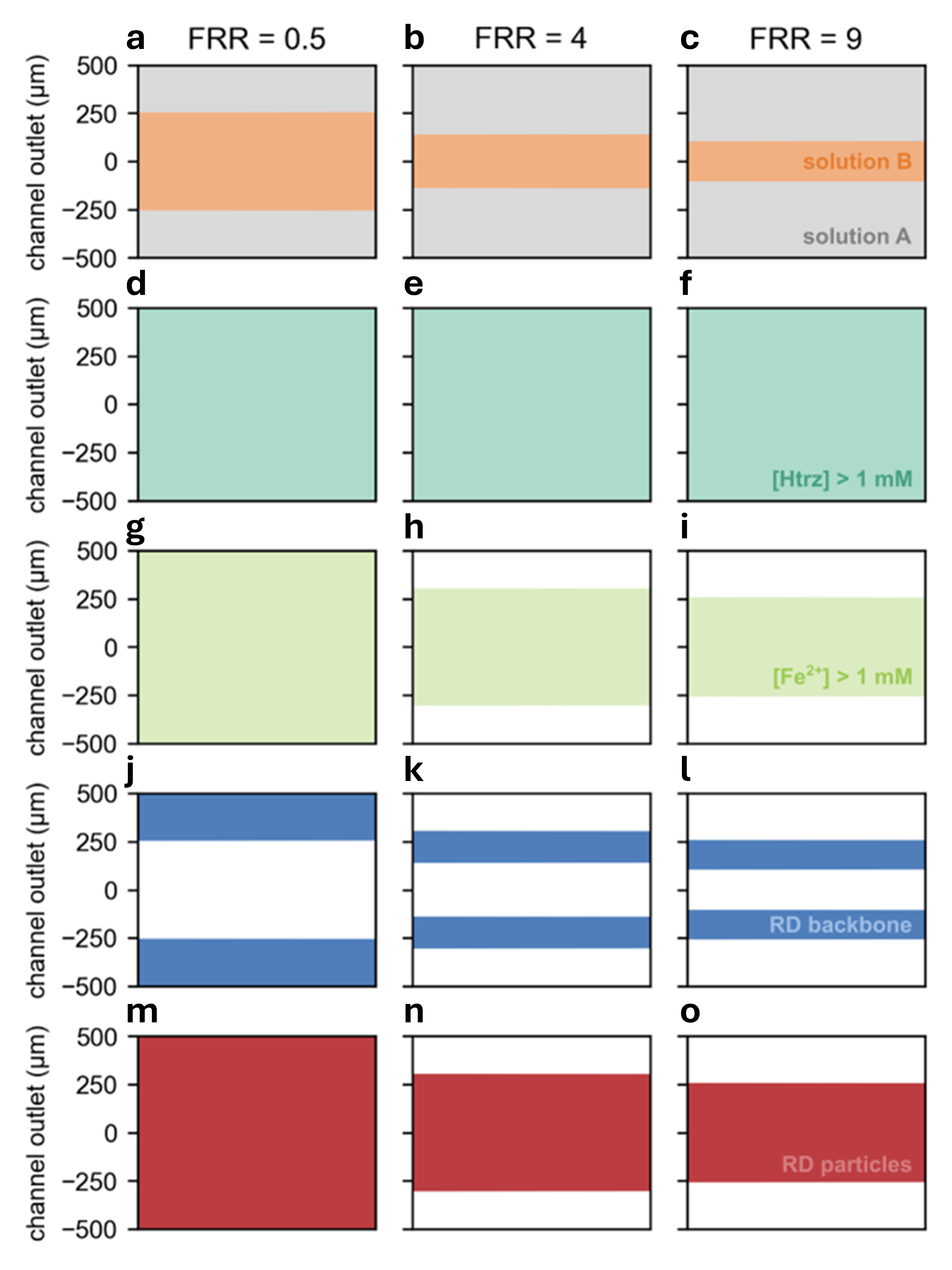


**Figure S41**. Positions along the radius of the channel outlet where each chemical species and RD region are located for class II fiber formation: solution A/solution B, [Htrz] > 1 mM, [Fe^2+^] > 1 mM, RD backbone and RD particles.

**Figure S40** presents the main results for class I fiber formation. In that type of flow, solution A (alginate-laden) is inserted into the central inlet and Htrz, the solute in solution A, diffuses rapidly from the center to the outer wall, occupying the entire channel cross-section at the outlet (with a concentration > 1 mM), for all FRRs. Simultaneously, solution B is inserted into the side inlets and Fe^2+^ diffuses radially towards the center of the device. This solute occupies a larger portion of the radius at the outlet for high FRRs (e.g. 9) because FRR is increased by increasing the flow rate of the side stream at the expense of that of the central stream. Yet, Fe(II) is not restricted to the region where solution B is, given that it diffuses into the region occupied by solution A (**Figure S40**). This means that both convection (flow focusing) and diffusion are important to the mass transport of solutes occurring in the device. Finally, the RD regions shown in the figure, i.e. RD backbone and RD particles, correspond to the regions where there is overlap (or simultaneous presence) of the relevant species: RD backbone is the region within solution A where [Fe^2+^] > 1 mM; RD particles is the region where [Htrz] > 1 mM and [Fe^2+^] > 1 mM. For RD backbone, an increasing FRR increases the focusing of solution A while leading to Fe^2+^ occupying the entire cross-section of the device at the outlet (**Figure S40**). This suggests that, at high FRR, the RD backbone is limited by the presence of solution A (alginate), whereas for low FRR, it is limited by the presence of Fe^2+^. For RD particles, Htrz was found to occupy the entire channel cross-section at the outlet for all the FRRs tested, whereas the same is only true at high FRRs for Fe^2+^ (**Figure S40**). This suggests that the location of RD particles will correspond to the regions where Fe^2+^ is present (being thus limited by its diffusion rate).

**Figure S41** shows the same type of results as **Figure S40** but for class II fiber formation. In this type of flow, solution A (alginate-laden) is now inserted into the side inlets and Htrz diffuses rapidly towards the center of the device, occupying the entire channel cross-section at the outlet (with a concentration >1 mM), for all FRRs. Simultaneously, solution B is inserted into the central inlet, with Fe^2+^ diffusing from the center to the outer walls. Much like the region occupied by Fe^2+^ widened with increasing FRRs in class I fiber formation when it was inserted through the side inlets, the region occupied by Fe^2+^ in class II fiber formation got narrower as FRRs increased, because Fe^2+^ is inserted into the central inlet and thus became focused. Despite these differences, the formation of the RD regions appears to follow the same principles that were observed for class I fibers: the RD backbone is limited by the presence of solution A (alginate) at low FRR and by the presence of Fe^2+^ at high FRR (in class I fiber formation the reverse was observed), whereas RD particles correspond to the regions where Fe^2+^ is present.

We represented RD composite in class I and class II fiber formation at increasing flow rate ratios in **Figure S42**. The RD composite is the region of the device where RD backbone and RD particles are simultaneously present. Because RD backbone was always smaller than, and within, RD particles(**Figure S40** and **Figure S41**), the region occupied by RD composite was that of RD backbone. Furthermore, in **Figure S43** we represent the RD composite at the outlet of the device in class I and class II fiber formation at increasing flow rate ratios along with solutions A and B. Depending on the conditions and type of fiber, the shape of the RD composite will be different, which may influence its properties.


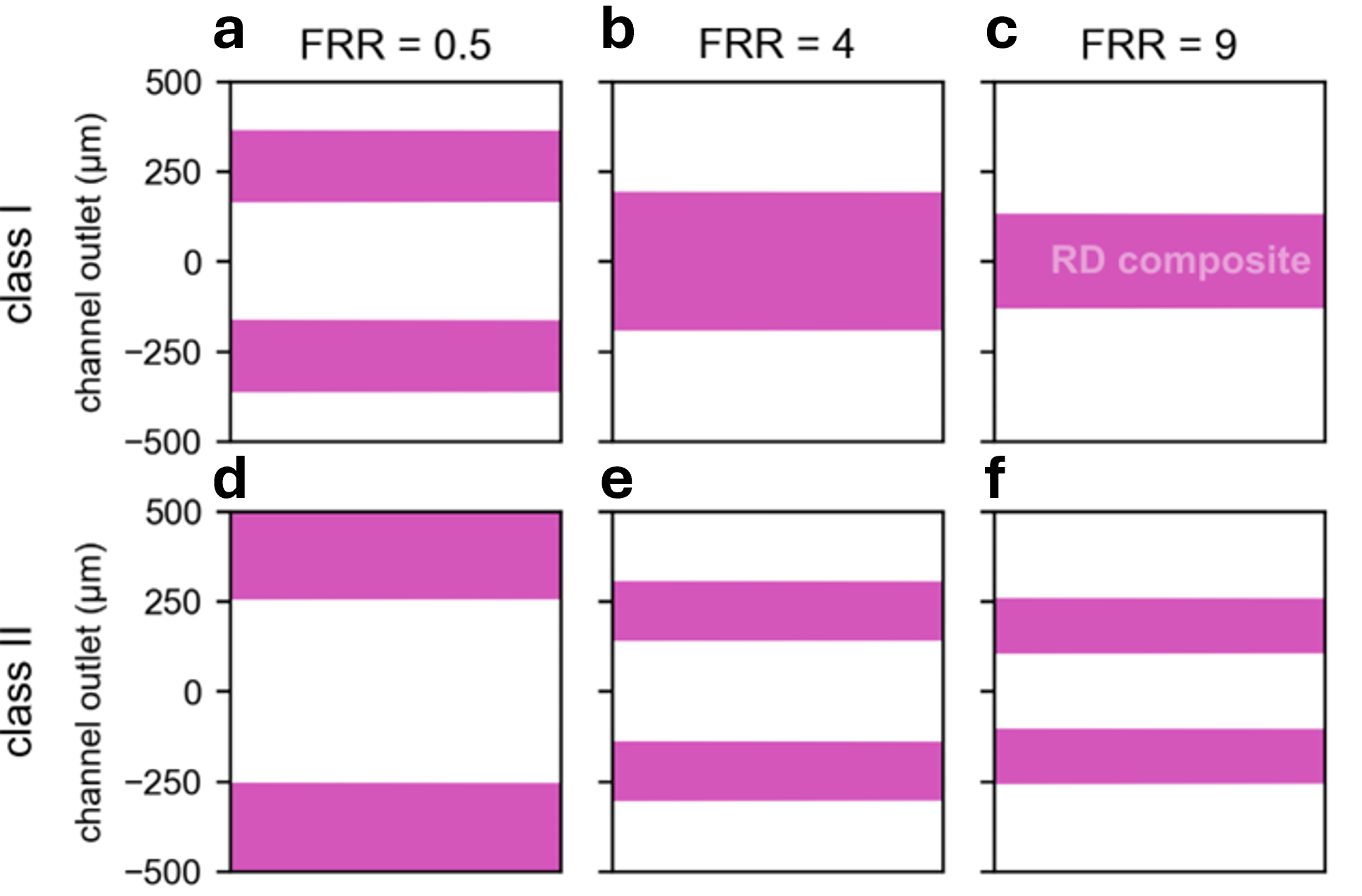


**Figure S42**. RD composite at the outlet of the microfluidic device for class I and class II fibers generated at various flow rate ratios.


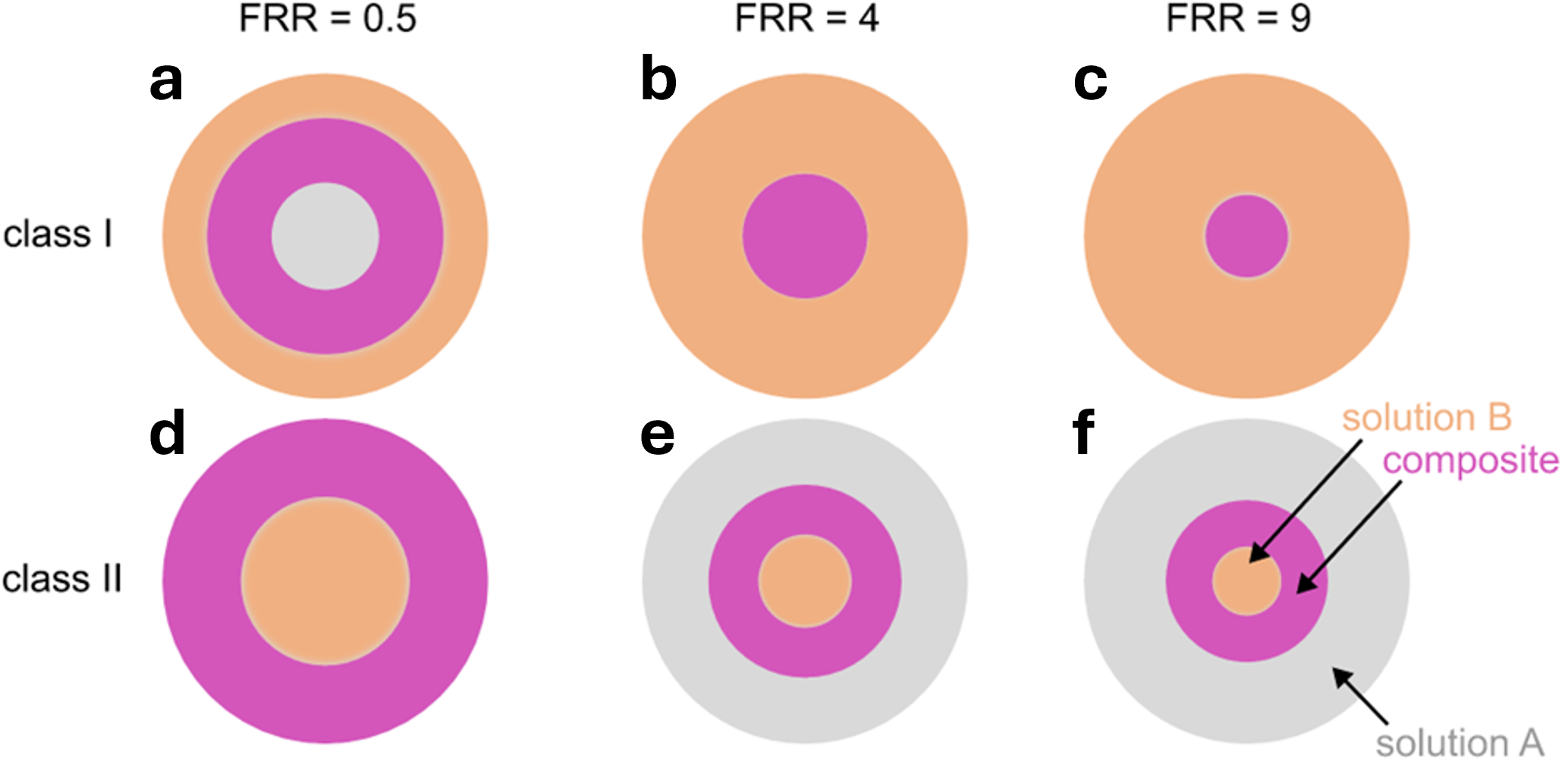


**Figure S43**. Representation of RD composite and solutions A and B at the outlet of the microfluidic device obtained from numerical simulations.

In **Figure S43**, we show the width of each RD region at the outlet of the device for class I and class II fiber formation, for increasing FRR. The RD widths, and how they change with FRR, can be directly understood from the earlier discussion on the factors governing the formation of each RD regions. The RD backbone (blue) is limited by the position of both solution A and Fe^2+^ (with the one inserted in the central inlet being limiting at high FRRs, and the other at low FRRs). Given that either one of these reactants is always at the central (focused) stream for class I and class II fiber formation, the width of the RD backbone is seen decreasing with FRR regardless of fiber class. In contrast, the RD particles (red) is limited by Fe^2+^ in both class I and class II fiber formation and, therefore, the width of the RD particles will depend directly on how Fe^2+^ is transported. In class I fiber formation, Fe^2+^ is inserted laterally and therefore occupies a larger region of the channel outlet at high FRRs (**Figure S40**), leading to the increase in the width of the RD particles that is observed at increasing FRRs (**Figure S44**). Inversely, in class II fiber formation, Fe^2+^ is inserted through the central inlet and therefore it becomes focused at high FRRs (**Figure S41**), which is consistent with the fact that the width of the RD particles decreases with FRR (**Figure S44**). Finally, the width of the RD composite is exactly equal to the width of the RD backbone in all conditions because these occupy the same region (as discussed above).


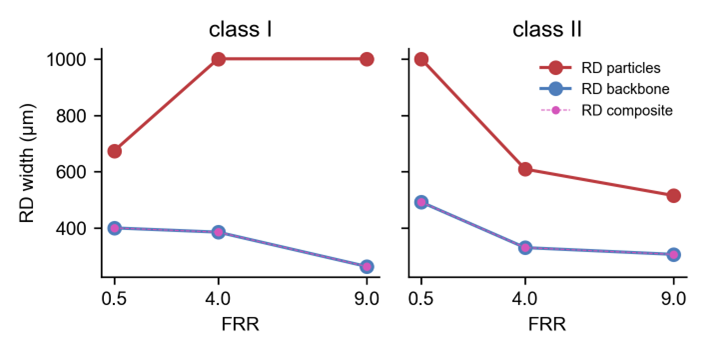


**Figure S44**. RD width in the generation of class I and class II fibers using various flow rate ratios.

In **Figure S45** we plotted the width of the RD regions, now along the length of the microfluidic device, for class I and II fiber formation under various FRRs. In this figure, the arrows indicate the solute(s) responsible for driving the growth of each RD region at different positions along the length of the device.

During the formation of RD particles (red), RD width initially increases rapidly due to the simultaneous diffusion of Htrz and Fe^2+^. As we move along the length of the device, Htrz quickly occupies the entire channel due to having a high diffusion coefficient. From that point onwards, the rate of growth of the RD width decreases, given that the growth becomes driven only by Fe^2+^ diffusion. Finally, in certain cases where the entire channel becomes occupied by Fe^2+^ (e.g. class I fiber formation using FRR = 9), the region of RD particles becomes that of the entire channel (1000 µm). Note how the rate at which the RD grows will greatly depend on the FRR that is used owing to different degrees of focusing of the central stream influencing the diffusion of species.

During the formation of RD backbone (blue), the width of the RD grows only due to Fe^2+^ diffusion given that alginate does not diffuse along the channel radius. This implies that the width of the RD backbone stops increasing when Fe^2+^ occupies the region where alginate is. At that point, a plateau in RD width is reached, with it being equal to the size of the alginate stream (solution A). Finally, the width of the RD composite (pink) evolves exactly like that of the RD backbone and is equal to it at every position along the channel length (as discussed above).


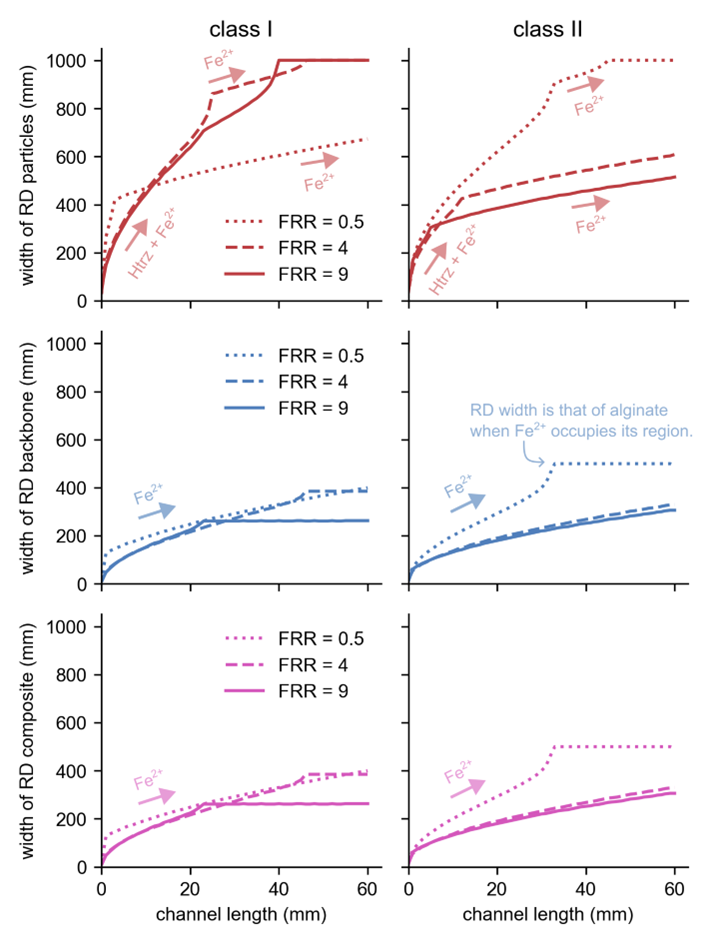


**Figure S45**. Width of the various RD regions along the channel length in the generation of class I and class II fibers using various flow rate ratios. The arrows indicate the solutes whose diffusion is driving the increase in RD width at different positions along the length of the channel.

# References

[1] J. Kroeber, J.-P. Audiere, R. Claude, E. Codjovi, O. Kahn, J. G. Haasnoot, F. Groliere, C. Jay, A. Bousseksou, *Chem. Mater.* **1994**, *6*, 1404–1412.

[2] A. Michalowicz, J. Moscovici, B. Ducourant, D. Cracco, O. Kahn, *Chem. Mater.* **1995**, *7*, 1833–1842.

[3] L. C. S. Nunes, *Opt. Lasers Eng.* **2011**, *49*, 640–646.

[4] K. Mazur, A. Gądek-Moszczak, A. Liber-Kneć, S. Kuciel, *Materials (Basel).* **2021**, *14*, 1712.

[5] T. R. Cuadros, O. Skurtys, J. M. Aguilera, *Carbohydr. Polym.* **2012**, *89*, 1198–1206.

[6] Z. Wang, A. A. Volinsky, N. D. Gallant, *J. Appl. Polym. Sci.* **2014**, *131*, DOI https://doi.org/10.1002/app.41050.

[7] Mizerski, Andrzej, *MATEC Web Conf.* **2018**, *247*, 64.

[8] S. V Lokesh, A. K. Satpati, B. S. Sherigara, *Open Electrochem. J.* **2010**, *2*.

[9] P. Yan, W. Lan, J. Xie, *Trends Food Sci. Technol.* **2024**, *143*, 104217.

[10] D. L. Parkhurst, C. A. J. Appelo, *Batch-Reaction, One-Dimensional Transp. Inverse Geochemical Calc. (Denver, Color. USA, US Geol. Surv. Water Resour. Div.* **2013**.

[11] R. B. Bird, W. E. Stewart, E. N. Lightfoot, *Transport Phenomena*, John Wiley & Sons, New York, **1960**.
